# Neurodevelopmental Outcomes After Nitric Oxide During Cardiopulmonary Bypass for Open Heart Surgery: A Randomized Clinical Trial

**DOI:** 10.1001/jamanetworkopen.2024.58040

**Published:** 2025-02-05

**Authors:** Debbie A. Long, Kristen S. Gibbons, Stephen B. Horton, Kerry Johnson, David H. F. Buckley, Simon Erickson, Marino Festa, Yves d’Udekem, Nelson Alphonso, Renate Le Marsney, David S. Winlaw, Kate Masterson, Kim van Loon, Paul J. Young, Andreas Schibler, Luregn J. Schlapbach, Warwick Butt

**Affiliations:** 1 School of Nursing, Centre for Healthcare Transformation, Queensland University of Technology, Brisbane, Australia; 2 Paediatric Intensive Care Unit, Queensland Children’s Hospital, Children’s Health Queensland, Brisbane, Australia; 3 Children’s Intensive Care Research Program, Child Health Research Centre, The University of Queensland, Brisbane, Australia; 4 Cardiac Surgical Unit, Royal Children’s Hospital, Melbourne, Victoria, Australia; 5 Faculty of Medicine, Department of Paediatrics, University of Melbourne, Melbourne, Victoria, Australia; 6 Clinical Sciences Theme, Murdoch Children’s Research Institute, Melbourne, Victoria, Australia; 7 Paediatric Intensive Care Unit, Starship Children’s Hospital, Auckland, New Zealand; 8 Paediatric Critical Care, Perth Children’s Hospital, Western Australia and The University of Western Australia, Crawley, Australia; 9 Kids Critical Care Research, Paediatric Intensive Care Unit, Children’s Hospital at Westmead, Westmead, New South Wales, Australia; 10 Sydney Children’s Hospital Network, Sydney, New South Wales, Australia; 11 Children’s National Hospital and The George Washington University School of Medicine and Health Sciences, Washington, District of Columbia; 12 Cardiac Surgery, Queensland Children’s Hospital, Brisbane, Australia; 13 School of Medicine, Children’s Health Clinical Unit, The University of Queensland, Brisbane, Australia; 14 Heart Centre for Children, The Children’s Hospital at Westmead, Westmead, New South Wales, Australia; 15 Sydney Children’s Hospital Network and Faculty of Medicine and Health, University of Sydney, Sydney, New South Wales, Australia; 16 Paediatric Intensive Care Unit, Royal Children’s Hospital Melbourne, Melbourne, Victoria, Australia; 17 Department of Anaesthesiology, University Medical Center Utrecht, Wilhelmina Children’s Hospital, Utrecht, the Netherlands; 18 Intensive Care Unit, Wellington Hospital, Wellington, New Zealand; 19 Medical Research Institute of New Zealand, Wellington, New Zealand; 20 Australian and New Zealand Intensive Care Research Centre, Monash University, Melbourne, Victoria, Australia; 21 Department of Critical Care, University of Melbourne, Melbourne, Victoria, Australia; 22 James Cook University, Townsville, Queensland, Australia; 23 Critical Care Research Group, Wesley Medical Research, St Andrew’s War Memorial Hospital, Brisbane, Queensland, Australia; 24 Department of Intensive Care and Neonatology, University Children’s Hospital Zurich, University of Zurich, Zurich, Switzerland; 25 Children’s Research Center, University Children’s Hospital Zurich, University of Zurich, Zurich, Switzerland; 26 Central Clinical School, Faculty of Medicine, Monash University, Melbourne, Victoria, Australia

## Abstract

**Importance:**

Children with congenital heart defects who undergo cardiopulmonary bypass (CPB) surgery are at risk for delayed or impaired neurodevelopmental outcomes. Nitric oxide (NO) added to the CPB oxygenator may reduce systemic inflammation due to CPB and improve recovery from surgery, including improved neurodevelopmental outcomes.

**Objective:**

To investigate neurodevelopment, health-related quality of life (HRQOL), and factors associated with impaired neurodevelopment at 12 months post surgery in infants who received CPB with NO or standard CPB.

**Design, Setting, and Participants:**

This double-masked randomized clinical trial was conducted in 6 centers in Australia, New Zealand, and the Netherlands between July 19, 2017, and April 28, 2021, with a preplanned prospective follow-up 12 months postrandomization completed on August 5, 2022. The cohort included 1364 infants younger than 2 years who underwent open heart surgery with CPB for congenital heart disease.

**Interventions:**

The intervention group received NO 20 ppm into the CPB oxygenator. The control group received standard CPB.

**Main Outcomes and Measures:**

The primary outcome was neurodevelopment, defined as the Ages and Stages Questionnaire, Third Edition (ASQ-3) total score. Secondary outcomes were HRQOL and functional status as measured by Pediatric Quality of Life Inventory and modified Pediatric Overall Performance Category scores, respectively. Sensitivity analyses modeled the outcome for patients lost to follow-up.

**Results:**

Of 1318 infants alive 12 months after randomization, follow-up was performed in 927, with 462 patients in the NO group and 465 in the standard care group (median [IQR] age at follow-up, 16.6 [13.7-19.8] months; median [IQR] time since randomization, 12.7 [12.1-13.9] months; 516 male [55.7%]). There were no differences between the NO and standard care groups in ASQ-3 total score (mean [SD], 196.6 [75.4] vs 198.7 [73.8], respectively; adjusted mean difference, −2.24; 95% CI, −11.84 to 7.36). There were no differences in secondary outcomes. Prematurity (gestational age <37 weeks), univentricular lesions, congenital syndromes, and longer intensive care unit length of stay were associated with lower ASQ-3 total scores in adjusted multivariable analyses.

**Conclusions and Relevance:**

In this randomized clinical trial of infants with congenital heart disease, NO administered via the CPB oxygenator did not improve neurodevelopmental outcomes or HRQOL 12 months after open heart surgery. Further research should explore homogenous cohorts with higher surgical risk and higher-dose or alternative therapies.

**Trial Registration:**

ANZCTR Identifier: ACTRN12617000821392

## Key Points

**Question:**

Does nitric oxide (NO) administered via cardiopulmonary bypass (CPB) improve neurodevelopment and health-related quality of life (HRQOL) in infants undergoing open heart surgery for congenital heart disease?

**Findings:**

In this secondary analysis of a randomized clinical trial of 1364 infants randomized to receive NO 20 ppm via CPB or standard CPB, parent-rated neurodevelopmental and HRQOL outcomes did not differ 12 months post surgery.

**Meaning:**

These findings suggest that administering NO during CPB does not improve neurodevelopmental or HRQOL outcomes, highlighting the need for further exploration in a homogenous cohort and of higher-dose or alternative therapies.

## Teaser

This randomized clinical trial compares 12-month neurodevelopmental and quality-of-life outcomes in infants receiving nitric oxide vs standard care during cardiopulmonary bypass surgery.

## Introduction

Advances in prenatal diagnosis, perioperative management, and postoperative care have dramatically improved outcomes for children with congenital heart disease (CHD) in recent decades. However, as increasing numbers of infants and young children survive their initial cardiac operation, substantial neurodevelopmental morbidities continue to complicate long-term outcomes in this population.^1^

While the underlying pathophysiologic mechanisms of CHD-associated neurologic impairment remain complex, ranging from genetic and intrauterine to postnatal conditions and environmental factors, including social determinants of health, the contribution of intraoperative and early postoperative factors are potentially modifiable.^2^ Cardiopulmonary bypass (CPB) is necessary to ensure intraoperative organ perfusion and oxygen supply during most heart surgeries but results in extensive perturbations of host homeostasis across inflammatory, coagulation, and endothelial systems.^3^ Postoperative low cardiac output may compromise brain perfusion and further increase the risk of brain injury. Given the known association of CHD with long-term outcomes, there is a need for novel strategies to improve outcomes.

Nitric oxide (NO) is a promising candidate for modulating biological responses during CPB and subsequent low cardiac output syndrome and might thereby improve brain perfusion and reduce the risk of brain injury.^4,5^ In the international, multicenter, double-masked Nitric Oxide During Cardiopulmonary Bypass to Improve Recovery in Infants With Congenital Heart Defects (NITRIC) randomized clinical trial (RCT) of infants undergoing open heart surgery for CHD, NO administered via CPB did not show a significant reduction in ventilator-free days or a number of secondary early outcomes.^6^ In this follow-up study, we report neurodevelopmental and health-related quality-of-life (HRQOL) outcomes measured 12 months post surgery in the NITRIC trial.

## Methods

This preplanned prospective follow-up study of infants and young children (aged <2 years) randomized to receive NO 20 ppm via CPB or standard CPB in the NITRIC RCT was conducted at 6 pediatric heart centers in Australia, New Zealand, and the Netherlands between July 19, 2017, and April 28, 2021, with the 12-month follow-up completed August 5, 2022.^6,7^ All participating sites had human research ethics committee approval for the study. Parents provided written informed consent prior to enrollment of their child, including consent for the 12-month follow-up. The study is reported according to the Consolidated Standards for Reporting Trials–Patient Reported Outcomes (CONSORT-PRO) checklist for inclusion of patient-reported outcomes in clinical trials.^8^

The full details of the trial protocol and the main trial findings on early outcomes have been published previously and are provided in Supplement 1.^7,9^ Detailed inclusion and exclusion criteria have been described previously.^6^ For the present preplanned analysis, parents of children who died prior to 12 months post randomization were not invited to participate in the follow-up study.

Approaching 12 months post randomization, participant medical records were reviewed for survival status. One primary caregiver per child was invited to complete an online questionnaire assessing neurodevelopment and HRQOL. Medical history since surgery along with socioeconomic data were also collected via the online questionnaire. Race and ethnicity (Aboriginal or Torres Strait Islander, Asian, Māori or Pacific Islander Peoples, White, multiethnic, or other) were collected to describe the cohort of participants, particularly focusing on groups at increased risk, and were self-reported by the child’s parent. Questionnaires were emailed to the primary caregiver via a personalized link to the electronic study database (developed using REDCap [Vanderbilt University])^10,11^ hosted by The University of Queensland and remained open for 3 months. Reminder telephone calls were made and emails sent weekly on 5 occasions if the questionnaire was not commenced or incomplete. After this point, participants were considered lost to follow-up. Parents and study staff remained unaware of the treatment allocation.

### Study Outcomes

The primary outcome was neurodevelopment at 12 months post randomization. Neurodevelopment was defined as the total score of the Ages and Stages Questionnaire, Third Edition (ASQ-3).^12^ Baseline ASQ-3 scores were also collected after surgery to capture premorbid neurodevelopment.

Secondary outcomes included HRQOL, defined as the total score of the Pediatric Quality of Life Inventory (PedsQL),^13,14^ and functional status, assessed using the modified Pediatric Overall Performance Category (mPOPC).^15^ Two additional outcomes were analyzed and reported: mortality at 12 months post randomization and a composite outcome of mortality or severe neurodevelopmental impairment (≥2 ASQ-3 domains ≥2 SD below the mean). These variables were not prespecified but, instead, were constructed during analyses to assess the impact of truncation due to death. Descriptions of the study outcomes are provided in the eMethods in Supplement 2.

### Statistical Analysis

Baseline clinical and demographic characteristics at the time of surgery were summarized using mean (SD), median (IQR), and number (percentage), depending on the distribution of the variable, and presented by study group (NO or standard care). Characteristics of respondents who did and did not complete follow-up questionnaires are presented by study groups. Study participants are presented and analyzed according to their modified intention-to-treat assignment, and unless otherwise specified, analyses were undertaken using the population of participants without missing data for the outcome under investigation.

For the primary outcome, a generalized linear model was used, including the study group as a fixed effect for the unadjusted model, original stratification variables in the RCT as further fixed effects (age at randomization and lesion type), and study site as a random effect. For secondary outcome measures, generalized linear models were used for continuous outcomes, logistic regression for binary outcomes, and multinomial logistic regression when the outcome variable had more than 2 categories. Sensitivity analyses were performed for the ASQ-3 total and domain scores, including the corresponding baseline ASQ-3 total and domain score for children with a corrected age of more than 28 days at baseline.

Prespecified subgroup analyses were performed for the primary outcome for study strata (age <6 weeks vs ≥6 weeks and univentricular vs biventricular lesions). Interaction was assessed through inclusion of interaction terms between the stratification variable and randomization group in the model, with *P* < .05 considered significant. A forest plot was used to visualize the results in the overall group and subgroups.

Finally, we explored associations of factors relating to child, sociodemographic, surgical (including Risk Adjustment for Congenital Heart Surgery [RACHS] score and presence of low cardiac output syndrome),^9^ and intensive care unit (ICU) management^16,17^ with neurodevelopmental and HRQOL outcomes. Generalized linear models with ASQ-3 and PedsQL total scores as the outcomes were developed, with the factor as the independent variable and the site as a random effect incorporated.

Descriptions of our approach to handling missing data are provided in the eMethods in Supplement 2. The data analysis was performed using Stata/SE, version 17.0 (StataCorp LLC).

## Results

**Figure 1.**
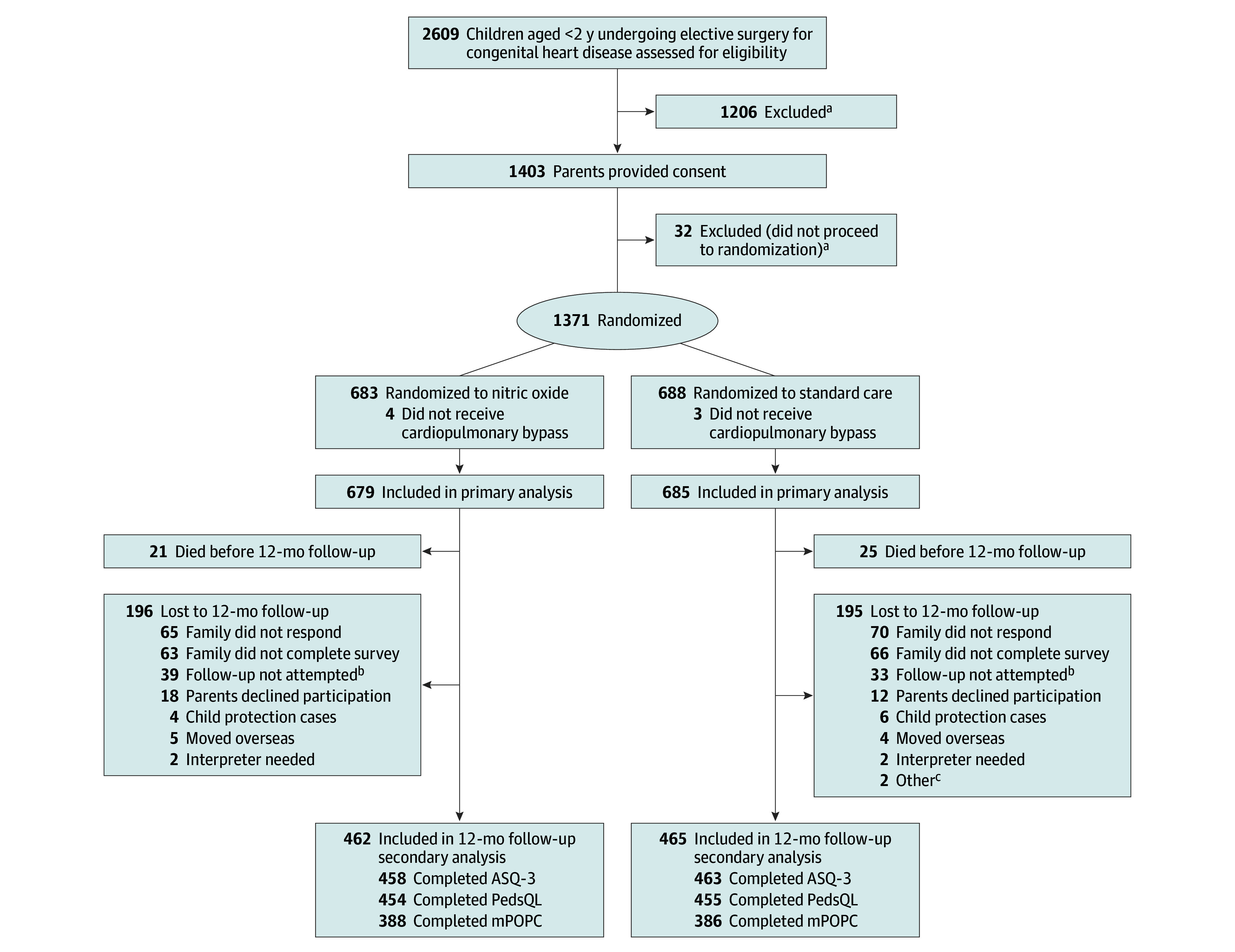
Participant Flow Diagram ASQ-3 indicates Ages and Stages Questionnaire, Third Edition; mPOPC, modified Pediatric Overall Performance Category; PedsQL, Pediatric Quality of Life Inventory. ^a^Further detail provided in Schlapbach et al.^6^ ^b^Missed due to staff unavailability. ^c^Other reasons include severe disability (1 patient) and complex social situation (1 patient).

A total of 1364 children were randomized and received CPB. Of the 1318 children (96.6%) alive at 12 months post surgery, 196 of 658 (29.8%) in the NO group and 195 of 660 (29.5%) in standard care group were lost to follow-up (Figure 1). Patients lost to follow-up were more likely to be from Indigenous populations (in both Australia and New Zealand) and to have a diagnosed congenital syndrome (eTable 1 in Supplement 2). Complete follow-up assessment data were available for 462 of 658 in the NO group and 465 of 660 in the standard care group (mean [SD] follow-up rate across sites, 66.8% [10.6%]; range by site, 54.4%-81.1%) and was performed at a median of 12.7 months (IQR, 12.1-13.9 months) after randomization. Among the 927 children in this follow-up study, the median (IQR) age at follow-up was 16.6 (13.7-19.8) months; 411 (44.3%) were female and 516 (55.7%) male; 17 (1.8%) identified as Aboriginal or Torres Strait Islander, 102 (11.0%) as Asian, 68 (7.3%) as Māori or Pacific Islander Peoples, 639 (68.9%) as White, and 101 (10.9%) as multiethnic or other race and ethnicity; 819 (88.3%) had biventricular lesions; 152 (16.4%) had a congenital syndrome; and 463 (49.9%) had a RACHS score of 2 or less. There were no clinically relevant differences between the 2 study groups for major demographic, baseline, and postsurgery characteristics (Table 1). There were no differences between the study groups in baseline characteristics previously shown to be associated with adverse neurodevelopmental outcomes, including caregiver age or level of education attained (Table 2; eTables 2 and 3 in Supplement 2).

**Table 1.** Baseline Characteristics at Time of Randomization of Children Included in the NITRIC 12-Month Follow-Up

Characteristic	No. (%)	
	Nitric oxide (n = 462)	Standard care (n = 465)
Gestational age, wk		
No. of patients	458	464
Median (IQR)	38 (37-39)	38 (37-39)
Birth weight, g		
No. of patients	447	449
Median (IQR)	3090 (2560-3465)	3075 (2500-3500)
Age at randomization, wk^a^		
Median (IQR)	12.5 (2.1-25.5)	13.8 (1.5-29.4)
<6	163 (35.3)	166 (35.7)
≥6	299 (64.7)	299 (64.3)
Weight, median (IQR), kg	4.5 (3.5-6.3)	4.8 (3.5-6.9)
Sex		
Female	193 (41.8)	218 (47.0)
Male	269 (58.2)	247 (53.0)
Race and ethnicity^b^		
Aboriginal or Torres Strait Islander	8 (1.7)	9 (1.9)
Asian	52 (11.3)	50 (10.8)
Māori or Pacific Islander Peoples	36 (7.8)	32 (6.9)
White	315 (68.2)	324 (69.7)
Multiethnic or other^c^	51 (11.0)	50 (10.8)
Congenital heart disease^d^		
Univentricular^a^	49 (10.6)	59 (12.7)
Biventricular^a^	413 (89.4)	406 (87.3)
History of previous cardiac surgery on CPB	39 (8.4)	35 (7.5)
Shunt lesions	312 (67.5)	300 (64.5)
Ventricular septal defect	183 (39.6)	185 (39.8)
Atrial septal defect	72 (15.6)	81 (17.4)
Transposition of the great arteries	70 (15.2)	69 (14.8)
Atrioventricular septal defect	43 (9.3)	37 (8.0)
Truncus arteriosus	4 (0.9)	9 (1.9)
Persistent ductus arteriosus	2 (0.4)	1 (0.2)
Other^e^	7 (1.5)	2 (0.4)
Right-sided obstructive lesions	128 (27.7)	144 (31.0)
Tetralogy of Fallot	68 (14.7)	78 (16.8)
Pulmonary stenosis or atresia	48 (10.4)	55 (11.8)
Tricuspid stenosis or atresia	9 (2.0)	8 (1.7)
Double outlet right ventricle	7 (1.5)	4 (0.9)
Other^e^	11 (2.4)	14 (3.0)
Left-sided obstructive lesions	97 (21.0)	109 (23.4)
Hypoplastic aortic arch	57 (12.3)	70 (15.1)
Hypoplastic left heart syndrome	20 (4.3)	19 (4.1)
Coarctation	3 (0.7)	2 (0.4)
Interrupted aortic arch	1 (0.2)	3 (0.7)
Other^e^	21 (4.6)	28 (6.0)
Various lesions	29 (6.3)	20 (4.3)
Total anomalous pulmonary venous drainage	19 (4.1)	11 (2.4)
Double inlet left ventricle	4 (0.9)	5 (1.1)
Other^e^	7 (1.5)	6 (1.3)
Surgical complexity		
RACHS score		
Median (IQR)	3.0 (2.0-3.0)	2.0 (2.0-3.0)
1 (Lowest risk)	14 (3.0)	16 (3.4)
2	216 (46.8)	217 (46.7)
3	127 (27.5)	122 (26.3)
4	88 (19.1)	91 (19.6)
5	1 (0.2)	3 (0.7)
6 (Highest risk)	16 (3.5)	16 (3.4)
Comorbidities		
Presurgical pediatric overall performance category		
Good, normal, or functionally normal	275 (61.1)	275 (60.3)
Mild overall disability	64 (14.2)	80 (17.5)
Moderate overall disability	90 (20.0)	87 (19.1)
Severe overall disability	21 (4.7)	14 (3.1)
Coma or vegetative state	0	0
Brain death	0	0
Congenital syndrome^d^	78 (16.9)	74 (15.9)
Trisomy 21	37 (8.0)	38 (8.2)
22q11	12 (2.6)	10 (2.2)
VACTERL	4 (0.9)	3 (0.7)
Noonan	0	4 (0.9)
Turner	2 (0.4)	0
CHARGE	1 (0.2)	0
Other^f^	23 (5.0)	19 (4.1)
Country of hospital		
Australia	345 (74.7)	348 (74.8)
New Zealand	93 (20.1)	87 (18.7)
The Netherlands	24 (5.2)	30 (6.5)
ICU outcomes		
Ventilator-free days, median (IQR), d	26.3 (24.8-27.3)	26.3 (24.2-27.2)
Duration of invasive ventilation, median (IQR), d	1.7 (0.7-3.2)	1.7 (0.8-3.8)
LCOS,^g^ need for ECLS, or death	95 (20.6)	91 (19.6)
Length of stay in ICU, median (IQR), d	3.0 (1.9-5.8)	3.1 (1.9-5.9)
Length of stay in hospital, median (IQR), d	9.8 (6.7-18.7)	9.7 (6.8-17.1)
Acute kidney injury at 48 h	91 (19.7)	78 (16.8)

Abbreviations: CHARGE, coloboma, heart defects, atresia choanae (also known as choanal atresia), growth retardation, genital abnormalities, and ear abnormalities; CPB, cardiopulmonary bypass; ECLS, extracorporeal life support; ICU, intensive care unit; LCOS, low cardiac output syndrome; NITRIC, Nitric Oxide During Cardiopulmonary Bypass to Improve Recovery in Infants With Congenital Heart Defects; RACHS, Risk Adjustment for Congenital Heart Surgery; VACTERL, vertebral defects, anal atresia, cardiac defects, tracheoesophageal fistula, renal anomalies, and limb abnormalities.

^a^ Used for stratification.

^b^ Race and ethnicity were self-reported by the parent or guardian of the child.

^c^ Ethnicities included were predominantly multiethnic children, with primary other ethnicities reported as Arabian (n = 16), Indian (n = 18), and African (n = 7).

^d^ Patients may have more than 1 type of congenital heart disease and more than 1 type of congenital syndrome.

^e^ Details of other lesions are provided in Schlapbach et al.^6^

^f^ Details of other congenital syndromes are provided in Schlapbach et al.^6^

^g^ Defined as a blood lactate level greater than 4 mmol/L with a concurrent oxygen extraction gradient of at least 35 percentage points or high inotrope and/or vasopressor requirement.

**Table 2.** Sociodemographic Characteristics of Children and Their Parents or Caregivers Who Participated in the NITRIC 12-Month Follow-Up

Characteristic	No. (%)		Difference (95% CI)
	Nitric oxide (n = 462)	Standard care (n = 465)	
Age of the enrolled child at follow-up, median (IQR), mo			
Chronologic age	16.4 (13.9 to 19.2)	16.7 (13.6 to 21.1)	−0.2 (−0.9 to 0.5)
Since randomization	12.7 (12.1 to 14.0)	12.7 (12.1 to 13.8)	0.0 (−0.2 to 0.2)
Respondent			
Mother	399 (86.4)	399 (87.8)	−0.6 (−5.0 to 3.9)
Father	49 (10.6)	51 (11.2)	0.4 (−3.6 to 4.4)
Other^a^	2 (0.4)	4 (0.9)	0.4 (−0.6 to 1.5)
Missing	12 (2.6)	11 (2.4)	NA
Respondent age, y			
<20	1 (0.2)	6 (1.3)	1.1 (0 to 2.2)
21-30	130 (28.1)	130 (28.0)	−0.2 (−6.0 to 5.6)
31-40	283 (61.3)	266 (57.2)	−4.1 (−10.4 to 2.3)
41-50	33 (7.1)	45 (9.7)	2.5 (−1.0 to 6.1)
>50	3 (0.7)	6 (1.3)	0.6 (−0.6 to 1.9)
Missing	12 (2.6)	12 (2.6)	NA
Respondent highest education			
Diploma, community college, or trade	142 (30.7)	133 (28.6)	−2.1 (−8.0 to 3.7)
Bachelor	134 (29.0)	124 (26.7)	−2.3 (−8.1 to 3.4)
High school	92 (19.9)	119 (25.6)	5.7 (0.3 to 11.1)
Postgraduate	81 (17.5)	75 (16.1)	−1.4 (−6.2 to 3.4)
Primary school	0	2 (0.4)	0.4 (−0.2 to 1.0)
Missing	13 (2.8)	12 (2.6)	NA
Respondent employment status			
Stay-at-home parent	180 (39.0)	169 (36.3)	−2.6 (−8.9 to 3.6)
Part time	154 (33.3)	160 (34.4)	1.1 (−5.0 to 7.2)
Full time	90 (19.5)	107 (23.0)	3.5 (−1.7 to 8.8)
Other^b^	25 (5.4)	16 (3.4)	−2.0 (−4.6 to 0.7)
Missing	13 (2.8)	13 (2.8)	NA
Current relationship status			
Life partner, de facto, or married	410 (88.7)	404 (86.7)	−1.9 (−6.1 to 2.3)
Single or separated	40 (8.7)	49 (10.5)	1.9 (−1.9 to 5.7)
Missing	12 (2.6)	12 (2.6)	NA
Current family structure			
2 Parents	413 (89.4)	419 (90.1)	−0.7 (−3.2 to 4.6)
1 Parent	36 (7.8)	32 (6.9)	−0.9 (−4.3 to 2.5)
Foster parent(s)	0	1 (0.2)	0.2 (−0.2 to 0.6)
Missing	13 (2.8)	13 (2.8)	NA
No. of people in house on a regular basis			
2	20 (4.3)	29 (6.2)	1.9 (−1.0 to 4.8)
3	139 (30.1)	125 (26.9)	−3.2 (−9.0 to 2.6)
4	157 (34.0)	180 (38.7)	4.7 (−1.5 to 10.9)
5	71 (15.4)	79 (17.0)	1.6 (−3.1 to 6.4)
6	33 (7.1)	18 (3.9)	−3.3 (−6.2 to −0.3)
≥7	30 (6.5)	22 (4.7)	−1.8 (−4.7 to 1.2)
Missing	12 (2.6)	12 (2.6)	NA
Annual household income, Australian $^c^			
0-50 000	93 (20.1)	91 (19.6)	−0.6 (−5.7 to 4.6)
51 000-100 000	139 (30.0)	140 (30.1)	0.0 (−5.8 to 5.9)
101 000-150 000	136 (29.4)	115 (24.7)	−4.7 (−10.4 to 1.0)
151 000-200 000	53 (11.5)	68 (14.6)	3.2 (−1.2 to 7.5)
>201 000	25 (5.4)	32 (6.9)	1.5 (−1.6 to 4.6)
Missing	16 (3.5)	19 (4.1)	NA
Languages spoken at home^b^^,^^d^			
English	398 (86.2)	395 (85.0)	−1.4 (−5.7 to 2.8)
Dutch	23 (5.0)	30 (6.5)	1.5 (−1.6 to 4.6)
Māori	10 (2.2)	8 (1.7)	−0.5 (−2.3 to 1.4)
Other	95 (20.6)	102 (22.0)	1.4 (−4.0 to 6.7)
Missing	12 (2.6)	11 (2.4)	NA

Abbreviations: NA, not applicable; NITRIC, Nitric Oxide During Cardiopulmonary Bypass to Improve Recovery in Infants With Congenital Heart Defects.

^a^ Respondents included in the other category were reported as grandparent (n = 5) and foster parent (n = 1). Additional sociodemographic data of children and their parents or caregivers are provided in eTable 2 in Supplement 2.

^b^ Details of other employment status and language spoken at home are provided in eTable 3 in Supplement 2.

^c^ A$1 is equal to US$0.61 as of January 10, 2025.

^d^ Patients may have more than 1 type of language spoken at home.

The mean (SD) ASQ-3 total score in the NO group was 196.6 (75.4) compared with 198.7 (73.8) in the standard group (adjusted mean difference [AMD], −2.24; 95% CI, −11.84 to 7.36) (Table 3). These findings were consistent after multiple imputation (AMD, −3.41; 95% CI, −12.86 to 6.05). There was no difference in the proportion of children with ASQ-3 scores below the mean per domain, with impairment in 1 domain (adjusted odds ratio [AOR], 0.09; 95% CI, −0.26 to 0.44), impairment in 2 or more domains (AOR, 0.01; 95% CI, −0.30 to 0.32), or composite death and neurodevelopmental impairment (AOR, 0.98; 95% CI, 0.74 to 1.28) (Table 3).

**Table 3.** Primary and Secondary Outcomes

Outcome	Nitric oxide (n = 462)	Standard care (n = 465)	Estimate of difference (95% CI)	
			Unadjusted	Adjusted^a^
Mortality, No.	679	685	NA	NA
Mortality at 12 mo, No. (%)^b^	21 (3.1)	25 (3.7)	0.84 (0.47 to 1.52)	0.85 (0.46 to 1.54)
Composite outcome, No.^b^	479	489	NA	NA
Death or neurodevelopmental impairment at 12 mo, No. (%)	145 (30.3)	151 (30.9)	0.97 (0.74 to 1.28)	0.98 (0.74 to 1.28)
ASQ-3, No.^c^	458	463	NA	NA
ASQ-3 total score, mean (SD)^d^	196.6 (75.4)	198.7 (73.8)	−2.10 (−11.73 to 7.53)	−2.24 (−11.84 to 7.36)
ASQ-3 domain score, mean (SD)				
Communication	36.4 (18.2)	37.5 (18.3)	−1.12 (−3.47 to 1.23)	−1.08 (−3.40 to 1.24)
Gross motor	38.2 (22.3)	38.0 (22.3)	0.13 (−2.75 to 3.00)	−0.07 (−2.92 to 2.78)
Fine motor	42.8 (16.5)	42.6 (16.3)	0.25 (−1.86 to 2.37)	0.28 (−1.82 to 2.39)
Problem solving	38.7 (17.3)	39.6 (16.6)	−0.90 (−3.09 to 1.29)	−0.87 (−3.05 to 1.32)
Personal-social	40.6 (15.7)	41.1 (15.4)	−0.46 (−2.47 to 1.54)	−0.49 (−2.49 to 1.52)
No. of ASQ-3 domains ≥2 SD below mean, No. (%)^b^				
0	246 (53.7)	252 (54.4)	1 [Reference]	1 [Reference]
1	88 (19.2)	85 (18.4)	0.06 (−0.29 to 0.40)	0.09 (−0.26 to 0.44)
≥2	124 (27.1)	126 (27.2)	0.01 (−0.30 to 0.31)	0.01 (−0.30 to 0.32)
ASQ-3 domain ≥2 SD below mean, No. (%)				
Communication	83 (18.1)	85 (18.4)	0.98 (0.70 to 1.38)	0.97 (0.69 to 1.36)
Gross motor	155 (33.8)	159 (34.3)	0.98 (0.74 to 1.28)	0.99 (0.75 to 1.31)
Fine motor	87 (19.0)	92 (19.9)	0.95 (0.68 to 1.31)	0.94 (0.68 to 1.30)
Problem solving	114 (24.9)	104 (22.5)	1.14 (0.84 to 1.55)	1.14 (0.84 to 1.54)
Personal-social	82 (17.9)	87 (18.8)	0.94 (0.68 to 1.32)	0.94 (0.67 to 1.31)
PedsQL, No.^e^	454	455	NA	NA
PedsQL score, mean (SD)^c^				
Total score	74.2 (15.3)	75.0 (14.6)	−0.71 (−2.65 to 1.23)	−0.81 (−2.73 to 1.12)
Physical score	79.3 (17.6)	79.9 (16.5)	−0.56 (−2.78 to 1.65)	−0.72 (−2.92 to 1.47)
Psychosocial	70.5 (16.7)	71.3 (15.7)	−0.82 (−2.93 to 1.29)	−0.88 (−2.98 to 1.23)
PedsQL ≥2 SD below mean, No. (%)^b^				
Total score	134 (29.5)	119 (26.2)	1.18 (0.88 to 1.58)	1.20 (0.89 to 1.61)
Physical score	120 (26.4)	122 (26.8)	0.98 (0.73 to 1.32)	1.00 (0.74 to 1.34)
Psychosocial	127 (27.4)	105 (23.1)	1.29 (0.96 to 1.75)	1.31 (0.97 to 1.77)
mPOPC, No.^b^	388	386		
Good, normal, or functionally normal, No. (%)	265 (68.3)	284 (73.6)	1 [Reference]	1 [Reference]
Mild overall disability, No. (%)	66 (17.0)	43 (11.1)	0.50 (0.08 to 0.92)	0.51 (0.09 to 0.94)
Moderate or severe overall disability, No. (%)	57 (14.7)	59 (15.3)	0.03 (−0.37 to 0.44)	0.03 (−0.38 to 0.44)
Coma or vegetative state, No. (%)	0	0	NA	NA
Brain death, No. (%)	0	0	NA	NA

Abbreviations: ASQ-3, Ages and Stages Questionnaire, Third Edition; mPOPC, modified Pediatric Overall Performance Category; NA, not applicable; PedsQL, Pediatric Quality of Life Inventory.

^a^ Adjusted for age at randomization, lesion type, and site.

^b^ Estimates reported as odds ratio (95% CI).

^c^ Estimates reported as mean difference (95% CI).

^d^ Maximum score of 300, with lower scores indicating more difficulties.

^e^ Items are reverse scored and linearly transformed to a scale of 0 to 100, with higher transformed scores indicating a better health-related quality of life.

The mean (SD) PedsQL total score in the NO group was 74.2 (15.3) compared with 75.0 (14.6) in the standard care group (AMD, −0.81; 95% CI, −2.73 to 1.12). There was no difference in the proportion of children with scores at least 2 SD below population norms (134 [29.5%] vs 119 [26.2%] for the NO and standard care groups, respectively; AOR, 1.20; 95% CI 0.89 to 1.61) (Table 3).

**Figure 2.**
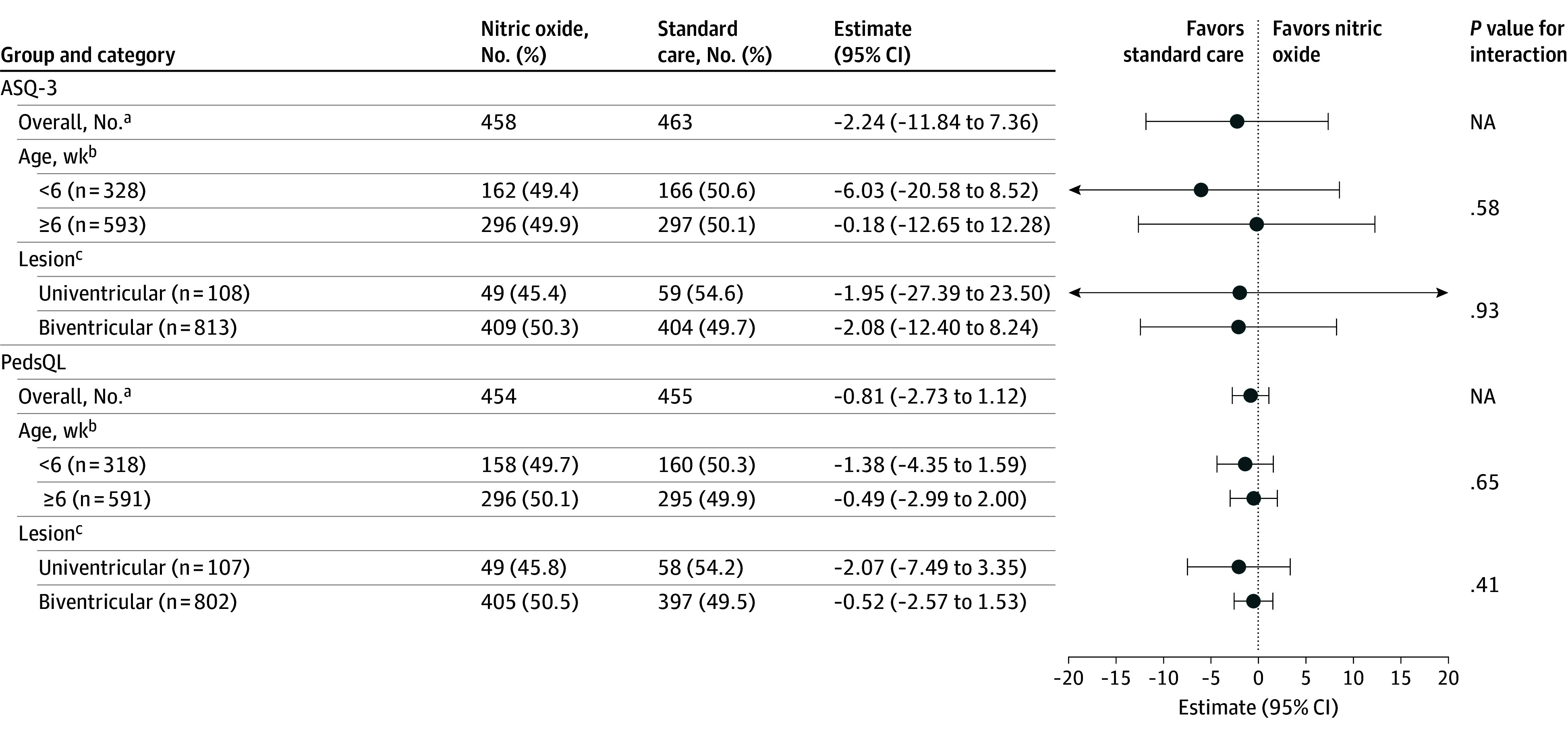
Adjusted Estimates of Difference for Total Ages and Stages, Third Edition (ASQ-3) Score and Total Pediatric Quality of Life Inventory (PedsQL) Score ^a^Adjusted for age at randomization, lesion type, and site. ^b^Adjusted for lesion type and site. ^c^Adjusted for age at randomization and site.

There was no difference in the proportion of children in the mPOPC categories of moderate to severe disability (57 [14.7%] vs 59 [15.3%] for the NO and standard care groups, respectively; AOR, 0.03; 95% CI, −0.38 to 0.44) (Table 3). Sensitivity analysis for the ASQ-3 total and domain scores, including the baseline ASQ-3 score (excluding the 310 of 927 infants [33.4%] who were <28 days corrected age at randomization), also showed no differences (eTable 4 in Supplement 2). Subgroup analyses by the study strata (age <6 weeks vs ≥6 weeks and univentricular vs biventricular lesions) did not show any differences in ASQ-3 or PedsQL scores between the NO and standard care groups (Figure 2; eTable 5 in Supplement 2).

For neurodevelopment, prematurity (gestational age <37 weeks) (AMD, −17.9; 95% CI, −28.9 to −6.9), congenital syndromes (AMD, −91.2; 95% CI, −102.8 to −79.6), univentricular lesions (AMD, −16.0; 95% CI, −29.3 to −2.6), and log-transformed length of ICU stay (AMD, −2.4; 95% CI, −3.2 to −1.6) were independently associated with worse ASQ-3 total scores (eTable 6 in Supplement 2). For HRQOL, prematurity (AMD, −5.3; 95% CI, −7.7 to −2.8), univentricular lesions (AMD, −6.2; 95% CI, −9.1 to −3.2), congenital syndromes (AMD, −9.4; 95% CI, −12.0 to −6.8), and log-transformed ICU length of stay (AMD, −0.3; 95% CI, −0.4 to −0.1]) remained independently associated with PedsQL (eTable 7 in Supplement 2).

## Discussion

In this longitudinal follow-up analysis of the NITRIC RCT in infants undergoing open heart surgery for CHD, we found that the administration of NO into the CPB oxygenator during surgery was not associated with any difference in neurodevelopmental impairment or in HRQOL at 12 months post randomization. This study is the first in our knowledge to examine the impact of extracorporeally administered NO on child neurodevelopment. Presurgical risk factors, complexity of surgery, and measures of postoperative disease severity were identified as independent variables associated with adverse neurodevelopmental outcomes at 12 months post randomization.

NO plays a key role in endothelial and platelet homeostasis, inflammation, and coagulation across multiple organ systems, and CPB has been shown to disrupt endogenous NO.^18^ Despite promising preclinical and pilot data from children and adults,^19,20,21,22^ the NITRIC study did not show any signal toward benefit or harm associated with NO on CPB in the trial or in any of many prespecified subgroup, secondary, exploratory, and sensitivity analyses. The absence of a measurable effect of the intervention on markers of postoperative illness severity (eg, organ disfunction, extracorporeal membrane oxygenation or death, acute kidney injury) and recovery (eg, ventilation-free survival) in the trial may explain why the intervention was also not associated with a benefit for long-term neurodevelopmental outcomes.

Our findings do not suggest any potential benefit of NO on cerebral protection resulting in improved long-term outcomes independent of early postoperative recovery. This finding is consistent with clinical studies on inhaled NO (iNO), which have not shown consistent benefits of NO on neurodevelopment in neonates. Specifically, a Cochrane systematic review of iNO for respiratory failure in preterm infants showed no association of iNO with a composite outcome of neurodevelopmental disability (cerebral palsy, bilateral blindness, bilateral hearing loss, or a Bayley Scales of Infant Development score >2 SD below the mean) across rescue therapy or routine use studies.^23^ Included in this review was the one study of reported benefit from routine use of iNO in neonates on developmental outcomes, showing a significant reduction in the frequency of neurodevelopment (Bayley Scales of Infant Development score >2 SD below the mean only), with a similar frequency of cerebral palsy across groups.^24^

Potential neuroprotective properties of NO have been reported in various neurologic disorders, with preclinical data showing that NO may dampen ischemic-reperfusion brain injury,^25^ preserve cerebral brain autoregulation after traumatic brain injury,^26^ prevent cerebral vasospasm after subarachnoid hemorrhage,^25^ and improve regional blood flow and decrease infarct size in ischemic stroke.^27^ In a rat model, Linardi et al^28^ found that NO 20 ppm administered in the oxygenator of selective antegrade cerebral perfusion during hypothermic circulatory arrest significantly improved neuroprotection by decreasing neuroinflammation, optimizing oxygen delivery by reducing oxidative stress and hypoxic areas, and decreasing apoptosis. As our trial did not include dose-finding studies, we cannot exclude that higher NO doses may have resulted in different outcomes, given mouse models suggesting a dose- and exposure-time dependency of exogenous NO–mediated neuroprotective effects.^29^ A double-masked RCT of iNO (80 ppm for a maximum of 72 hours) as an adjunctive therapy for severe malaria in children showed a 64% reduced relative risk in fine motor impairment at 6 months in children treated with iNO.^30^ Furthermore, the full extent of neurodevelopmental outcomes may only be evident after several years and at an age when more granular tests on executive function can be performed.^31^ With early childhood being a period of rapid neurodevelopment, failure to achieve developmental gains compared with peers may become more evident as children enter formal schooling and as social, academic, and behavioral demands increase.

Overall, the neurodevelopmental and HRQOL scores of both the NO and standard care groups were comparable to those reported in other recent cohorts of infants with CHD who have undergone open heart surgery with CPB.^32,33^ The enrolled cohort represented the contemporary spectrum of congenital heart surgery in infants, although the follow-up rate was 70.3% with a bias toward lower-risk surgeries, fewer syndromic cases, and families of higher socioeconomic background.^34^ The subanalysis of strata showed that neurodevelopment was not different between children with univentricular and biventricular lesions, and 49.9% of patients enrolled in the NITRIC trial underwent lower-risk surgeries, with a RACHS score of 2 or less. Subsequently, as the trial captured the entire spectrum of infant CPB surgeries, resulting in considerable heterogeneity, further research is needed in more homogenous populations. Kolcz et al^35^ conducted a single-center RCT of NO via CPB in children undergoing Fontan surgery and demonstrated a reduction in respiratory support time by 4.6 hours; however, they did not report a plan to conduct long-term neurodevelopmental assessments. An international multicenter RCT of NO in infants undergoing arterial switch operation, which is currently under way and will include long-term follow-up assessments,^36^ might provide more answers for this question.

The analyses of follow-up data at 12 months in 927 infants demonstrates that the large majority of children with CHD achieve reasonable to good long-term outcomes comparable to reference standards. Multivariable analyses on this prospective, highly curated follow-up database identified the impact of presurgical (syndromes, prematurity), surgical (univentricular, RACHS category), and postoperative markers of severity and recovery (low cardiac output syndrome, use of extracorporeal membrane oxygenation, duration of ventilation, duration of ICU stay) factors on both neurodevelopmental (ASQ-3) and HRQOL (PedsQL) outcomes. Importantly, several of these factors may be modifiable, paving the way toward interventions that may further reduce the adverse effects of CPB surgery on long-term outcomes. Our findings are consistent with the results of a prior large, international retrospective study that showed that when adjusting for patient, preoperative, CPB, and postoperative factors, operative factors were less important than innate patient and preoperative factors.^37^ Intensive care unit length of stay may serve as a proxy marker for severity and impact of CPB on the developing brain while also providing measures of potentially harmful exposures experienced in the ICU, such as hypotension, sedation-related toxic effects, and sleep disruption due to lights, noise, and painful procedures, which have been shown to produce considerable levels of stress in patients.^38,39,40^ A recent large, population-based study identified ICU length of stay as consistently associated with school achievement in patients treated in the pediatric ICU.^41,42^ Optimizing the postoperative ICU environment for the developing brain thus carries considerable potential for improving neurodevelopmental outcomes.^43^

### Limitations

Our study has several important limitations. First, interpretation of the findings is restricted to children younger than 2 years who had open heart surgery. Furthermore, inclusion was not restricted to the child’s first surgery, and they may have also undergone subsequent surgeries during or after the study period. Second, approximately 30% of the surviving infants were lost to follow-up. This group differed by race and ethnicity and the presence of congenital syndromes. Acknowledging this limitation, we are currently following up this cohort to school entry and are working with patients and families with lived experience of CHD to explore factors contributing to attrition.^44^ We also only collected sociodemographic information at the time of follow-up; therefore, we cannot exclude other modifiable sociodemographic factors as time-varying confounders. Third, the ASQ-3 is a parent-completed neurodevelopmental screening tool and does not provide the comprehensive assessment that an independent clinician would administer face to face. Although we adjusted our analyses for baseline neurodevelopment, we were unable to capture a baseline neurodevelopmental assessment due to age at randomization being less than 28 days corrected in 310 infants (33.4%). This limitation may have introduced important biases as surgery within the first few weeks of life is known to be more complex and carry a higher risk of neurodevelopmental impairment.^17^ While this study was representative of children with CHD more broadly, the heterogeneity in patient age and type and complexity of heart surgery limits the generalizability of the findings. Additionally, at the time of follow-up, the patients were aged 1 to 3 years, which may be too early to identify more subtle differences often seen in children with CHD, such as executive and adaptive function.^31^ This highlights the importance of conducting longer-term follow-up studies in this cohort. The findings of an ongoing follow-up study that evaluates this cohort up to the age of 5 years may show whether relevant neurodevelopmental outcomes become evident later in life.^44^

## Conclusions

In this RCT of infants undergoing open heart surgery for congenital heart defects, administration of NO via CPB did not improve neurodevelopmental or HRQOL outcomes 12 months after surgery. Further research should explore homogenous cohorts with higher RACHS scores and higher-dose or alternative therapies to improve neurodevelopment in children with CHD.

## Notes

Supplement 1.
Trial Protocol and Statistical Analysis Plan

Supplement 2.
eMethods

eReferences
**eTable 1.** Baseline Characteristics of Children Lost to Follow-Up Compared With Those Included in NITRIC 12-Month Follow-Up **eTable 2.** Additional Sociodemographic Characteristics of Children and Their Parents/Caregivers Who Participated in NITRIC 12-Month Follow-Up **eTable 3.** Listing of Sociodemographic Data Contained in ‘Other’ Categories **eTable 4.** Sensitivity Analysis for Comparison of Primary and Secondary Outcomes **eTable 5.** Adjusted Estimates of Difference for Total ASQ-3 Score and Total PedsQL Score **eTable 6.** Factors Associated With Neurodevelopmental Outcome (ASQ-3 Total Score at 12 Months) **eTable 7.** Factors Associated With Health-Related Quality of Life Outcome (PedsQL Total Score at 12 Months)

Supplement 3.
Nonauthor Collaborators

Supplement 4.
Data Sharing Statement
