# Supplementary material for: Neurodevelopmental Outcomes After Nitric Oxide During Cardiopulmonary Bypass for Open Heart Surgery: A Randomized Clinical Trial
Source: JAMA Netw Open. 2025 Feb 5;8(2):e2458040. doi: 10.1001/jamanetworkopen.2024.58040 (PMC11800016; doi:10.1001/jamanetworkopen.2024.58040)
Supplement: Supplement 1. — Trial Protocol and Statistical Analysis Plan [file jamanetwopen-e2458040-s001.pdf]

Supplementary Materials to

Neurodevelopmental Outcomes After Nitric Oxide During Cardiopulmonary Bypass for Open Heart Surgery: The NITRIC 12-month outcome study.

Debbie A Long1-3#*, PhD, Kristen S Gibbons3*, PhD, Stephen B Horton4-6, PhD, Kerry Johnson3, GradCertPaed, David HF Buckley7, MBChB, Simon Erickson8, MBBS, Marino Festa9,10, MD (Res), Yves d'Udekem5,11, MD, PhD, Nelson Alphonso12,13, MD, Renate LeMarsney3, MPH, David S Winlaw14,15, MbChB, Kate Masterson6,16, MSc, Kim van Loon17, MD, PhD, Paul J Young18-21, PhD, Andreas Schibler22,23, MD, Luregn J Schlapbach2,3,24^, MD, PhD, Warwick Butt5,6,17,21,25^, MD for the NITRIC Study Group, the Australian and New Zealand Intensive Care Society Paediatric Study Group (ANZICS PSG).

Correspondence:
Associate Professor Debbie Long
School of Nursing
Centre for Healthcare Transformation
Queensland University of Technology
Email: da.long@qut.edu.au

Study protocol.

STUDY PROTOCOL

Nitric Oxide during Cardio Pulmonary Bypass during surgery for congenital heart defects: A Randomised Controlled Trial.

Protocol Version1.5.

25th June 2020

Trial registry

ACTRN12617000821392

INVESTIGATORS:

A/Prof Andreas Schibler, Queensland Children's Hospital, South Brisbane

A/Prof Luregn Schlapbach, Queensland Children's Hospital, South Brisbane

A/Prof Warwick Butt, Royal Children's Hospital, Melbourne

A/Prof John Beca, Starship Children's Hospital Auckland

A/Prof Steve Horton, Royal Children's Hospital, Melbourne

Dr Marino Festa, Westmead Children`s Hospital, Sydney

Dr Simon Erickson, Perth Children`s Hospital, Perth

Deborah Long, PhD, Queensland Children's Hospital, South Brisbane

Revision Chronology:

**Date of change**	**Summary of changes**
**16.7.2017 (Version 1.1.)**	Westmead and Perth added as sites<br>Exclusion criteria modified<br>Pre-Planned subgroup analyses specified<br>Stratification by age: cut off modified<br>Re-enrolment defined
**20.11.2017 (Version 1.2)**	All outcomes censured at 28 days<br>Specification of contact of parents pre surgery<br>Adding Utrecht MC (NL) as study site
**12.07.2018 (Version 1.3)**	Addition of Bayley III assessment at 12 months at sites where funding and infrastructure allows<br>Addition of Socioeconomic survey at 12 months to all sites<br>Addition of hyperoxia as a pre-planned analysis, including transcriptomics<br>Revision of study sample size

**10.10.2018 (Version 1.4)**	28 day data collection specified<br>Addition of authority to access medical records from other healthcare facilities<br>Contact with families: modified to include all attempts to check child's status prior to contact<br>Inflammatory bloods: modified to optional collection
**25.6.2020 (Version 1.5)**	phone consent option during COVID-19 pandemic<br>Specified use of biobanked bloods<br>Monitoring plan specified<br>Trial stop date updated

CONFIDENTIAL

This document is confidential and the property of the investigators. No part of it may be transmitted, reproduced, published, or used without prior written authorisation from the investigators.

Statement of Compliance

This document is a protocol for a research project. This study will be conducted in compliance with all stipulation of this protocol, the conditions of the ethics committee approval, the NHMRC National Statement on ethical Conduct in Human Research (2007) and the Note for Guidance on Good Clinical Practice (CPMP/ICH-135/95).

Table of Contents

PROTOCOL SYNOPSIS 7
GLOSSARY OF ABBREVIATIONS 8
ADMINISTRATIVE INFORMATION 10
1.1. Trial registration 10
1.2. Sponsor 10
1.3. Expected duration of study 10
1.4. Author Contributions 10
2. INTRODUCTION AND BACKGROUND 13
2.1. Background and rationale 13
2.2. Hypothesis and Aim 19
3 STUDY OUTCOMES 19
3.1 Primary outcome 19
3.2 Secondary outcomes 19
3.3 Pre-planned secondary analyses: 19
4 STUDY DESIGN 20
4.1 Type of Study 20
4.2 Study Setting 21
5 PARTICIPANTS, RECRUITMENTAND CONSENT 21
5.1 Eligibility Criteria 21
5.1.1 Inclusion criteria 21
5.1.2 Exclusion criteria 21
5.2 Recruitment and identification of potential participants 22
5.3 Consent 22
6 INTERVENTION 25
6.1 Treatment arms 26
6.2 RANDOMISATION 27
7 STUDY PROCEDURE 27

7.1 Screening ... 27
7.2 Data Collection ... 27
7.3 Blood Sample Collection for Inflammation analysis ... 29
7.4 *HEALTH ECONOMIC EVALUATION* ... 31
7.5 *NEURODEVELOPMENTAL EVALUATION* ... 31
7.6 *MONITORING* ... 32
8 ADVERSE EVENTS AND RISKS ... 33
8.1 Definitions ... 33
Serious Adverse Event (SAE) is any AE that results in: ... 33
- Death ... 33
- Is life-threatening ... 33
- Requires prolongation of existing hospitalisation ... 33
- Results in persistent or significant disability/incapacity ... 33
- Requires intervention to prevent permanent impairment or damage ... 33
8.2 Assessment and documentation of adverse events ... 33
8.3 Eliciting adverse event information ... 34
8.4 Serious adverse event reporting ... 34
9 DATA MANAGEMENT ... 34
9.1 Data Collection ... 34
9.1.1 Source Data ... 34
9.1.2 Data Capture Methods ... 34
9.2 Data Storage ... 34
9.3 Sample Storage ... 34
9.4 Record Retention ... 35
10 STUDY OVERSIGHT ... 35
10.1 Quality Control and Quality Assurance ... 35
11 STATISTICAL METHOD ... 36
11.1 *ANALYSIS PLAN* ... 36
11.2 *SAMPLE SIZE* ... 36

12 ETHICS AND DISSEMINATION 36
12.1 Research Ethics Approval 36
12.2 Modifications to the protocol 36
12.3 Protocol Deviations 37
12.4 Confidentiality 37
12.5 Dissemination and translation plan 37
13 APPENDICES 41
13.1 Informed consent materials 41
13.2 Case Report Forms (CRFs) 42
13.3 Causality and Assessment of Severity – Adverse Events 42

PROTOCOL SYNOPSIS

Title	Nitric Oxide during Cardio Pulmonary Bypass during surgery for congenital heart defects: A Randomised Controlled Trial.
Objectives	To determine whether adding nitric oxide to the oxygenator during cardiopulmonary bypass results in improved post-operative morbidity as measured by a reduction post-operative length of mechanical ventilation (ventilator-free days), a reduction in Low Cardiac Output Syndrome (reduced lactate, higher central venous saturations, lower inotrope requirement), lower ECMO requirement, a lower length of hospital and ICU stay, and lower mortality.
Design	Randomised control trial
Outcomes	Post-operative morbidity
Number of Participants	1320
Population	Infants and children < 2 year undergoing open heart surgery with cardiopulmonary bypass.
Intervention	Gaseous nitric oxide vs standard care
Study Duration	4 years

Synopsis: Nitric Oxide on Cardio Pulmonary Bypass in Congenital Heart Disease

Each year, over 2000 children are born with congenital heart disease in Australia, of which the majority requires surgical intervention. Congenital heart disease ranks still within the top causes of infant mortality in industrialized countries. Despite considerable advances over the past decade, the exposure to cardiopulmonary bypass (CPB), which is needed for most surgeries, remains responsible for major side effects: The exposure of patient blood to large artificial surfaces in the CPB circuit triggers a very strong systemic inflammatory syndrome, which leads in a third of patients to low cardiac output syndrome (LCOS). LCOS is defined as a condition with a reduced oxygen delivery to end organs due the postoperative heart not being able to meet the circulatory demand. ***LCOS manifests with severe organ dysfunction such as respiratory and renal failure, and can lead to brain hypoperfusion, cardiac arrest, and death***. Survivors are at increased risk for long term neurological impairment. Patients with LCOS require increased length of respiratory support, prolonged length of stay in intensive care and hospital, resulting in significantly increased health care costs, and translating into lifelong costs due to neurological impairment. Previous attempts to reduce the detrimental inflammatory effects of CPB using immunomodulating drugs such as corticosteroids have failed to show a demonstrable benefit. Nitric oxide is an endogenous anti-inflammatory mediator, with direct actions on endothelial bed and immunologically active cells. Previous studies suggest that the delivery of gaseous nitric oxide (NO) to bypass circuits results in myocardial protection and in a reduction in bypass-induced inflammation. We have therefore performed a randomised controlled single centre pilot trial and showed that the delivery of gaseous nitric oxide (NO) to the oxygenator of the CPB circuit for children undergoing cardiac surgery for congenital heart defect resulted in a twofold reduced incidence of LCOS, and improved patient-centred outcomes including less need for extracorporeal life support post surgery, and shorter duration of mechanical ventilation, with a trend to improved mortality.

Outcome, Significance and Innovation. In order to confirm these single centre pilot data we aim to investigate in a multicentre randomised controlled trial if NO reduces length of mechanical ventilation as a primary outcome, and reduced LCOS/ECLS/death as secondary outcomes. This study is supported by a well-experienced paediatric intensive care research and cardiac surgical network across Australia and New Zealand and is strengthened by clinically very important endpoints such as mortality and utilisation of intensive care resources. The investigators have substantial track record in multi-centre trials. This study would represent the ***first international population based interventional trial in cardiac surgical and paediatric intensive care patients.***

GLOSSARY OF ABBREVIATIONS

ABBREVIATION	TERM
CPB	Cardio Pulmonary Bypass
CHD	Congenital Heart Disease
ECLS	Extracorporeal Membrane Oxygenation
NO	Nitric Oxide
PICU	Paediatric Intensive Care Unit

ADMINISTRATIVE INFORMATION

1.1. Trial registration

The trial will be registered with the Australia New Zealand Clinical Trials Registry.

1.2. Sponsor

The following peer-reviewed grants have been received by study CIs. None of the funding bodies have any involvement in study design, conduct, nor analyses.

Study Sponsor	**Children's Hospital Foundation, Brisbane (CIA Schlapbach)**<br>**Heart Kids Australia (CIA Schlapbach)**<br>**NHMRC (CIA Schibler)**<br>**Children's Hospital Foundation - Mary McConnell (CIA Long)**
Contact name	
Address	

1.3. Expected duration of study

A recruitment period of 4 years is expected to achieve the required sample size.

Start date: 1.7.2017

Expected stop date: Recruitment completion by 31.12.2020; monitoring and analyses completed by 31.6.2021. The 1-year follow-up is expected to be completed by 31.12.2021. Final analyses completed by 30.6.2022.

1.4. Author Contributions

**Name**	**Summary of contribution**
**A/Prof Andreas Schibler (CIA)**	CIA will be responsible for the set up of the trial, initial site visits and all the administration and logistics of the trial. He will also be responsible to set up the relevant DSMB, communication with ethics and governance. In addition to the overall lead, CIA will also be the local principle investigator at Queensland Children's Hospital and overseeing the recruitment of patients and adherence to study protocol in collaboration with AIs Alphonso and Zazulak.

Name	Summary of contribution
**A/Prof Luregn Schlapbach (CIB)**	In addition to supporting up CIA as study coordinator proxy, CIB will be site investigator with CIA at the Queensland Children`s Hospital site. CIB Schlapbach will be responsible to oversee trial logistics, including database setup (RedCap), eCRF design, and will be primarily responsible to design, implement, monitor, and analyse the inflammation marker data established through this project.
**A/Prof Warwick Butt (CIC)**	CIC has been providing expertise in study design, trial protocol development and trial management as he designed and led the pilot study. CIC will also be responsible to set up the relevant ethics and governance at a site level (Royal Children`s Hospital Melbourne). In addition to being one of the overall lead investigators, CIF will also be the local principle investigator at Royal Melbourne Children's Hospital, the largest centre for patient recruitment, where he will oversee the recruitment of patients and adherence to study protocol.
**A/Prof John Beca (CID)**	CID will be involved with the set-up and logistics of the trial, trial conduct and finally, analysis and presentation of the results. He will also be responsible to set up the relevant ethics and governance at a site level (Starship Children`s Hospital, NZ). In addition to being one of the overall lead investigators, CID will also be the local principle investigator at Starship Children's Hospital where he will oversee the recruitment of patients and adherence to study protocol.

Name	Summary of contribution
**A/Prof Steve Horton (CIE)**	CIE will be involved with the set-up and logistics of the trial, trial conduct and finally, analysis and presentation of the results. He will also be responsible to lead study perfusionists across the sites and will oversee adherence to study protocol. CIE has been providing expertise in study design, trial protocol development and trial management as was involved in design and conduct of the pilot study.
**Marino Festa**	Marino Festa will be responsible for recruitment at Westmead site.
**Simon Erickson**	Simon Erickson will be responsible for recruitment at Perth site.
**Deborah Long, PhD (AIA)**	AIA will contribute to study setup and logistics. In addition, Deborah Long will be responsible for secondary analyses on delirium and neurodevelopment
**Kerry Johnson**	Kerry Johnson will be the nitric study coordinator and contribute to study setup and logistics, coordinate study nurses across the study sites, and will be responsible for monitoring study sites and recruitment at Queensland Children Hospital site.

2. INTRODUCTION AND BACKGROUND

2.1. Background and rationale

Epidemiology of Congenital Heart disease – expected impact on the next decades: The incidence of congenital heart disease (CHD) is approximately 1/100 life born children, of which up to 50% at some stage during their life require cardiac surgery to correct the underlying abnormality. In the USA the costs for CHD was U$ 1.9 billion in 2011 with an average cost per patient of U$ 25,000 (Centre for Disease Control and Prevention, USA). In Australia, over 2000 children are born each year with CHD, and over 30,000 children with CHD are currently living in Australia. CHD ranks still within the top five causes of infant mortality in most industrialized countries. >75% of infants born with a critical CHD (requiring surgical intervention to survive) survive to one year of age. Over 80% of cardiac surgical procedures require cardiopulmonary bypass (CPB). While the cardiac surgical and intensive care mortality in children following cardiac surgery is low with an 2-5% peri operative death rate dependent on the complexity of the procedure, major postoperative morbidity is common and translates into an increased rate of longterm mortality, morbidity, and disability [1].

Life-threatening side effects of cardiopulmonary bypass (CPB) and myocardial ischemia during surgery - Excessive systemic inflammation leads to Low Cardiac Output Syndrome (LCOS): Despite major improvements in CPB devices, the exposure of host blood to large artificial organ surfaces combined with myocardial injury during planned myocardial ischaemia, albeit partially protected by cardioplegia, result in a significant systemic inflammatory response of the patient. Indeed, the strong CPB triggered systemic inflammatory syndrome, is responsible for the most serious and potentially life-threatening side effects after heart surgery. CPB, hypoxic-ischemic injury, and the release of damage-associated molecular patterns trigger an inflammatory cascade closely related to sepsis-induced systemic inflammatory response syndrome (SIRS) [2]. It is characterized by endotoxin release, leukocyte and complement activation, and widespread activation of inflammatory mediators, resulting in endothelial leak, increased oxygen consumption, and organ dysfunction [3]. During CPB applying cardioplegic arrest allows the surgeon to operate on a still heart. While the heart is arrested, myocardial blood flow stops, which results in ischemia and myocardial injury. With restoration of blood circulation at the release of cross-clamping, CPB provides full flows with high oxygen content which can lead to reperfusion injury. Central to the pathophysiology of reperfusion injury is a robust local inflammatory response, children appearing to be particularly susceptible to developing multisystem organ failure as a result of these processes [4, 5]. As a result of the combined effects of direct CPB-related inflammation, myocardial ischemia, and reperfusion, the newly operated on heart is unable to meet the metabolic demands of the body resulting in organ hypoperfusion. This situation is called Low Cardiac Output Syndrome (LCOS), and is commonly defined as increased need for inotropes, increased arterial-venous oxygen extraction, increase in blood lactate levels (metabolic acidosis), decrease in urine output (oliguria) and need for extracorporeal life support (ECLS)[6].***This CPB triggered systemic inflammatory response leads to acute cardiac dysfunction, with limited reserve to respond to meet the metabolic demands of the vital organs. This situation is defined as Low Cardiac Output Syndrome (LCOS).***

Children post bypass commonly develop a low cardiac output syndrome (LCOS) which may be life

threatening and represents the major determinant of postoperative outcomes. LCOS manifests with severe organ dysfunction such as respiratory and renal failure, and can lead to organ and brain hypoperfusion, cardiac arrest, and death. The severity of the LOCS is influenced as well by the type of surgery performed, pre surgical condition of the patient and strongly dependent on non-surgical injury of the heart muscle due to CPB. Several studies have shown that postoperative morbidity and mortality are strongly determined by LCOS (Table 1), which is present in ca. 25-40% of children post CPB in the hours immediately following heart surgery [7]. LCOS may lead to a transient or permanent organ damage, brain ischemia, cardiac arrest and death. ***Long-term outcome may be affected by acute injury of the developing brain caused by brain ischemia during LCOS*** [8].

CPB-related injuries are most pronounced in infants and young children for several reasons, including higher metabolic rate, stronger inflammatory response, higher bypass circuit to patient blood volume, and altered homeostasis. At the same time this is the cardiac surgical group with highest mortality, and with the highest risk of long-term neurological sequelae due to the vulnerability of the immature developing brain. In addition, bypass-induced modulation of inflammatory cytokines can lead to subsequent immunoparalysis enhancing the risk of postoperative invasive infections [9]. Should LCOS become apparent, the level of support that the newly operated on heart receives is increased using fluid boluses and inotropes. Organ replacement such as renal dialysis and prolonged mechanical ventilation may be required. In the most severe cases, the heart is supported mechanically with extracorporal life support (ECLS). Considering the severe impact of LCOS on patient centered outcomes after surgery for congenital heart disease in children, improved strategies targeting LCOS are urgently needed[8]. Interventions leading to reduced LCOS have a high likelihood to lead to a reduction in the incidence of organ failure, reduce the severe major events such as need for mechanical support of the heart with ECLS, and shorten postoperative ventilation and ICU length of stay.

Current strategies to reduce LCOS are insufficient and lack evidence for benefit:

Steroids. The most common but controversial approach to reduce LOCS is using steroids (methylprednisolone) given preoperatively to reduce the inflammatory response. There have been few prospective randomized controlled trials of corticosteroids in children undergoing cardiac surgery with conflicting results[10]. Patients randomized to dexamethasone in a relatively small study had significantly less fever, required less supplemental fluid, had greater preservation of renal function and less impairment of oxygenation, and experienced a significantly shortened duration of mechanical ventilation and length of stay in the intensive care unit. In another trial investigating the effect of methylprednisolone 4 h prior to CPB and in the bypass prime showed that these patients who received two doses of methylprednisolone had significantly less fever, required less fluid, had a significantly reduced oxygen extraction ratio and experienced a trend toward reduced length of stay in the intensive care unit ($p = 0.07$) [11]. A recent multicentre study on infants undergoing Norwood surgery reported increased mortality in patients receiving intraoperative steroids, confirming previous concerns about risks and lack of benefit of steroids [12]. The latest adult data suggests that steroids could cause more harm than benefit [13].

Modified Ultra Filtration (MUF). Another prophylactic attempt is using modified ultrafiltration which

is used in the vast majority of pediatric cardiac centers [14]. Ultrafiltration removes water, reverses haemodilution and eliminates low-molecular-weight substances, including inflammatory mediators [15]. Ultrafiltration may be used during CPB (i.e., conventional ultrafiltration, CUF) or once CPB is completed (i.e., MUF), with the composition of filtrates being identical and the assertion that a greater amount of fluid and therefore solute may be removed following CPB than can be removed with CUF alone. While some studies have shown a significant beneficial effect of MUF on the post-operative course, others have failed to do so [16].

<u>The proposed physiological effect of Nitric Oxide (NO).</u>Nitric oxide (NO) is an endogenous anti-inflammatory mediator, with direct protective actions on the endothelial bed and on immunologically active cells. NO has been shown to have a myocardial protective effect, through a reduction of reperfusion injury [17]. NO generation is essential to regulate endothelial function and microvascular inflammation, and dysregulation of endogenous NO during CPB may aggravate the subsequent inflammatory response [18]. Several animal and human studies have demonstrated that exogenous NO can reduce myocardial damage after clinical and experimental settings of ischemia and arrest [19-22]. Reduced nitric oxide (NO) signalling is associated with several known risk factors for the most common cardiovascular diseases (CVDs) [23]. Organic nitrates, such as nitroglycerin (also known as glyceryl trinitrate), have been used clinically in the treatment of CVDs for more than 150 years, but it was only in the late 1970s that their beneficial effects were shown to be due to the release of NO [24, 25].NO has since been found to be produced endogenously and to have a key role in the regulation of many physiological processes, including cardiovascular function [3]. Together, these findings have triggered substantial interest in the identification of ways to therapeutically modulate NO signalling. Only a few drug candidates designed to directly activate NO signalling have reached the clinic, the most notable of these being inhaled NO for the treatment of newborns with pulmonary arterial hypertension (PAH) [26] and phosphodiesterase (PDE) inhibitors to treat erectile dysfunction [27]. Therapeutic modulation of NO signalling is challenging, as the effect must occur at the correct location, time and dose. These pharmacokinetic characteristics have been an ***advantage in the use of inhaled NO with minimal systemic side effects*** [26]. Inhaled nitric oxide reaches the huge surface of the pulmonary vascular bed, which represents the most powerful endothelial organ in humans. Nitric oxide, when administered as an inhaled gas to adults undergoing cardiac surgery on CPB, was able to blunt the release of markers of myocardial injury and to antagonize the left ventricular subclinical dysfunction during and immediately after cardiopulmonary bypass [28].Directed delivery of NO to CPB may have therefore have the capacity to reduce the CPB-induced systemic inflammatory response more selectively, with minimal systemic side effects due to the short half life and localized delivery of the gaseous drug, resulting in mitigation of the detrimental consequences of CPB to the host. A previous small single centre U.S. study has tested this hypothesis and reported a reduction in bypass-induced inflammation using the delivery of gaseous nitric oxide (NO) to bypass circuits [29]. Children receiving gaseous nitric oxide into CPB had a significantly shortened duration of mechanical ventilation (8.4 versus 16.3 hours; $P < .05$) and intensive care unit length of stay (53.8 versus 79.4 hours; $P < .05$) compared with the placebo group. The patients had significantly lower troponin and B-type natriuretic peptide levels postoperatively. In addition, patients treated with NO had a less positive fluid balance, with significantly less diuretic usage, and higher haemoglobin levels postoperatively. This study had important limitations, principally the

small number of subjects (n = 16) and focus only a single type of cardiac lesion, tetralogy of Fallot.

Supportive Pilot data from our group:

Overview: Our group have performed a pilot study using a randomized-controlled trial design to deliver gaseous NO to CPB circuits in 198 children [30].The study was approved by HREC Melbourne (HREC 33112B, RCH Melbourne) was published with an editorial in Intensive Care Medicine in 2016, receiving international attention, including an award from the American Heart Association. This pilot study confirms the positive effects on NO reported in the U.S. trial, and demonstrated significantly reduced incidence of LCOS with improved patient outcomes, including lower need for ECLS, and trends for reduced length of stay, and shorter duration of ventilation. In view of these preliminary results from two studies with similar effect size, a large multicentre trial is required to test the generalizability of these findings to children requiring heart surgery. Some additional aspects compared to the pilot need to be considered in a new large RCT: randomisation of the patients is required per underlying circulatory physiology (cyanotic/acyanotic heart disease) and per centre and age group (0-28 days, 28days to 2 years). Additional data is needed to demonstrate physiological plausibility of the anti-inflammatory effect of NO.

Details on Pilot Data and Limitation of the Pilot Data: We have performed a single institution pilot trial at RCH Melbourne for this study to investigate feasibility and safety of the NO delivery in infants and children (0-16 years) undergoing CPB. In this pilot trial we allocated children to receive NO delivered into the oxygenator of the CBP machine versus control. NO was delivered at 20 ppm during the entire length of CPB. The primary outcome was feasibility and safety as well as a reduction of LOCS. The pilot study was performed in a single institution (Royal Children's Hospital Melbourne, HREC 33112B, RCH Melbourne), which is the largest paediatric cardiac surgical service in ANZ. The pilot data showed several important findings, which helped us to strengthen and improve the protocol for this proposal: children aged > 2years showed little benefit from the NO as an intervention and stratifying the children in 0-28 days and 29days-2 years showed a greater difference in the clinically relevant outcomes such as length of mechanical ventilation (compared to the original <6 weeks, 6 weeks to 2 years). This age group <28 days is commonly associated with very different types and high risk of cardiac surgical procedure. The pilot study showed overall a significant reduction of the incidence of LCOS. Since there is no accepted standard definition of LCOS the generalisability of the results can be disputed. Table 1 shows the re-analysed results for age group 0-2 years.

Post hoc analysis restricted to the age group < 2 years showed stratifying the children in 0-28 days and 29days-2 years demonstrated a greater difference in the clinically relevant outcomes such as length of mechanical ventilation compared to the original <6 weeks, 6 weeks to 2 years) **(Table 1)**. In addition, this age group <28 days is commonly associated with high risk cardiac surgical procedures.

Outcome		n	control	NO on bypass
			Mean (CI)	*Mean (CI)*
**PICU**	all	132	187.4 (120.7-254.2)	133.6 (87.4-179.8)
**Length of stay**	<28days	63	171.7 (119.4-224.0)	161.3 (80.9-241.8)
**(hrs)**	29d-2yrs	69	199.2 (86.4-312.1)	107.5 (56.3-158.6)
**Length of**	all	130	97.8 (61.5-134.1)	65.1 (45.5-74.6)
**Mechanical**	<28days	61	107.1 (69.2-144.9)	71.3 (47.2-95.5)
**Ventilation (hrs)**	29d-2yrs	69	90.3 (30.6-150)	59 (27.2-90.8)
**Low cardiac**	all	132	35.8% (24.5-48.5%)	12.3% (5.5-22.8%)
**output syndrome**	<28days	63	48.4% (30.2-66.9%)	15.6% (5.2-32.8%)
**(LCOS)**	29d-2yrs	69	25% (10.1-39.9%)	9.1% (1.3-19.4%)

Table 1. Pilot trial outcome in 132 children < 2 years of age undergoing CPB; LCOS: Low Cardiac Output Syndrome. We do not provide significance thresholds as the pilot study was not powered for the proposed stratification.

Since there is no accepted standard definition of LCOS the generalisability of the results can be disputed and a larger clinical trial is needed to answer whether NO during CPB leads to a patient centred relevant outcome. The pilot trial also did not link and explain the plausibility of the intervention to LCOS as no direct markers of the inflammatory process post CPB were measured.

Why is this study important? Postoperative paediatric cardiac surgical patients have a high consumption of intensive care resources and are at very high risk of major complications, including cardiac arrest, death, and long-term neurological impairment. LCOS is the major determinant of poor patient outcomes, translating into prolonged PICU and hospital length of stay, prolonged need for ventilation, higher risk of organ failure, brain damage, and renal replacement. LCOS is strongly correlated with the risk ECLS, cardiac arrest, and death postoperatively. Approximately 10% of children with CHD survive with major neurological sequelae postoperatively, resulting in a massive lifelong burden for patients, families, healthcare systems, and the society [8]. An attempt to reduce

LCOS and hence the perioperative morbidity has the potential to translate not only in a reduction in intensive care resource utilisation but also to impact positively on long-term outcome.

Why should it be done multi-centre? With a multi-centre trial we can reduce the bias effect of site-specific surgical approach and CPB methods during surgery inherent in a single centre study and to gain a true understanding on the efficacy and applicability of this therapy in real-world conditions. Inherent differences that may impact on patient outcomes include the heart defect specific surgical approach, operator experience and expertise and the use of other techniques such as priming the bypass, management on bypass and modified ultra-hemofiltration post bypass. The post-operative intensive care management strategies also vary between intensive care units. This includes respiratory strategies, fluid and inotrope management as well as threshold to offer extracorporeal life support. Only an appropriately designed multi-centre study would have sufficient certainty of demonstrating a generalisable nitric oxide specific impact on outcome. We intend not to restrict the study intervention to a particular heart defect to achieve a greater generalisability of the results to any cardiac surgical procedure on CBP.

Feasibility. With our pilot study we have demonstrated that the proposed protocol applied in a complex clinical environment such as cardiac surgical service and intensive care is feasible. In our pilot trial the perfusionist was the only none blinded person for the intervention. The concentration of the NO needs to be monitored not to exceed the target concentration of 20 ppm, for which we have provided proof of technical feasibility. Surgeons, anaesthetists and ICU staff were blinded for the gas delivered. All clinically relevant data is recorded as per standard practice and can be accessed through registry data and unit internal data collection.

Expected enrolment opportunity. In 2013 The Australian New Zealand PICU Registry (ANZPICS) reported 1547 children who underwent surgery for a congenital heart defect, of which approximately 80% on CPB. On average 400-600 of children undergoing surgery for a congenital heart defect each year are <2 years of age and fulfilling the inclusion criteria in the participating hospitals. The consent rate of eligible patients was 78% in the pilot trial. With an expected conservative estimate 50-60% enrolment rate of eligible patients we expect a 3 ½ year recruitment in the study.

Research Team Performance. This trial is a multi-centre trial in the major tertiary paediatric hospitals in Australia and New Zealand with the support of the Paediatric Study Group (PSG) as the paediatric subgroup to the ANZICS Clinical Trial Group with a proven NHMRC track record. This research network has demonstrated the capacity to perform complex multicentre randomised controlled trials such as trials investigating the prophylactic hypothermia in head injury [31] or trials investigating relevant clinical outcomes in sepsis or acute lung injury [32, 33]. The group is led by several CIs holding NHMRC grants over the last years. CIA and CIB as members of the Paediatric Critical Care Research group (PCCRG) will lead this trial with extensive experience running large NHMRC funded multi-centre trials.

2.2. Hypothesis and Aim

Aims. 1. To investigate in a double blind randomized controlled trial in children undergoing open heart surgery if NO exposure during CPB reduces the postoperative duration of invasive mechanical ventilation (defined as ventilator free days within 28 days post randomisation) compared to control.

2. To demonstrate that NO reduces the incidence of low cardiac output syndrome (LCOS), requirement for extracorporeal life support (ECLS), and 28-day mortality
2. To demonstrate that NO reduces the length of PICU stay and health care costs

3. To demonstrate that NO reduces the inflammatory response following CPB.

Hypotheses. Infants and children undergoing cardiac surgery on CPB exposed to gaseous nitric oxide (NO) have compared to controls, less myocardial injury and a reduced inflammatory response, leading to reduced incidence of LCOS. We hypothesize that lower LCOS will translate into reduced secondary organ failure and a reduced postoperative intensive care resource utilisation measured by length of mechanical ventilation, and will ultimately result in a reduction in mortality and health care costs.

3 STUDY OUTCOMES

3.1 Primary outcome

Length of mechanical ventilation as defined as the duration of respiratory support for all episodes with an endotracheal tube in situ for the first 28 days post randomisation. The outcome will be reported using ventilator free days (VFD). A systematically zero value will be assigned for patients who die to allow important weight to death as the most pejorative outcome.

3.2 SECONDARY OUTCOMES

- Incidence of LCOS, need for ECLS, and Mortality (AIM 2)
- The length of stay in PICU, hospital length of stay and health care costs (AIM 3)
- Levels of systemic inflammatory markers and levels of markers of myocardial injury (AIM 4)

3.3 Pre-planned secondary analyses:

- Risk adjustment for preoperative surgical risk (RACHS and Aristotle)
- Health care costs
- Subgroup analysis for age (<28d, <42d, <90d), and univentricular or biventricular hearts
- Number and duration of postoperative organ dysfunction (assessed by respiratory, cardiovascular, renal support)

- Postoperative renal failure, maximum creatinine, need and duration of renal replacement (CVVHF, CVVHDF, peritoneal dialysis, SCUF if on ECLS postoperatively), and blood, serum and urine markers of renal dysfunction including secretory/concentration capacity
- Volume and type of fluid and of transfusions given during CPB, and during the first 24 and 48 hours post CPB in PICU.
- To compare treatment response between patients depending on pre-intervention severity assessed by markers of inflammation and organ failure
- To biochemically define responders to the intervention using markers of inflammation
- Impact of open chest
- To investigate predictors of worse outcome including transcriptomic analyses, biomarkers, and clinical factors
- To investigate the association of hyperoxia with outcomes
- Subgroup analyses on infants and children undergoing several CPB procedures during first years of life, including neurocognitive and functional outcome
- Neurodevelopmental and functional follow-up
- Incidence and severity of postoperative delirium (using sedation and delirium scoring)
- Relationship of postoperative delirium and neurodevelopmental outcomes to intervention and inflammation

4 STUDY DESIGN

4.1 Type of Study

A multi-centre randomised controlled and blinded study in children < 2 years of age undergoing open heart surgery on cardiopulmonary bypass.

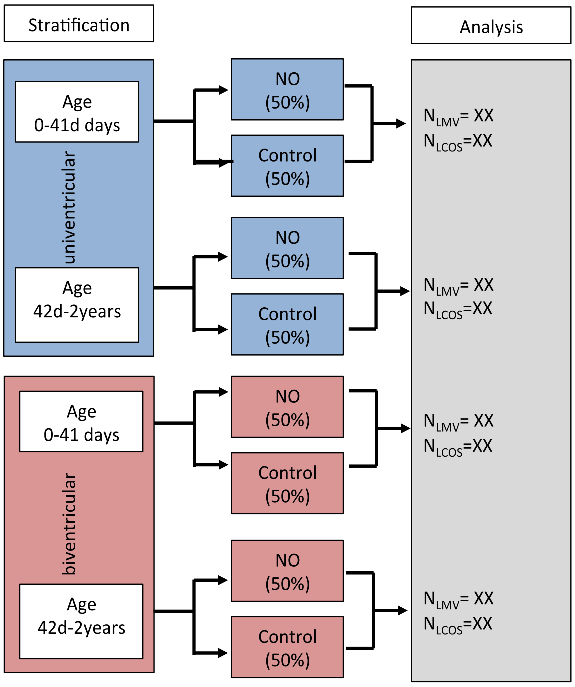

Figure 1. Study Flow Diagram:

4.2 Study Setting

Cardiac and Paediatric Intensive Care Services of Royal Children's Hospital, Melbourne; Starship Children's Hospital Auckland NZ; Queensland Children's Hospital Brisbane; Westmead Children`s Hospital, Sydney; Princess Margaret Children`s Hospital, Perth; and Utrecht Medical Center, Utrecht, Netherlands.

5 PARTICIPANTS, RECRUITMENTAND CONSENT

5.1 Eligibility Criteria

5.1.1 Inclusion criteria

- All infants and children < 2 years of age undergoing open heart surgery on CPB
- Elective cardiac surgery and consent of parents/guardian.

5.1.2 Exclusion criteria

- Signs of persistently elevated pulmonary vascular resistance preoperatively requiring iNO or preoperative intravenous use of drugs involved in the NO pathway such as GTN, within 48 hours prior to CPB (oral sildenafil treatment alone is not an exclusion).
- Patient is on ECLS immediately prior to surgery

- Chronic ventilator dependency
- Concurrent known confirmed bacterial sepsis/septic shock, diagnosed within <48hours prior to surgery and being actively treated with antibiotics at time of surgery (suspected sepsis treated with antibiotics is not an exclusion criteria unless inotropes are required for treatment of septic shock at time of surgery)
- Preoperative acute respiratory distress syndrome requiring HFOV ventilation <48 hours of surgery
- Patient requires high doses of vasoactive drugs prior to surgery with an inotrope score ≥15 met within 24 hours prior to surgery: Inotrope requirement will be calculated by means of the Vasoactive-Inotrope Score (VIS) (2): VIS = dopamine dose (mcg/kg/min) + dobutamine dose (mcg/kg/min) + 100 x adrenaline dose (mcg/kg/min) + 100 x noradrenaline dose (mcg/kg/min) + 10 x milrinone dose (mcg/kg/min) + 10,000 x vasopressin dose (U/kg/min).
- Cardiac arrest within one week (7d) prior to surgery
- Emergency cardiac surgery which may preclude obtaining informed consent (defined as acutely required life-saving procedure in a patient unlikely to survive the next hours without the surgery)
- Pre-existing methaemoglobinemia (MetHb>3%)

NB. Patients that were previously enrolled and randomised into the study with surgical procedure performed that required use of cardio-pulmonary bypass will not get re-randomised. Previously enrolled and randomised patients will undergo the same treatment allocation for subsequent surgeries, unless parents opt out. Rationale: For patients undergoing same procedures, any potentially additive protective impact of NO during CPB on long-term neurodevelopment may become diluted by use of CPB without NO during subsequent procedures. We expect that ca. 10% of all CPB cases <2 years may require subsequent CPB <2 years of age.

5.2 Recruitment and identification of potential participants

Only parents/guardians of elective cardiac surgical children will be approached for consent. Consent will be obtained from parents/guardian prior to surgery either in pre admission clinic or whilst the patient is in hospital prior to surgery. A screening log will be maintained to document missing patients.

5.3 Consent

Consent will be sought from the parents of every child <2 years of age undergoing CPB for elective cardiac surgery over the study period. When the family is seen by the surgeons in pre-assessment clinic (usually days prior to surgery), the study will be mentioned to them by the surgeon. In addition, the study team will provide study information prior to hospitalization to families, including printed study flyers, and links to online study documentation (media release). A video detailing the study has also been prepared and is available to families. These will not replace consent, but support the consent process inasmuch as parents will have more time and more information available to

consider prior to consenting. Pre-admission contact can be made by phone, too, if not feasible directly. While parents may provide verbal consent, written consent will have to be obtained no later than the day of surgery in such circumstances.

The study team will meet the family and they will be given the study Information Statement and the consent form if they are interested. When the patient and family return to hospital for the operation, further explanation will be offered by a member of the research team and consent taken if the family wish to take part in the study. All endeavours will be made to consent parents by face-to-face meetings prior to the day of surgery, however due to the changing nature of that cardiac surgical bookings parents may be approached on the morning of surgery. Early phone contact with parents not present at the hospital or not attending pre-surgical clinics will be made. If parents give consent by phone it will be later confirmed by written consent during hospital/PICU stay.

The consent form will make it clear that standard medical treatment of children will be unaffected by participation in the study. Taking part is voluntary and will be free from coercion.
The research team will be available to field any questions from the parents prior to the consent form being signed.

The following basic principles of obtaining consent will be followed: Explain the project to the child's family in language that they understand. Generally, consent should be obtained in privacy and parents given as much time as possible to consider the study before consent.

It is important that the investigator considers the parent/guardian(s) has a good understanding of what the study means for their child before they sign the consent form. If the parent/guardian(s) is unable to give informed consent, their child should not be recruited into the study. It should also be made clear that the parent/guardian(s) is free to not have their child participate in the study and that if they do so, their medical care within the hospital will not be affected in any way. In addition, the parent/guardian(s) of the child should be informed that the data collected about themselves and their child will be de-identified before publication and their child's name will not appear on any publications that may arise from the trial.

Patients that were previously enrolled and randomised into the study with surgical procedure performed that required use of cardio-pulmonary bypass will undergo the same treatment allocation for subsequent surgeries requiring bypass, unless parents opt out.

In the event of early phone contact with parents, the following script is recommended:

1. An initial phone call is made to provide information on the Nitric Oxide (NO) study to parents, this will be followed up by an email or letter with written information and will be discussed with parents in the pre-surgical admission clinic or prior to hospital admission.
2. This call is conducted over the telephone with a research team member and the child's parent or legal guardian.
3. Check the name of the parent/ legal guardian on the hospital EMR system.
4. Introduce yourself by saying the following:

"Hello my name is [your name] and I work with the doctors in the intensive care unit at the [Hospital name] as [role of research staff calling]. Are you the parent and/or legal guardian for [Child's name]?"

If they are not the legal guardian, confirm who is the legal guardian), and only proceed with the discussion with the legal guardian.

"(Child's name) is due to attend our cardiac clinic [state: day/month] to discuss his/her heart operation."

For some children it will be a hospital admission so discuss this instead of the clinic appointment.

"At the clinic/admission we would like to talk to you about a study that is happening in the hospital to see the effects of adding Nitric Oxide into the Bypass circuit. We are asking all parents of children aged 2 years or under who are going to have heart operation with bypass. Nitric Oxide will be administered along with oxygen to the bypass circuit compared to just oxygen administered, which is what we do now. The results of a small study we did in 2013 indicated that Nitric Oxide can reduce ventilation and PICU admission time, and we believe that administering it to the bypass circuit may result in less problems after surgery. We use Nitric Oxide in PICUs worldwide on small babies so it is a safe gas. During this small 2013 study there were no problems in children who received the nitric gas."

"This call is to give you some information before we meet you and give you time to think about the study. I can send you some extra information by email or post" (obtain email if they want emailed info). "*One of the research team will meet you in the cardiac pre-admissions clinic (or hospital) to talk about it but if you have any more questions you can call the research team on [provide number]"*

"If you have any concerns about who I am you may call the intensive care unit on [phone number] and ask them to confirm my identity and role in the PICU."

If anyone becomes extremely anxious about your telephone call or the questions you ask, please provide them with the hospital switch board number [phone number]. They can ask to speak to [Name], (Director of Paediatric Intensive care Unit) who can assist in clarifying any concerns that they may have. The research team will document the phone call in the notes in EMR and will organise to meet the family in clinic or hospital. An email address will be obtained if parents wish to receive additional information by email prior to clinic. Parents who refuse any further contact will be documented in the medical record at the time of phone contact so they will not be approached in clinic.

Phone consent during the COVID-19 pandemic

COVID-19 restrictions within healthcare facilities affected the recruitment process of study patients, approval was obtained to contact families via telephone when we are not able to contact as per normal routine. The patient cohort are not affected directly by COVID-19, as they are pre surgical patients and at little risk of being COVID-19 positive.

COVID-19 restriction on research/parental experience:

- Hospital health checks on arrival (temp, wellness)
- Parental/ Guardian support reduced with visitor restrictions
- Research staff restricted from high risk areas
- Research staff working from home

Verbal consent allows the study nurses to contact families who are interested in participating in the study after speaking to the cardiac surgeons. This allows families to consider research in the comfort of their own homes with no pressure of the admission and COVID-19 restrictions within the hospital. If they wish to participant, the research nurse will take verbal consent over the phone. Research will always aim to retrieve consent in the normal manner whenever possible. We would resume standard practise as soon as outpatient clinic returns to normal.

Withdrawal of Participants

The investigator may withdraw a patient from the study treatment and follow-up procedures if the patient:

- Is in violation of the protocol;
- Experiences a serious or intolerable adverse event
- Develops, during the course of the study, symptoms or conditions listed in the exclusion criteria
- Requires early discontinuation for any reason

The investigator will also withdraw all participants from the study treatment if the study is terminated. Patients are free to withdraw from the study at any time upon their request or the request of their legally acceptable representative. Withdrawing from the study will not affect their access to standard treatment or their relationship with the hospital and affiliated health care professionals

When a participant is withdrawn from the treatment and/or the study by the investigator, the reasons for withdrawal shall be recorded and captured in the CRF by the site research assistant. Where the participant withdraws from the treatment and/or the study themselves or is withdrawn by their parent/guardian, the site research assistant shall attempt to ascertain the reason(s) for withdrawal from the parent/legal guardian or competent participant and, where provided, record this data. Any results obtained prior to withdrawal from the treatment and/or the study will be included in the analysis, unless the participant or their parent/guardian withdraws consent to use any of the participant’s data.

General contact with families

Prior to any contact being made with families, particularly following their PICU admission, all attempts will be made to determine the child’s status via accessing hospital systems currently in place.

6 INTERVENTION

6.1 Treatment arms

Patients allocated to the study gas arm will receive NO, which will be blended into the fresh gas flow kept at 3L/min for the CPB oxygenator with NO levels maintained at 20 ppm via an Ikaria INOmax DSIR (Ikaria, NJ, USA) or similar device. Continuous sampling of NO and NO2 concentration will be undertaken from an access port just prior to the oxygenator. NO will be started immediately when the patient is on CPB and ceased once coming off CPB at the completion of MUFing. Patients allocated to the Placebo arm will receive standard respiratory gases (oxygen-air mix) during bypass at a flow rate of 3L/min. Partial pressures of CO2 will be maintained constantly in both study arms as per the institutional practice.

Note: If patients require several CPB runs during the same procedure, the treatment has to be provided for each run using the same treatment allocation for every run (i.e. patients allocated to study gas arm will receive NO at 20ppm for every run).

Subsequent CPB procedures: Patients that were previously enrolled and randomised into the study with surgical procedure performed that required use of cardio-pulmonary bypass will not get re-randomised. Previously enrolled and randomised patients will undergo the same treatment allocation for subsequent surgeries, unless parents opt out. The study perfusionists at each study site have access to the treatment allocation, which will remain blinded for all other study team members.

Blinding arrangements. Of the investigating team, only the study perfusionist will be aware of the randomisation and NO delivery. Blinding arrangements in the operating theatre will be achieved by covering the NO delivery system with drapes. The dedicated study NO delivery system will be connected to the CPB oxygenator at all times, independent of randomisation. The surgeons, anaesthetists and ICU staff will be not aware which treatment arm a patient been allocated to. The perfusionists will be advised that all aspects of CPB except for provision of NO (or not) should be performed in a standard fashion for study participants. The perfusionist does not attend the surgical handover in PICU. The Data Safety Monitoring Board will be unblinded in the event of a severe adverse event. In our pilot study and the previous published study by Checcia, methaemoglobin values using NO at 20 ppm were similar in both the control and intervention groups (1.4%) [29].

Perioperative Care. Techniques of anaesthesia, surgical technique or method of CPB perfusion will not be specified apart from some details of perfusion. *Rationale:* To allow site specific individual practice and not to interfere with centre specific cardiac surgical load and outcome.

All patients will receive a minimum sweep gas flow of 3.0 l/min and CO_2will be added to maintain $PaCO_2$ 35–40 mmHg to ensure NO mixing and delivery in the intervention group. Sweep gas will be set as per standard CPB practice.

Note: Patients undergoing CPB that are considered by treating physicians (surgeons, anaesthetists, or intensivists) to require inhaled NO can receive iNO at any time during or after CPB, independent of treatment allocation, delivered as an inhalational gases at the doses defined by the clinicians (usually 0 to 20ppm).

Postoperative Care. The postoperative care will be performed as per site specific standard protocol/care for all patients without any specific definition of any procedures or interventions.

Inotrope delivery, fluid management, renal replacement therapy, iNO, or indication for ECLS will be provided as per standard care locally. *Rationale*: Site specific intensive care practice shall continue for the trial.

6.2 RANDOMISATION

Study patients will be randomly allocated to NO or standard care, and stratified by age group (<42 days, 42 days -24 months) and uni-versus biventricular lesions for each site. Randomisation will done by using REDCap randomisation. For incomplete data or withdrawal of consent the intention to treat principle will be applied.

Stratification

- age group (0 to <42 days and 42 days to 2 years)
- uni-versus biventricular
- study site

Rationale for stratification: The younger age group, including neonates (<28days) and young infants up to 6 weeks of age (<42d) represents the highest surgical risk for many procedures. The pilot study demonstrated an increased effect size in the <6 week age group. Cardiac physiology (uni- versus biventricular) is a major determinant of surgical complexity, risk, and outcome. Less than10% of CPB procedures are performed in infants 28d to <42d, however the proportion of high-risk procedures in this age group is higher compared to older children. For this reason, we will stratify to < 6 weeks and > 6 weeks of age.

7 STUDY PROCEDURE

7.1 Screening

All patients undergoing either elective or emergency cardiac surgery will be screened for eligibility criteria. This will be achieved by the on call cardiac surgical team, perfusionist, and PICU study team members.

7.2 Data Collection

Baseline variables, primary end points, secondary end points, pre-determined physiological variables of interest, and process of care measures will be prospectively recorded into a study REDCap online database.

Time Points for Data Collection. All physiological and blood parameters are captured for the first 48 hours post operatively and analysed (for patients discharged earlier only data up to discharge will be collected). The specific time points for these parameters will be: on admission to PICU (within the 1st hour), 6 hours, 12, 24, 36 and 48 hours post admission (or until PICU discharge). Gross functional performance assessment will be recorded on admission to PICU and upon discharge from PICU and discharge from hospital. Follow-up to define neurological/functional outcome and quality of life (including phone interviews with parents/caregivers) will be performed at 6-12 months postoperatively.

Measured Parameters.

Baseline variables: Sex, age, weight, primary cardiac diagnosis, secondary diagnoses including comorbidities (prematurity, syndrome), pre-operative disease severity (pre-operative PICU admission and length of PICU admission, ventilation and length of preoperative invasive ventilation, inotrope use and dose, prostaglandin use pre-op, diuretics, afterload reduction, infection pre-bypass). Functional assessment at baseline pre-surgery using parental interview/questionnaire to define POPC. Pre-operative medications: steroids, antibiotics, inotropes, prostaglandin, afterload reducing agents.

Surgical intervention and bypass: Data on performed cardiac surgical procedure, RACHS and Aristotle score (scores describing the complexity of the surgical procedure), amount and type of fluids and blood products given during surgery, length of CPB (for every run if several runs during one surgery are required), aortic cross clamp time, deep hypothermic arrest (temperature, duration), and length of NO or control delivery. Steroids given during theatre. Modified ultrafiltration. MetHb, NO.

Postoperative status: Data on postoperative cardiovascular status, respiratory status, urine output will be collected. Arterial and central venous (taken from jugular, subclavian, or femoral central venous catheter) blood gas analysis including lactate measurements. Laboratory tests to define organ dysfunction platelets, white cell count, haemoglobin, creatinine, base excess, pH, pCO2, pO2, SataO2, and coagulation. Amount and type of blood products given during first 24 and 48 hours. Amount of fluid given during first 24 and 48 hours. Delayed chest closure and time to chest closure. Arrhythmia (JET, etc). Intravenous steroids given during 48 hours postoperatively. Use of iNO. MetHb. Delirium. Total inotrope hours, post-operative infections, re-intubations, cardiopulmonary resuscitation, necrotising enterocolitis, readmissions to PICU and child status will be collected at 28 days. In the event that the child has been transferred to another healthcare facility, parents are asked to consider permission to access medical records within the consenting process.

Definitions

- ***Length of mechanical ventilation*** is defined as the duration of invasive respiratory support for all episodes with an endotracheal tube in situ for the first 28 days post randomisation. The outcome will be reported using ventilator free days (VFD). A systematically zero value will be assigned for patients who die to allow important weight to death as the most pejorative outcome.
    - For infants with tracheostomies, ventilation support will be considered completed at the time point when the patient reaches pre-intervention baseline respiratory support levels
- ***LCOS*** is defined as the inability of the myocardium to provide adequate oxygen delivery (DO_2) to the tissue. DO_2 measurements are not feasible in daily practice, hence accepted surrogate measures are commonly used. For the purpose of this study, ***LCOS*** will be defined as[6]:

- blood lactate level greater than 4 mmol/l with an oxygen extraction of greater than 35% (SaO_2-SvO_2 gradient >35%); within the first 48 hours postoperatively
and/or
- high inotrope requirement: Inotrope requirement will be calculated by means of the Vasoactive-Inotrope Score (VIS) (2): VIS = dopamine dose (mcg/kg/min) + dobutamine dose (mcg/kg/min) + 100 x adrenaline dose (mcg/kg/min) + 100 x noradrenaline dose (mcg/kg/min) + 10 x milrinone dose (mcg/kg/min) + 10,000 x vasopressin dose (U/kg/hr). A score ≥15 indicating low cardiac *output syndrome.*

- Initiation of **ECLS** in theatre or postoperatively in intensive care with presence of LCOS
-
- ***Death*** within 28 days of the operation
- Length of stay in intensive care
- Hospital length of stay
- Renal failure
- Inhaled NO
- Sedation and delirium scores post surgery
- Functional performance at PICU discharge, at hospital discharge, and during follow-up (in comparison to baseline functional status, if available)
- 1-year follow-up surveys (QoL, Ages and Stages Questionnaire, socioeconomic status)
- 1-year follow-up neurodevelopmental assessment with neuropsychologist (at sites where the funding and infrastructure allows): using Bayley-III, including cognitive, language, motor, social-emotional and adaptive behaviours

7.3 Blood Sample Collection for Inflammation analysis

Biomarker Analysis and Biobanking: Blood markers for myocardial injury and inflammatory response will be collected on induction of anaesthetics (baseline – pre bypass), at admission to PICU (0 hours – post bypass) and at 12, and 24 hours for the following purposes: i) to test the impact of the intervention on markers of inflammation; ii) to stratify patients in to high- versus low inflammatory groups; iii) to biochemically define responders to the intervention (to identify patient subgroups that are more likely to respond to a specific treatment), iv) to develop improved markers of outcome post cardiac surgery. Collection of blood for biomarker analysis and biobanking is optional and parents will be asked to provide their permission during the consenting process.
Blood sampling and processing. *Preoperative baseline* (before onset of cardiac surgery, done by anaesthetist during the induction of the patient once the arterial line has been inserted): 1-2ml of EDTA blood (for DNA), 2.5ml of blood (PAXgene tube for gene expression markers), 1-2ml of serum
Postoperative at 0,12,24 hrs (in PICU until discharge to the ward): 2.5ml of blood (PAXgene tube - time point 0 only), 1-2ml of serum. The total amount of blood taken is 10-15ml for the entire study duration, which is much less than the amount of blood taken for routine perioperative care tests. The samples will be processed within 30 minutes, centrifuged, aliquotted and immediately frozen. The samples will be kept in a security protected freezer in the each institution, and will be transferred in batch every 12 months to the Centre for Children's Health Research, Brisbane, Australia. Urinary electrolytes and NH_4 will be measured from patient urine collected through the

standard IDC at baseline, admission to PICU, and 6-24hours to assess for renal concentration and secretory function. Paired arterial and venous gases will be collected postoperatively at 0,6,12,24,48 hrs (in PICU until discharge to the ward or removal of arterial line, whichever occurs first) to assess for lactate and SaO2-SvO2 gradient. Each arterial or venous blood gas sample requires 0.2mls of blood and would total 2mls across all samples. Routine paired gases are often part of standard clinical care.

Use of biobanked blood:

The following list the specific analyses to be performed using the biobanked blood samples:

Cytokine and Metabolomic Measurement and analyses*[34-36]:* Inflammatory cytokines will be measured using multiplex cytokine assay using 50uL of serum on Cytokine Human Ultrasensitive 27-Plex Panel for Luminex® Platform at Diamantina Institute, University of Queensland or similar to higher performant test dependent on availability and prize at completion of the study). For optimal costing batch effect, cytokine measurements will be done at the end of the study in all samples concomitantly. Several candidates have been selected (Levels of systemic inflammatory markers (TNF, Csf2, IL5, IL10, IFNG, IL8, IL4, IL2, IL6, IL1B). Untargeted metabolomics will be performed on plasma samples using liquid chromatography-mass spectrometry with Australian partners such as the Centre for Integrative Metabolomics & Computational Biology (Edith Cowan University). We will identify cytokine and plasma metabolites that discriminates between children with worse outcomes including LCOS and those who do not have these outcomes by assessing the latent multivariate structure of the data using Principal Components Analysis and similar tools. Candidate biomarkers identified in this analysis will then inform the development of a targeted cytokine/metabolite panel.

Markers of organ dysfunction: Markers of cardiovascular (natriuretic peptides, BNP), renal (serum neutrophil gelatinase-associated lipocalin NGAL Nephrocheck (cell cycle arrest biomarkers), IL-18, LFABP, CHEM1, FGF23), and pulmonary damage (soluble receptor for advanced glycation end products (sRAGE)) marker of myocardial injury (Troponin I) where available. Serum samples may be shipped overseas for sample measurement.

Gene and gene-expression based markers: gene changes and gene-expression will be investigated, to test the individual response to the intervention, and to develop improved markers. Gene-expression analyses will be performed on RNA aliquots obtained from the PAXgene Blood RNA tubesRNA will be extracted using PAXgene silica-membrane technology (PAXgene Blood miRNA Kit) on QIAcube extractor at TRI-UQ. Full transcriptome sequencing will be done at the Institute for Molecular Biosciences Sequencing Facility at University of Queensland. Raw Illumina reads will be mapped to the human reference genome. HTSeq-count will be used to count reads mapping to exons, and to estimate fragments mapping per kilobase per million mapped reads (FPKM). A generalised linear model framework in EdgeR will be used to identify differentially expressed genes between disease groups, considering genes with a false-discovery rate (FDR), as estimated by a Benjamini-Hochberg procedure, to be differentially expressed. We will use Fisher's exact test to calculate whether particular Gene Ontology categories or KEGG pathways are significantly enriched

in differentially expressed genes. We will use the weighted gene coexpression network analysis (WGCNA) tool, to identify co-regulated gene modules, and identify differentially expressed modules by association of module 'eigengene' with disease group. We will *derive minimal signatures distinguishing the disease groups* using a rapid forward-selection algorithm we have previously used. The performance of the signatures will be assessed using cross-validation.

Determination of serum vitamin C levels and their relationship with presence and severity of response to CPB. The unique dataset and biobank acquired thanks to this study will enable to improve our understanding of response to CPB in children. Specifically, we will investigate whether levels of vitamin C in blood are associated with severity after CPB, as postulated by recent studies who have proposed ascorbic acid as a novel treatment. These tests will be performed in batch in Queensland Health and University of Queensland laboratories. Test results will be available to researchers only and will not affect clinical care as performed in batch for research only purpose. The tests will be done on already available study aliquots and will not increase or alter the amount of study blood taken.

7.4 *HEALTH ECONOMIC EVALUATION*

Economic Evaluation. A with-in trial economic evaluation will be undertaken from the health system perspective to compare the cost of providing NO to that of usual care. Resources to be measured and costed will include the number of length of stay (in PICU and/or non-intensive stay), and the length of ECLS treatment. Costs will be assigned by the hospital cost centre or standard national sources (e.g. Independent Hospital Pricing Authority). Both parametric (e.g. ANCOVA) and non-parametric bootstrapping techniques [37] will be employed to compare mean difference in the costs between groups, and to estimate a confidence interval around the mean. It is anticipated that NO intervention will be cost saving to the health system. The cost comparison will inform recommendations on adopting NO in standard CPB practice.

7.5 *NEURODEVELOPMENTAL EVALUATION*

The consent of study participants specifically mentions that families will get contacted a year post intervention for follow-up.

Patients will be followed up at all sites at 12 months post intervention (range of 10 to 15 months post intervention is recommended). The 1-year follow-up will be based on surveys which parents can attend/complete from home (less than 20 mins time commitment required) and will assess Quality of Life (pedsQL), neurodevelopment (Ages and Stages Questionnaire), and socioeconomic status.

In order to obtain more granular information on neurodevelopmental outcomes, families will be offered a full neurodevelopmental assessment as well. This will be restricted to sites where funding and local resources and expertise allow, and coordinated with the local practice of follow-up to reduce time commitment for families.

Neurodevelopmental assessment will be performed by an experienced paediatric neuropsychologist, using the Bayley-III SE, at 12-month post intervention. The Bayley-III is recognised internationally as one of the most comprehensive tools to assess children from as young as one month old and represents the most widely used developmental assessment tool during pre-school which is widely

used in neonatal follow-up across the world. Bayley-III identifies infant and toddler strengths and competencies, as well as their weakness. It also provides a valid and reliable measure of a child's abilities, in addition to giving comparison data for children with high incidence clinical diagnoses. Descriptive statistics will be utilised to report on the baseline characteristics of the study cohort and each subgroup. Proportion of children with a neurodevelopmental deficit will be analysed using a Chi-square/Fisher exact tests. Analysis of association includes both comparisons of measurements and proportions, using confidence intervals of differences as the major method of presentation where possible, otherwise standard techniques such as Mann-Whitney U tests, t-tests and chi-squared tests will be utilised. The raw score for Bayley-III is standardized to a mean of 100 with an SD of 15 (range, 50-150). The standardized score is also classified into the categories of accelerated performance (>115), within normal limits (85-115), and delayed performance (<85). Statistical significance will be set at the 0.05 level for the secondary outcomes. It is anticipated the neurodevelopmental outcomes at 12 months will provide data for feasibility, sample size calculation and design of long term neurodevelopmental assessments within the study towards school age outcomes.

7.6 *MONITORING*

Monitoring:

Each participating site will be monitored in 6 monthly intervals. Data monitoring will be undertaken throughout the trial, based on a data monitoring and auditing plan (DMAP) devised by the study team. The DMAP has been developed in accordance with ICH GCP guidelines and reflects current best practice for data monitoring practices in investigator-initiated trials. The DMAP includes the following components:

- Data verification on all screening data items (i.e. inclusion and exclusion criteria) on a random sample of 5% of ineligible patients from each site;
- Data verification on all screening data items (i.e. inclusion and exclusion criteria) for every enrolled patient;
- Data verification on the stratification used for randomisation and consent data items and data items related to calculation of the primary outcome and secondary outcomes for every enrolled patient; and
- Data verification on key data items relating to cohort descriptors on a random sample of 10% of enrolled patients from each site.

The original study REDCap database was enhanced to facilitate the DMAP. Each site is monitored by a research co-ordinator from a different trial site. This will be undertaken through a combination of on-site monitoring and remote monitoring using the institutional programme to share desktop computer screens. The remote monitoring will not be recorded or stored and the monitored site is in control of the shared patient screen at all times. During the coronavirus-2019 pandemic (COVID-19), sites are only allowed to use remote monitoring. Each relevant data item is verified individually by comparing the entered value with the value in the source documentation. Where discrepancies are found, the site research co-ordinator and monitor discuss and resolve the discrepancies. Once data monitoring is finalised, the patient's REDCap data entry record is locked in preparation for analysis.

Study protocol adherence. Site visits for start up and education to guarantee adherence to the study protocol will be performed at the start and then twice during the study.

8 ADVERSE EVENTS AND RISKS

8.1 Definitions

Adverse Event (AE) is any untoward medical occurrence in a patient enrolled into this study regardless of its causal relationship to study treatment intraoperatively and ***within the first 48 hours postoperatively.***

Serious Adverse Event (SAE**) is** any AE that results in:
- Death
- Is life-threatening
- Requires prolongation of existing hospitalisation
- Results in persistent or significant disability/incapacity
- Requires intervention to prevent permanent impairment or damage

Specific AEs related to NO delivery during CBP: **air embolism, severe hypotension during bypass and increased MetHb (MetHb is >3%).** For the purposes of this study the site investigator is responsible for recording all AEs, regardless of their relationship to study intervention, for the period from randomisation until completion of each individual child's participation in the study.

This study is performed in infants and children undergoing high risk cardiac surgical procedures with death or high risk organ support such as ECLS or renal replacement therapy within the expected outcome. We will report all SAE including deaths and the investigators will give clinical recommendations if the death was unexpected and reason for further investigation by the DSMB.

Additional screening for adverse events will occur at the time of patient discharge from hospital.

8.2 Assessment and documentation of adverse events

The description of each AE on the Case Report Form (CRF) will include all of the following:

- A description of the AE;
- The onset date, duration, date of resolution;
- Severity (mild, moderate or severe);
- Seriousness (i.e. is it an SAE?);
- Any action taken;
- The outcome (recovery, death, continuing, worsening);
- The likelihood of the relationship of the AE to the study treatment (Unrelated, Possible, Probable, Definite).

Changes in the severity of an AE will be recorded on the CRF.

AEs characterised as intermittent will be documented for each episode.

All AEs will be followed to adequate resolution or stabilisation, where possible.

Data Safety Monitoring Board (DSMB). Consists of one statistician, one independent intensive care specialist (from a non-cardiac centre) and one cardiac surgeon. The DSMB will oversee the study performance, recruitment rate and analysis.

8.3 Eliciting adverse event information

Adverse events will be recorded from the time the patient is randomised to the study until end of study participation. Additional screen for AEs will occur at the time of patient discharge from hospital. AEs will be documented from physical examination findings, clinically significant lab results or other documents that are relevant to patient safety.

8.4 Serious adverse event reporting

Any SAE occurring in a study participant will be reported to the HREC within 24-72 hours of detection, in accordance with the safety reporting policy of the HREC. The HREC safety reporting form will be completed, signed and submitted by an investigator.

9 DATA MANAGEMENT

9.1 Data Collection

The investigators are responsible for ensuring the accuracy, completeness, legibility, and timeliness of the data reported. All source documents should be completed in a neat, legible manner to ensure accurate interpretation of data. The investigators will maintain adequate case histories of study participants, including accurate case report forms (CRFs), and source documentation.

9.1.1 Source Data

Source data will be entered directly onto pre-printed CRFs where practical, where this cannot occur in real time, data will be retrospectively entered onto CRFs from hospital records, observation charts and resuscitation flow sheets to complete the required data set.

9.1.2 Data Capture Methods

Data will be prospectively entered into a secure web-based database (REDCap https://redcap.health.uq.edu.au/), hosted by the University of Queensland. Printed paper CRFs will be available if required.

9.2 Data Storage

Hard copy data will be securely stored by the investigating site in a locked cupboard in a secured location. Electronic data will be securely stored on the RedCAP database, hosted by the University of Queensland.

9.3 Sample Storage

Biological sample processing and storage will follow standard operating procedures. The samples are kept in a security protected freezer in the each institution, and will be transferred in batch every 12 months to a dedicated security protected freezer at the Centre for Children`s Health Research

Centre, Brisbane, Australia. The samples will be kept for 10 years after study completion and will be destroyed thereafter. All study blood tests will be labelled with a study number, and not with an identifier containing the identity of the participant, to ensure confidentiality. Any use of the participants blood other than what is approved for this study will require further review by our ethics committee and approval.

9.4 Record Retention

As required by the Queensland State Archivist, all study information and documentation will be securely stored for a period of 15 years after the date of the child's eighteenth birthday. As this trial will recruit participants down to birth age, all records will be securely stored for a total of 33 years before being securely destroyed.

10 STUDY OVERSIGHT

The trial will be overseen by a trial steering committee (TSC), the membership of which will include: the CIA, and at least 2 further CIs. The role of the TSC will be to: monitor and supervise progress of the trial; review at regular intervals relevant information from other sources.

The statistician and secretariat will be appointed once the study has authorisation to commence. Prior to the commencement of the study, the DSMB Terms of Reference (ToR) and Standard Operatine Proceedures (SOP) will be distributed.

The trial will be coordinated at each site by an on-site Study Management Team consisting of, at a minimum, the site PI and a site study coordinator/research assistant, and an expanded group including a representative each of perfusion, PICU, and cardiac surgery. The site PI will be responsible for local oversight of the study including: monitoring safety; ensuring that the study is conducted according to the protocol; and ensuring data integrity. The site PI will review the data for safety concerns and data trends at regular intervals, and will promptly report to the HREC and the central coordinating team any significant protocol deviation or any other significant event or problem that arises during the conduct of the study.

10.1 Quality Control and Quality Assurance

To standardise the collection of data by local investigator teams, training of the site PI and research assistant will be undertaken by the central coordinating team; the site PI and site research assistant will train the remaining local investigator team in particular the senior medical staff conducting consent and randomisation.

Training of the site PI and site research assistant will include all aspects of study conduct.

Set DSMB review points on the progress and safety of the trial are after the primary outcome is known at the interim analysis. While no formal stopping rule will be used, the DSMC may recommend ceasing the trial if there is a statistically significant difference ($p<0.001$) in primary outcome between the treatment groups overall or within pre-specified age subgroups, or in case of serious adverse events. Cessation of the trial may also be recommended if there is equipment failure

or recall, or if other evidence becomes available that would make continuing the trial unethical.

11 STATISTICAL METHOD

11.1 *ANALYSIS PLAN*

Data Analysis. Descriptive statistics will be utilised to report on the baseline characteristics of the total study cohort and each subgroup, as well as by site. The primary outcome measure investigating length of mechanical ventilation will be analysed using a Mann-Whitney test (assuming the data is non-normally distributed). Analysis of secondary outcomes includes both comparisons of measurements and proportions, using confidence intervals of differences as the major method of presentation where possible, otherwise standard techniques such as Mann-Whitney U tests, t-tests and chi-squared tests will be utilised. All analyses will be by intention-to-treat. Statistical significance will be set at the 0.05 level for the primary outcomes. Post-hoc power analyses may be undertaken to determine if results found in sub-group analyses are reliable.

11.2 *SAMPLE SIZE*

Sample Size. Table 2 reports the pilot data, which supports the sample size calculation. Sample size calculations were updated based on individual patient level data from the pilot study to take the distribution of ventilator free days into account. The pilot study showed an approximate 66 hours (0.33 SD) reduction in ventilator-free days (VFD) associated with the study intervention [26]. Based on the primary outcome measure VFD, 1,320 patients (660 per group) would be required to demonstrate a significant increase in VFD assuming a minimally clinically significant small effect size (0.2 SD), 90% power, two-sided alpha level of significance of 5%, 10% withdrawals, and 15% increase in sample size to account for a non-normal distribution of VFD.

12 ETHICS AND DISSEMINATION

12.1 Research Ethics Approval

This protocol and the informed consent document and any subsequent modifications will be reviewed and approved by the human research ethics committee (HREC).A letter of protocol approval by HREC will be obtained prior to the commencement of the study, as well as approval for other study documents participant to HREC review. The pilot study received ethical approval by the ethic committee of the Royal Children's Hospital Melbourne (HREC 33112B, RCH Melbourne). Protocol Version 1 has been approved by Children`s Health Queensland HREC (HREC/17/QRCH/43).

12.2 Modifications to the protocol

This study will be conducted in compliance with the current version of the protocol. Any change to the protocol document or Informed Consent Form that affects the scientific intent, study design, patient

safety, or may affect a participants willingness to continue participation in the study is considered an amendment, and therefore will be written and filed as an amendment to this protocol and/or informed consent form. All such amendments will be submitted to the HREC, for approval prior to becoming effective.

12.3 Protocol Deviations

All protocol deviations must be recorded in the patient record (source document) and on the CRF and must be reported to the PI. Protocol deviations will be assessed for significance by the Principal Investigator. Those deviations deemed to have a potential impact on the integrity of the study results, patient safety or the ethical acceptability of the trial will be reported to the HREC in a timely manner. Where deviations to the protocol identify issues for protocol review, the protocol will be amended as per section 12.2.

12.4 Confidentiality

Participant confidentiality is strictly held in trust by the participating investigators, research staff, and the sponsoring institution and their agents. The study protocol, documentation, data and all other information generated will be held in strict confidence. No information concerning the study or the data will be released to any unauthorised third party, without prior written approval of the sponsoring institution. Clinical information will not be released without written permission of the participant, except as necessary for monitoring by HREC or regulatory agencies.

12.5 Dissemination and translation plan

Given the lead role of the investigators in paediatric intensive medicine their role in regional and state-wide guideline development processes; their involvement in professional colleges and at key educational conferences it is likely that the findings will have nationwide impact across Australia and New Zealand.

Publication in high impact peer-reviewed journals will be sought and presentation at national and international conferences is anticipated. Novel and modern information dissemination strategies will also be used including social media, podcast presentations and Free Open Access Medical education (FOAM) resources to generate discussion and disseminate the outcomes of the study.

REFERENCES

1. Registry, A.N.Z.P.I.C.; Available from: http://www.anzics.com.au/anzpicr.
2. Zahler, S., et al., *Acute cardiac inflammatory responses to postischemic reperfusion during cardiopulmonary bypass.* Cardiovasc Res, 1999. **41**(3): p. 722-30.
3. Levy, J.H. and K.A. Tanaka, *Inflammatory response to cardiopulmonary bypass.* Ann Thorac Surg, 2003. **75**(2): p. S715-20.
4. Duval, E.L., et al., *Pro- and anti-inflammatory cytokine patterns during and after cardiac surgery in young children.* Eur J Pediatr, 1999. **158**(5): p. 387-93.
5. Domanski, M.J., et al., *Association of myocardial enzyme elevation and survival following coronary artery bypass graft surgery.* JAMA, 2011. **305**(6): p. 585-91.
6. Hoffman, T.M., et al., *Efficacy and safety of milrinone in preventing low cardiac output syndrome in infants and children after corrective surgery for congenital heart disease.* Circulation, 2003. **107**(7): p. 996-1002.
7. Duke, T., C. Stocker, and W. Butt, *Monitoring children after cardiac surgery: a minimalist approach might be maximally effective.* Crit Care Resusc, 2004. **6**(4): p. 306-10.
8. Ballweg, J.A., G. Wernovsky, and J.W. Gaynor, *Neurodevelopmental outcomes following congenital heart surgery.* Pediatr Cardiol, 2007. **28**(2): p. 126-33.
9. Cornell, T.T., et al., *Clinical implications and molecular mechanisms of immunoparalysis after cardiopulmonary bypass.* J Thorac Cardiovasc Surg, 2012. **143**(5): p. 1160-1166 e1.
10. Bronicki, R.A., et al., *Dexamethasone reduces the inflammatory response to cardiopulmonary bypass in children.* Ann Thorac Surg, 2000. **69**(5): p. 1490-5.
11. Schroeder, V.A., et al., *Combined steroid treatment for congenital heart surgery improves oxygen delivery and reduces postbypass inflammatory mediator expression.* Circulation, 2003. **107**(22): p. 2823-8.
12. Elhoff, J.J., et al., *Intraoperative Steroid Use and Outcomes Following the Norwood Procedure: An Analysis of the Pediatric Heart Network's Public Database.* Pediatr Crit Care Med, 2016. **17**(1): p. 30-5.
13. Whitlock, R.P., et al., *Methylprednisolone in patients undergoing cardiopulmonary bypass (SIRS): a randomised, double-blind, placebo-controlled trial.* Lancet, 2015. **386**(10000): p. 1243-53.
14. Groom, R.C., et al., *Update on pediatric perfusion practice in North America: 2005 survey.* J Extra Corpor Technol, 2005. **37**(4): p. 343-50.
15. Gaynor, J.W., *The effect of modified ultrafiltration on the postoperative course in patients with congenital heart disease.* Semin Thorac Cardiovasc Surg Pediatr Card Surg Annu, 2003. **6**: p. 128-39.
16. Davies, M.J., et al., *Modified ultrafiltration improves left ventricular systolic function in infants after cardiopulmonary bypass.* J Thorac Cardiovasc Surg, 1998. **115**(2): p. 361-9; discussion 369-70.
17. Hataishi, R., et al., *Inhaled nitric oxide decreases infarction size and improves left ventricular function in a murine model of myocardial ischemia-reperfusion injury.* Am J Physiol Heart Circ Physiol, 2006. **291**(1): p. H379-84.

18. Chello, M., et al., *Nitric oxide modulation of neutrophil-endothelium interaction: difference between arterial and venous coronary bypass grafts.* J Am Coll Cardiol, 1998. **31**(4): p. 823-6.
19. Jones, S.P. and R. Bolli, *The ubiquitous role of nitric oxide in cardioprotection.* J Mol Cell Cardiol, 2006. **40**(1): p. 16-23.
20. Jones, S.P., et al., *Myocardial ischemia-reperfusion injury is exacerbated in absence of endothelial cell nitric oxide synthase.* Am J Physiol, 1999. **276**(5 Pt 2): p. H1567-73.
21. Minamishima, S., et al., *Inhaled nitric oxide improves outcomes after successful cardiopulmonary resuscitation in mice.* Circulation, 2011. **124**(15): p. 1645-53.
22. Schulz, R., M. Kelm, and G. Heusch, *Nitric oxide in myocardial ischemia/reperfusion injury.* Cardiovasc Res, 2004. **61**(3): p. 402-13.
23. Lundberg, J.O., M.T. Gladwin, and E. Weitzberg, *Strategies to increase nitric oxide signalling in cardiovascular disease.* Nat Rev Drug Discov, 2015. **14**(9): p. 623-41.
24. Abadesso, C., et al., *Non-invasive ventilation in acute respiratory failure in children.* Pediatric Reports, 2012. **4**(2): p. e16-e16.
25. Arnold, W.P., et al., *Nitric oxide activates guanylate cyclase and increases guanosine 3':5'-cyclic monophosphate levels in various tissue preparations.* Proc Natl Acad Sci U S A, 1977. **74**(8): p. 3203-7.
26. Sokol, J., S.E. Jacobs, and D. Bohn, *Inhaled nitric oxide for acute hypoxemic respiratory failure in children and adults.* Cochrane Database Syst Rev, 2000(4): p. CD002787.
27. Goldstein, I., et al., *Oral sildenafil in the treatment of erectile dysfunction. Sildenafil Study Group.* N Engl J Med, 1998. **338**(20): p. 1397-404.
28. Gianetti, J., et al., *Supplemental nitric oxide and its effect on myocardial injury and function in patients undergoing cardiac surgery with extracorporeal circulation.* J Thorac Cardiovasc Surg, 2004. **127**(1): p. 44-50.
29. Checchia, P.A., et al., *Nitric oxide delivery during cardiopulmonary bypass reduces postoperative morbidity in children--a randomized trial.* J Thorac Cardiovasc Surg, 2013. **146**(3): p. 530-6.
30. James, C., et al., *Nitric oxide administration during paediatric cardiopulmonary bypass: a randomised controlled trial.* Intensive Care Med, 2016. **42**(11): p. 1744-1752.
31. Beca, J., et al., *Hypothermia for Traumatic Brain Injury in Children-A Phase II Randomized Controlled Trial.* Crit Care Med, 2015. **43**(7): p. 1458-66.
32. Erickson, S., et al., *Acute lung injury in pediatric intensive care in Australia and New Zealand: a prospective, multicenter, observational study.* Pediatr Crit Care Med, 2007. **8**(4): p. 317-23.
33. Schlapbach, L.J., et al., *Mortality related to invasive infections, sepsis, and septic shock in critically ill children in Australia and New Zealand, 2002-13: a multicentre retrospective cohort study.* Lancet Infect Dis, 2015. **15**(1): p. 46-54.
34. Liangos, O., et al., *Whole blood transcriptomics in cardiac surgery identifies a gene regulatory network connecting ischemia reperfusion with systemic inflammation.* PLoS One, 2010. **5**(10): p. e13658.
35. Caputo, M., et al., *Controlled reoxygenation during cardiopulmonary bypass decreases markers of organ damage, inflammation, and oxidative stress in single-ventricle patients undergoing pediatric heart surgery.* J Thorac Cardiovasc Surg, 2014. **148**(3): p. 792-801 e8; discussion 800-1.

36. Ghorbel, M.T., et al., *Controlled reoxygenation cardiopulmonary bypass is associated with reduced transcriptomic changes in cyanotic tetralogy of Fallot patients undergoing surgery.* Physiol Genomics, 2012. **44**(22): p. 1098-106.

37. Efron, B. and R.J. Tibshirani, *An Introduction to the Bootstrap*. 1993, New York: Chapman & Hall.

13 APPENDICES

13.1 Informed consent materials

Master consent form and other related documentation given to participants and authorised surrogates.

13.2 Case Report Forms (CRFs)

13.3 Causality and Assessment of Severity – Adverse Events

The severity of an Adverse Event will be assessed as follows:

- **Mild**: Events that require minimal or no treatment and do not interfere with the patient's daily activities.
- **Moderate**: Events that cause sufficient discomfort to interfere with daily activity and/or require a simple dose of medication.
- **Severe**: Events that prevent usual daily activity or require complex treatment.

The relationship of the event to the study drug will be assessed as follows:

- **Unrelated:** There is no association between the intervention and the reported event. AEs in this category do not have a reasonable temporal relationship to exposure to the intervention, or can be explained by a commonly occurring alternative aetiology.
- **Possible:** The event could have cause or contributed to the AE. AEs in this category follow a reasonable temporal sequence from the time of exposure to the intervention and/or follow a known response pattern to the intervention, but could also have been produced by other factors.
- **Probable:** The association of the event with the study seems likely. AEs in this category follow a reasonable temporal sequence from the time of exposure to the intervention and are consistent with the known adverse effects of the treatment, or judgement based on the investigators clinical experience.
- **Definite:** The AE is a consequence of administration of the intervention. AEs in the category cannot be explained by concurrent illness, progression of disease state or concurrent medication reaction. Such events may be widely documented as having an association with the intervention.

Supplementary Materials to

Neurodevelopmental Outcomes After Nitric Oxide During Cardiopulmonary Bypass for Open Heart Surgery: The NITRIC 12-month outcome study.

Debbie A Long[1-3#*], PhD, Kristen S Gibbons[3*], PhD, Stephen B Horton[4-6], PhD, Kerry Johnson[3], GradCertPaed, David HF Buckley[7], MBChB, Simon Erickson[8], MBBS, Marino Festa[9,10], MD (Res), Yves d'Udekem[5,11], MD, PhD, Nelson Alphonso[12,13], MD, Renate LeMarsney[3], MPH, David S Winlaw[14,15], MbChB, Kate Masterson[6,16], MSc, Kim van Loon[17], MD, PhD, Paul J Young[18-21], PhD, Andreas Schibler[22,23], MD, Luregn J Schlapbach[2,3,24^], MD, PhD, Warwick Butt[5,6,17,21,25^], MD for the NITRIC Study Group, the Australian and New Zealand Intensive Care Society Paediatric Study Group (ANZICS PSG).

Correspondence:
Associate Professor Debbie Long
School of Nursing
Centre for Healthcare Transformation
Queensland University of Technology
Email: da.long@qut.edu.au

Statistical analysis plan.

Statistical analysis plan for the NITric oxide during cardiopulmonary bypass to improve Recovery in Infants with Congenital heart defects (NITRIC) trial

Study design and participants

The NITRIC trial is a 1320-patient international, multicentre, randomised, double-blind trial in infants and children less than two years of age undergoing open heart surgery on CPB. A total of 1320 patients will be recruited across the six paediatric cardiac centres in Australia, New Zealand, and The Netherlands. Eligible patients will be randomly assigned to NO at 20 ppm administered into the CPB oxygenator for the entire duration of CPB versus standard care (no NO into the CPB) in a 1:1 ratio with stratification by age (less than six weeks; greater than or equal to six weeks), single ventricle physiology (present or not present) and study site.

The primary hypothesis is to demonstrate that NO during CPB increases VFDs compared with standard care in eligible infants. The full study protocol has been previously published (13) and specified inclusion and exclusion criteria, randomisation and blinding procedures, interventions, study outcomes including primary and secondary outcomes, process of care measures and physiological descriptors, adverse events, safety data monitoring, sample size, and data collection and management.

The study protocol has been approved by the Children's Health Queensland Human Research Ethics Committee (HREC/17/QRCH/43; original submission approved 6th March 2017). Minor modifications to the original study protocol were reviewed and approved by the HREC and are provided in Supplementary Appendix (Section S1). This SAP is based on version 1.4 of the study protocol.

Sample size

Pilot study data demonstrated a 66 hour (0.33 SD) reduction in VFDs associated with the study intervention [26]. Assuming a minimally clinically significant small effect size (0.2 SD), 90% power, two-sided alpha level of significance of 5%, 10% withdrawals, and 15% increase in sample size to account for a non-normal distribution of VFD, 1,320 patients are required (660 per group).

Randomisation

Randomisation is conducted online through the purpose-built REDCap electronic data capture tool hosted at The University of Queensland (14, 15); the randomisation module can only be accessed by the central study coordinator and the site perfusionist, and only after a patient has been screened, is deemed to have met eligibility criteria and has provided informed consent. A randomisation sequence using variable block randomisation with a 1:1 ratio was generated and loaded into REDCap prior to screening of the first patient. Randomisation is stratified on age (<six weeks, ≥six weeks), cardiac physiology (univentricular, biventricular) and study site. The precise time of randomisation occurs variably prior to starting CPB dependent on site logistics, and the intervention begins with start of CPB.

Outcome measures

The definition and detail of calculation of outcome measures can be found in the Supplementary Appendix (Section S2), alongside the Stata scripts written to calculate these outcomes (available on GitHub (16)). Briefly, the outcomes are:

- Primary outcome measure: VFDs within 28 days from start of CPB
- Secondary outcome measures:

- Individual VFD components (i.e. duration of mechanical ventilation, death within 28 days after CPB start)
- Individual and components of composite measure of LCOS, and/or ECLS within 48 hours after CPB start and/or death within 28 days after CPB start;
- Length of PICU stay after CPB start;
- Length of hospital stay after CPB start;
- Process of care measures:
  - Treatment with ECLS within 48 hours after CPB start;
  - Duration of postoperative time spent with open chest including unplanned chest reopening after CPB start;
  - Treatment and duration of treatment using inhalational nitric oxide postoperatively after CPB start;
  - Treatment and duration of treatment of postoperative renal replacement therapy (includes peritoneal and continuous venous-venous haemodialysis) after CPB start;
- Physiological descriptors:
  - Postoperative troponin levels during the first 24 hours postoperatively;
  - Severity and duration of postoperative organ dysfunction; and
  - Postoperative acute kidney injury (AKI) and serum creatinine levels measured during the first 24 hours.

For the purposes of analysis and reporting, LCOS within 48 hours after CPB start, ECLS within 48 hours after CPB start, death within 28 days after CPB start and length of stay in PICU and hospital are classified as potentially patient-important secondary outcomes. The remaining secondary outcomes will be reported to describe patient physiology and processes of care only.

Data monitoring

Data monitoring is being undertaken throughout the trial, based on a data monitoring and auditing plan (DMAP) devised by the study team. The DMAP was developed in accordance with the ICH E6 (R2) Good Clinical Practice Guideline (17) and reflects current best practice for data monitoring practices in investigator-initiated trials. Briefly, the DMAP includes the following components:

- Data verification on all screening data items (i.e. inclusion and exclusion criteria) on a random sample of 20 ineligible patients (or all patients if less than 20 ineligible patients) from each site;
- Data verification on all screening data items (i.e. inclusion and exclusion criteria) for every enrolled patient;
- Data verification on the stratification used for randomisation and consent data items and data items related to calculation of the primary outcome and secondary outcomes for every enrolled patient; and
- Data verification on key data items relating to cohort descriptors on a random sample of 10% of enrolled patients from each site.

The original study REDCap database was enhanced to facilitate the DMAP. Each Australian and New Zealand site is being monitored by a research co-ordinator from a different trial site. This is being undertaken through a combination of on-site monitoring and remote monitoring using the institutional programme to share desktop computer screens. All sites except one have the required information for physiological data, treatment, outcomes, laboratory values, and patient notes on an electronic health record. One site has parts of the patient data on paper, which requires digital scanning for review. During the coronavirus-2019 pandemic (COVID-19), sites are only allowing remote monitoring. Each relevant data item is verified individually by comparing the entered value with the value in the source documentation. Where

discrepancies are found, the site research co-ordinator and monitor meet to discuss and resolve the discrepancies. Once data monitoring is finalised, the patient's REDCap data entry record is locked in preparation for analysis. The site in The Netherlands is being monitored onsite by an external monitoring company as remote monitoring is not permitted in The Netherlands; during the COVID-19 pandemic, monitoring was paused.

Statistical analysis

Statistical analysis principles

- Analyses will be conducted based on the intention-to-treat principle. Specifically, patients who are eligible, who do not meet any exclusion criteria and who undergo randomisation will be analysed based on the treatment group they were allocated to, independent of the compliance with the treatment delivered.
- Participants who had more than one surgery before their second birthday will only have data related to their first surgery for which study consent was available analysed.
- Statistical tests will be two-sided applying a statistical significance level of 0.05. As we are not correcting for multiplicity when comparing secondary or other outcomes, such results will be considered exploratory and will be reported as point estimates with 95% confidence intervals (CIs).
- If there is missing data for the primary outcome measure for any participants, imputation methods will be used.
- Continuous variables will be assessed for normality; this will be undertaken using visual inspection of histograms and Q-Q plots.
- Standard descriptive statistics will be used when summarising variables; frequencies (percentages) for discrete variables, mean and standard deviation (SD) for continuous

variables, or, if continuous variables are non-normally distributed, median with interquartile range (IQR).

- This analysis plan and the primary manuscript will only include analyses up to 28 days. We will present analyses of postoperative delirium, health care costs, inflammatory markers and long-term outcomes (12 months and later post procedure) separately.
- Pre-planned subgroup analyses will be performed including the pre-defined study strata; these will be executed regardless of any potential treatment effect on the primary or secondary outcomes in the main cohort.
- To ensure transparency and reproducibility, the Stata code that will be used to analyse the final study data is available on GitHub (16).
- The trial statistician will be blinded to the treatment group until the analyses outlined in this SAP have been completed.
- Changes in the analysis plan by the investigators effective after publication of this SAP will be declared as such.

Interim analyses

Two pre-planned interim analyses were performed after the primary outcome measure was finalised for 660 and 1000 patients, respectively. Blinded interim analyses that detailed the primary outcome between treatment groups as well as information on recruitment and adverse events were presented to the Data and Safety Monitoring Board (DSMB) for their consideration. The Haybittle-Peto rule was applied (i.e. a significant p-value less than 0.001 was deemed necessary to warrant consideration of stopping the study early for benefit) (18, 19). The type I error for the final study analyses has not been adjusted to allow for interim analyses.

Datasets analysed

The Consolidated Standards of Reporting Trials (CONSORT) flow diagram will be presented based on all patients who were screened for the study. All other analyses will be performed on eligible patients who underwent randomisation, i.e. the intention-to-treat population independent of compliance with the protocol. If consent is not obtained or withdrawn, data will be excluded from the analyses, unless withdrawn patients permitted the use of data up to the point of withdrawal. The primary dataset for analysis will include baseline variables, surgical data, outcomes, adverse events and protocol deviations. Following completion of the data monitoring process, data and associated data transformation code will be extracted from the study REDCap database in a Stata (StataCorp Pty Ltd, College Station, Texas) format.

Trial profile and overview

Recruitment of patients into the trial will be represented using a flow chart based on the CONSORT guidelines (20) (Figure 1). This will describe screened patients, those meeting exclusion criteria, eligible patients, consent process, and those randomised into each of the study arms, with the documentation of the respective primary outcome. We will report on the start and stop date of the trial and provide the recruitment graph by month including division into the contributing sites.

Patient baseline characteristics

Baseline characteristics at time of randomisation will be reported for each of the two treatment groups (statistical comparison between groups will not be undertaken) (Table 1).

Surgical procedure and intervention characteristics

We will provide details on the surgical procedure by treatment group, including concomitant therapies (Table 2). We will compare characteristics of the procedure that in principle may be altered by the intervention (for example, duration of CPB, duration of aortic cross-clamp) between the two study arms by presenting descriptive statistics along with estimate of difference and 95% confidence interval (CI).

We will report on the compliance with the study drug (NO) using a number of measures, reported for patients randomised to the intervention group only (Table 2). Additionally, we will list protocol deviations relating to the administration of NO in supplementary material.

Outcome measures analysis

Primary outcome measure

The primary outcome measure (VFDs censored at 28 days after CPB start) will be analysed using a Mann-Whitney test with differences between medians calculated by quantile regression using the simplex algorithm, inclusive of stratification variables (age group and physiology) and site included as fixed effects in the model. The effect estimate, corresponding 95% confidence interval (CI) and p-value will be presented. The unadjusted p-value using a Mann-Whitney test will also be presented (Table 3).

Secondary outcome measures

For binary outcome measures (e.g. LCOS), logistic regression analyses will be used including age group and physiology as fixed effects, and site as a random effect, with unadjusted and adjusted odds ratios (ORs) and 95% CIs reported (Table 3). Survival outcomes (length of PICU stay, length of hospital stay) will be visually presented using a Kaplan-Meier plot and a Cox proportional hazard model will be used to assess differences between treatment groups with

treatment group and stratification variables as fixed effects and site as a random effect (i.e. utilising a shared frailty model). The hazard ratio and 95% CI will be presented as an estimate of treatment effect. Continuous outcomes (e.g. PELOD-2, duration of renal replacement therapy) will be analysed using linear regression adjusting for age group and physiology as fixed effects, and site as a random effect, with mean difference and 95% CI reported. If the residuals demonstrate non-normality, quantile regression will instead be used in the same manner as for the primary outcome. Key assumptions of the models will be tested and reported on (for logistic regression: specification, goodness-of-fit, absence of multicollinearity and absence of influential observations; for survival analysis: proportionality assumption, goodness-of-fit; for linear regression: specification, distribution of residuals, homoscedasticity, absence of multicollinearity, linearity).

Safety outcomes

Adverse drug reactions considered to be possibly, probably, or definitively related to the study treatment based on the judgement of the treating local physician, will be reported in supplementary material. Additionally, the proportion of patients with at least one adverse event will be compared between the two treatment groups using logistic regression as described above for secondary outcomes.

Subgroup and sensitivity analyses

We will undertake two subgroup analyses that were pre-defined in the study protocol:

- age: <six weeks versus ≥six weeks; and
- physiology: univentricular versus biventricular.

Subgroup analyses will be undertaken using the same analysis methods described for the primary and secondary outcome measures, with the addition of the subgroup variable and its

related interaction term into the main regression model. For each subgroup, the relevant descriptive statistics will be presented for the primary and secondary outcomes, along with the appropriate measure of effect size (and 95% CI) and interaction effect (and 95% CI and p-value). A Forest plot will be developed to present heterogeneity between the treatment group and subgroup variable, including the p-value, and presented as a supplementary figure.

Additionally, a sensitivity analysis for study outcomes will be undertaken including the following variables: treatment group, duration of CPB, surgical complexity (recorded using the risk adjustment for congenital heart surgery [RACHS] score), blood prime using during surgery, sex and strata variables as fixed effects, and site as a random effect. Results will be presented in the same manner as primary analyses and included in the supplementary material.

Treatment of missing data

Missing data will be imputed for the primary outcome measure for any patients are missing one or more components required to calculate VFDs. Fully conditional specification will be used for imputation; the imputation model will include randomised treatment arm, study site and the two study strata variables. Ten sets of imputed data will be created using the methods described for the primary outcome. A pooled common effect estimate and 95% confidence interval will be generated from the imputed datasets.

List of planned figures

Figure 1. CONSORT participant flow diagram

Figure 2. Cumulative incidence functions for extubation (accounting for mortality) (all patients, and the pre-specified subgroups)

Figure 3. Composite figure of a) bar chart depicting proportion of patients with LCOS, b) mean (and 95% CI) SaO_2-$ScvO_2$ difference, c) boxplot of lactate levels, d) boxplot of VIS score, e) mean (and 95% CI) of creatinine values, over time points 0, 6, 12, 24, 48 hours after PICU admission, f) PELOD-2 score at 0, 24, 48 hours after PICU admission, separated by treatment group

List of planned supplementary material

- Funding sources
- Trial committee and NITRIC trial investigators
- Data and Safety Monitoring Board Terms of Reference
- Data and Auditing Monitoring Plan
- Enrolment statistics by site and country
- List of congenital heart disease conditions and surgeries
- List of protocol violations
- Results of interim analyses
- Consent details
- Volume of blood product transfused between study arms
- Listing of adverse events
- Kaplan-Meier survival curves for length of PICU stay and length of hospital stay
- Results of subgroup analyses
- Forest plot of the treatment effect across subgroups for the primary and secondary outcomes.
- Results of sensitivity analyses

REFERENCES

1. Triedman JK, Newburger JW. Trends in Congenital Heart Disease: The Next Decade. Circulation. 2016;133(25):2716-33.
2. Marelli AJ, Ionescu-Ittu R, Mackie AS, Guo L, Dendukuri N, Kaouache M. Lifetime prevalence of congenital heart disease in the general population from 2000 to 2010. Circulation. 2014;130(9):749-56.
3. Marelli AJ, Mackie AS, Ionescu-Ittu R, Rahme E, Pilote L. Congenital heart disease in the general population: changing prevalence and age distribution. Circulation. 2007;115(2):163-72.
4. Zahler S, Massoudy P, Hartl H, Hahnel C, Meisner H, Becker BF. Acute cardiac inflammatory responses to postischemic reperfusion during cardiopulmonary bypass. Cardiovasc Res. 1999;41(3):722-30.
5. McElhinney DB, Wernovsky G. Outcomes of neonates with congenital heart disease. Current opinion in pediatrics. 2001;13(2):104-10.
6. Ballweg JA, Wernovsky G, Gaynor JW. Neurodevelopmental outcomes following congenital heart surgery. Pediatr Cardiol. 2007;28(2):126-33.
7. Marino BS. New concepts in predicting, evaluating, and managing neurodevelopmental outcomes in children with congenital heart disease. Current opinion in pediatrics. 2013;25(5):574-84.
8. Marino BS, Lipkin PH, Newburger JW, Peacock G, Gerdes M, Gaynor JW, et al. Neurodevelopmental outcomes in children with congenital heart disease: evaluation and management: a scientific statement from the American Heart Association. Circulation. 2012;126(9):1143-72.

9. Brown MD, Wernovsky G, Mussatto KA, Berger S. Long-term and developmental outcomes of children with complex congenital heart disease. Clinics in perinatology. 2005;32(4):1043-57, xi.

10. Kaltman JR, Andropoulos DB, Checchia PA, Gaynor JW, Hoffman TM, Laussen PC, et al. Report of the pediatric heart network and national heart, lung, and blood institute working group on the perioperative management of congenital heart disease. Circulation. 2010;121(25):2766-72.

11. James C, Millar J, Horton S, Brizard C, Molesworth C, Butt W. Nitric oxide administration during paediatric cardiopulmonary bypass: a randomised controlled trial. Intensive Care Med. 2016;42(11):1744-52.

12. Checchia PA, Bronicki RA, Muenzer JT, Dixon D, Raithel S, Gandhi SK, et al. Nitric oxide delivery during cardiopulmonary bypass reduces postoperative morbidity in children--a randomized trial. J Thorac Cardiovasc Surg. 2013;146(3):530-6.

13. Schlapbach LJ, Horton SB, Long DA, Beca J, Erickson S, Festa M, et al. Study protocol: NITric oxide during cardiopulmonary bypass to improve Recovery in Infants with Congenital heart defects (NITRIC trial): a randomised controlled trial. BMJ Open. 2019;9(8):e026664.

14. Harris PA, Taylor R, Minor BL, Elliott V, Fernandez M, O'Neal L, et al. The REDCap consortium: Building an international community of software platform partners. J Biomed Inform. 2019;95:103208.

15. Harris PA, Taylor R, Thielke R, Payne J, Gonzalez N, Conde JG. Research electronic data capture (REDCap)--a metadata-driven methodology and workflow process for providing translational research informatics support. J Biomed Inform. 2009;42(2):377-81.

16. Gibbons KS. NITric oxide during cardiopulmonary bypass to improve Recovery in Infants with Congenital heart defects (NITRIC) trial: Statistical Analysis Code 2020 [Available from: https://github.com/kgibbons44/NITRICAnalysis/.

17. International Council for Harmonisation of Technical Requirements for Pharmaceuticals for Human Use. ICH Harmonised Guideline: Integrated Addendum to ICH E6 (R1): Guideline for Good Clinical Practice E6 (R2). 2016.

18. Haybittle JL. Repeated assessment of results in clinical trials of cancer treatment. The British journal of radiology. 1971;44(526):793-7.

19. Peto R, Pike MC, Armitage P, Breslow NE, Cox DR, Howard SV, et al. Design and analysis of randomized clinical trials requiring prolonged observation of each patient. I. Introduction and design. Br J Cancer. 1976;34(6):585-612.

20. Schulz KF, Altman DG, Moher D, Group C. CONSORT 2010 statement: updated guidelines for reporting parallel group randomised trials. BMJ. 2010;340:c332.

21. Hoffman TM, Wernovsky G, Atz AM, Kulik TJ, Nelson DP, Chang AC, et al. Efficacy and safety of milrinone in preventing low cardiac output syndrome in infants and children after corrective surgery for congenital heart disease. Circulation. 2003;107(7):996-1002.

22. Gaies MG, Jeffries HE, Niebler RA, Pasquali SK, Donohue JE, Yu S, et al. Vasoactive-inotropic score is associated with outcome after infant cardiac surgery: an analysis from the Pediatric Cardiac Critical Care Consortium and Virtual PICU System Registries. Pediatr Crit Care Med. 2014;15(6):529-37.

23. Gaies MG, Gurney JG, Yen AH, Napoli ML, Gajarski RJ, Ohye RG, et al. Vasoactive-inotropic score as a predictor of morbidity and mortality in infants after cardiopulmonary bypass. Pediatr Crit Care Med. 2010;11(2):234-8.

24. Leteurtre S, Duhamel A, Salleron J, Grandbastien B, Lacroix J, Leclerc F, et al. PELOD-2: an update of the PEdiatric logistic organ dysfunction score. Crit Care Med. 2013;41(7):1761-73.

25. Kaddourah A, Basu RK, Bagshaw SM, Goldstein SL. Epidemiology of Acute Kidney Injury in Critically Ill Children and Young Adults. N Engl J Med. 2017;376(1):11-20.

Figure 1. Draft CONSORT participant flow diagram

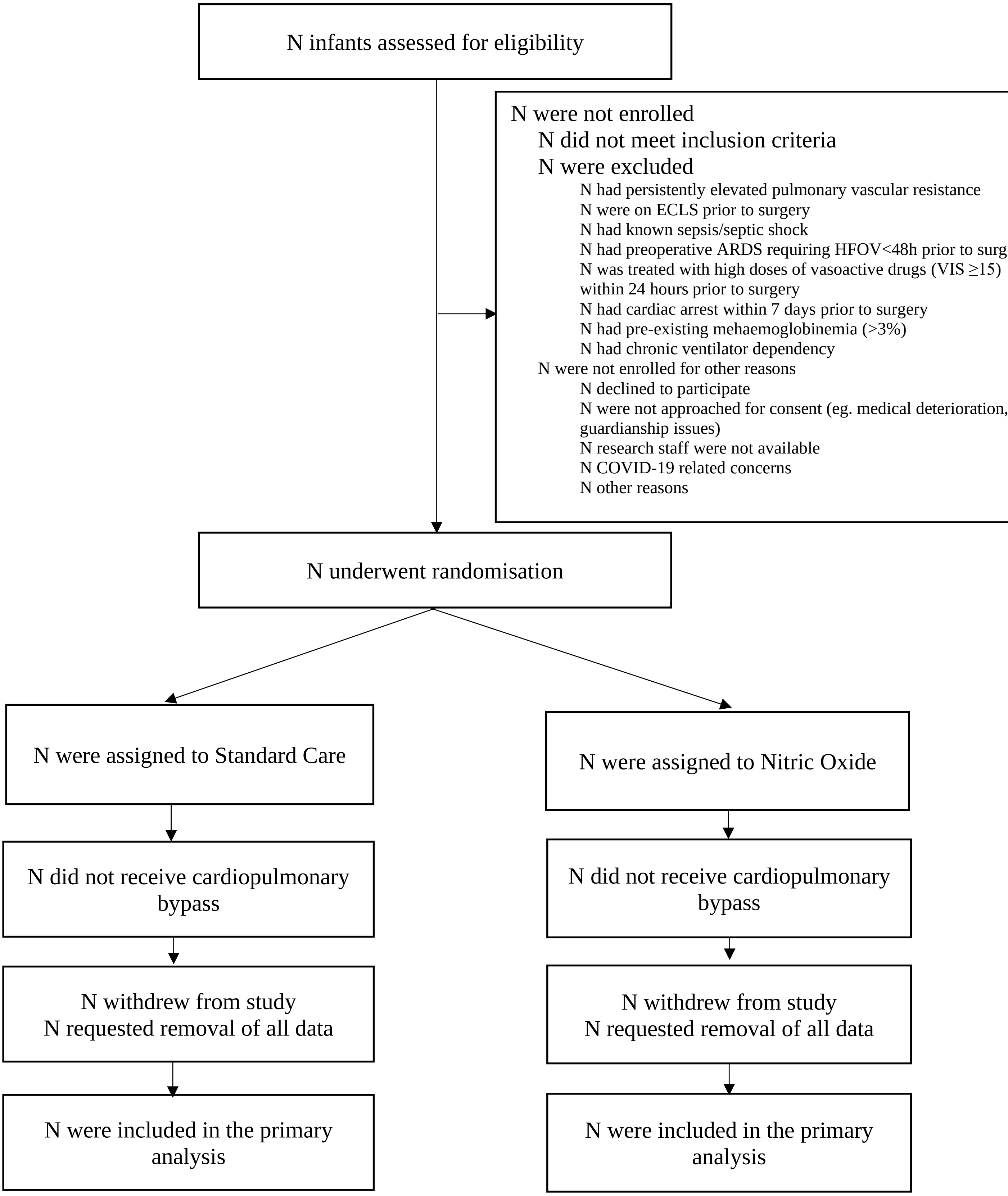

Table 1. Baseline characteristics of infants enrolled in NITRIC trial

**Characteristic**	**Standard Care N=**	**Nitric Oxide N=**
**Age at randomisation (weeks)*** *median (IQR)*		
< 6 weeks *n (%)*		
≥ 6 weeks *n (%)*		
**Weight (kg)** *median (IQR)*		
**Female sex** *n (%)*		
**Ethnicity**		
Caucasian *n (%)*		
Aboriginal/Torres Strait Islander *n (%)*		
Asian *n (%)*		
Maori/Pacific Islander *n (%)*		
Mixed/Other *n (%)*		
**Congenital heart disease**		
Univentricular* *n (%)*		
Biventricular* *n (%)*		
History of previous cardiac surgery on CPB *n (%)*		
Right-sided obstructive lesions *n (%)*		
Tetralogy of Fallot *n (%)*		
Double outlet right ventricle (DORV) *n (%)*		
Pulmonary stenosis/atresia *n (%)*		
Tricuspid stenosis/atresia *n (%)*		
Other *n (%)*		
Left-sided obstructive lesions *n (%)*		
Coarctation *n (%)*		
Interrupted aortic arch *n (%)*		
Hypoplastic aortic arch *n (%)*		
HLHS *n (%)*		
Other *n (%)*		
Shunt lesions *n (%)*		
ASD *n (%)*		
VSD *n (%)*		
AVSD *n (%)*		
Truncus *n (%)*		
TGA *n (%)*		
Persistent ductus arteriosus *n (%)*		
Other *n (%)*		
Various lesions *n (%)*		
Double-inlet left ventricle (DILV) *n (%)*		
TAPVD *n (%)*		
Other *n (%)*		
**Current ICU admission and treatments prior to heart surgery**		

**Characteristic**	**Standard Care N=**	**Nitric Oxide N=**
Intensive care admission *n (%)*		
Duration of ICU stay (days) *median (IQR)*		
Treatments		
Invasive ventilation *n (%)*		
Duration of invasive ventilation (days) *median (IQR)*		
Tracheostomy *n (%)*		
Inotropes *n (%)*		
Prostaglandin *n (%)*		
Steroids *n (%)*		
Afterload reducing agents *n (%)*		
Inhaled nitric oxide *n (%)*		
Sildenafil *n (%)*		
**Comorbidities**		
POPC		
Good/normal *n (%)*		
Functionally normal *n (%)*		
Mild overall disability *n (%)*		
Moderate overall disability *n (%)*		
Severe overall disability *n (%)*		
Coma/vegetative state *n (%)*		
Brain death *n (%)*		
Unknown *n (%)*		
Congenital syndrome *n (%)*		
Trisomy 21 *n (%)*		
22q11 *n (%)*		
Turner *n (%)*		
Noonan *n (%)*		
VACTERL *n (%)*		
CHARGE *n (%)*		
Other syndrome *n (%)*		
**Country of hospital**		
Australia *n (%)*		
New Zealand *n (%)*		
Netherlands *n (%)*		
**Surgical complexity**		
RACHS score *median (IQR)*		
RACHS-1 *n (%)*		
RACHS-2 *n (%)*		
RACHS-3 *n (%)*		
RACHS-4 *n (%)*		
RACHS-5 *n (%)*		
RACHS-6 *n (%)*		

**Characteristic**	**Standard Care N=**	**Nitric Oxide N=**
**Surgical procedure**		
Tetralogy repair *n (%)*		
Norwood procedure *n (%)*		
Bicavopulmonary shunt *n (%)*		
Right ventricular to pulmonary artery shunt/conduit *n (%)*		
Fontan completion *n (%)*		
Arterial switch operation *n (%)*		
ASD repair *n (%)*		
VSD repair *n (%)*		
AVSD repair *n (%)*		
Aortic arch repair *n (%)*		
Coarctation repair *n (%)*		
Truncus repair *n (%)*		
Pulmonary artery band *n (%)*		
Ross procedure *n (%)*		
Left ventricular outflow tract surgery *n (%)*		
Right ventricular outflow tract surgery *n (%)*		
Valve surgery *n (%)*		
Valve repair *n (%)*		
Anomalous pulmonary vein repair *n (%)*		
Heart Transplant *n (%)*		
Other *n (%)*		

*used for stratification

SD standard deviation; IQR interquartile range; POPC Pediatric Overall Performance Category; CPB cardiopulmonary bypass; HLHS hypoplastic left heart syndrome; ASD atrial septal defect; VSD ventricular septal defect; AVSD atrioventricular septal defect; TGA transposition of the great arteries; TAPVD total anomalous pulmonary venous drainage; ICU intensive care unit; VACTERL vertebral defects, anal atresia, cardiac defects, tracheo-esophageal fistula, renal anomalies, and limb abnormalities; CHARGE coloboma, heart defects, atresia choanae (also known as choanal atresia), growth retardation, genital abnormalities, and ear abnormalities; RACHS risk adjustment for congenital heart surgery

Table 2. Surgical and perioperative characteristics of infants enrolled in NITRIC trial

Characteristic	Nitric Oxide N=	Standard Care N=	Difference (95% CI)
**Cardiopulmonary bypass characteristics**			
Blood prime *n (%)*			
CPB duration (min) *median (IQR)*			
CPB <60minutes *n (%)*			
CPB ≥60minutes *n (%)*			
Cross-clamp *n (%)*			
Cross-clamp (min) *median (IQR)*			
Number of CPB runs			
1 *n (%)*			
2 *n (%)*			
3 *n (%)*			
≥4 *n (%)*			
Deep hypothermic arrest *n (%)*			
Duration of deep hypothermic arrest (min) *median (IQR)*			
Antegrade cerebral perfusion *n (%)*			
Modified ultrafiltration used *n (%)*			
Slow continuous ultrafiltration used *n (%)*			
**Blood products received in theatre**			
Red blood cells (mL/kg) *median (IQR)*			
Whole blood (mL/kg) *median (IQR)*			
Platelets (mL/kg) *median (IQR)*			
Fresh frozen plasma (mL/kg) *median (IQR)*			
Cryoprecipitate (mL/kg) *median (IQR)*			
**Drug treatments received in theatre**			
Intravenous steroids *n (%)*			

Inhaled NO *n (%)*			
**Administration of study drug (nitric oxide)**			
Time from start of CPB to start of NO (min) *mean (SD)/median (IQR)*			
Duration of NO on CPB (min) *median (IQR)*			
Proportion of time spent on CPB with NO* *mean (SD)/median (IQR)*			
Change in methaemoglobin level (%) between start and post-CPB *mean (SD)/median (IQR)*^			
Change in methaemoglobin level (%) between start and post-CPB >3%^ *n (%)*			

* if multiple CPB runs during the same surgery, summarising every CPB run individually; ^ calculated as post-CPB methaemoglobin – pre-CPB methaemoglobin

IQR interquartile range; CPB cardiopulmonary bypass; SD standard deviation; NO nitric oxide

Table 3. Primary and secondary outcomes per intention-to-treat analysis

Outcome	Standard Care N=	Nitric Oxide N=	Unadjusted p-value	Estimate of Difference (95% CI)	Adjusted p-value
**Primary Outcome**					
Ventilator-free days *median (IQR)*					
**Secondary Outcomes**					
Duration of invasive ventilation *median (IQR)*					
Low cardiac output syndrome, need for ECLS or death *n (%)*					
Low cardiac output syndrome *n (%)*					
ECLS *n (%)*					
Death *n (%)*					
Length of stay in PICU *median (IQR)*					
Length of stay in hospital *median (IQR)*					
Process of Care Measures					
Duration of time with open chest post-operatively *median (IQR)*					
Treated with inhaled Nitric Oxide post-operatively *n (%)*					
Duration of inhaled Nitric Oxide *median (IQR)*					
Treated with renal replacement post-operatively *n (%)*					
Duration of renal replacement *median (IQR)*					
Physiological Descriptors					
Organ dysfunction post-operatively (PELOD-2)					
PICU admission *median (IQR)*					
24 hours *median (IQR)*					
48 hours *median (IQR)*					
Troponin post-operatively					
PICU admission *median (IQR)*					

24 hours post-PICU admission *median (IQR)*					
Creatinine					
PICU admission *mean (SD)*					
24 hours post-PICU admission *mean (SD)*					
Acute kidney injury: PICU admission *n (%)*					
Stage 1 *n (%)*					
Stage 2 *n (%)*					
Stage 3 *n (%)*					
Acute kidney injury: 24 hours *n (%)*					
Stage 1 *n (%)*					
Stage 2 *n (%)*					
Stage 3 *n (%)*					
Acute kidney injury: 48 hours *n (%)*					
Stage 1 *n (%)*					
Stage 2 *n (%)*					
Stage 3 *n (%)*					

SD standard deviation; IQR interquartile range; ECLS extracorporeal life support; PICU paediatric intensive care unit; CI confidence interval

Supplementary Appendix

S1. List of approved protocol modifications

Version Number	Approval Date	List of Modifications
1 (Original)	06/03/2017	-
1.1	16/07/2017	• Addition of study sites (Children's Hospital Westmead, Sydney; Perth Children's Hospital)<br>• Addition of investigators<br>• Inclusion of planned secondary analyses<br>• Change to randomisation stratification<br>• Inclusion of additional exclusion criteria<br>• Consent form modification<br>• Further detail on instructions on gas flow across all sites, safety cut offs (Met Hb)<br>• Further detail on data collection and blood sampling procedures
1.2	20/11/2017	• Addition of study site (Utrecht Medical Centre, The Netherlands)<br>• Clarification on censoring outcomes at 28 days<br>• Inclusion of additional exclusion criteria<br>• Further detail on consent processes
1.3	18/07/2018	• Addition of neurodevelopment assessment with funding infrastructure<br>• Addition of socioeconomic Survey<br>• Addition of hyperoxia as pre-planned analyses<br>• Revision of study sample size
1.4	10/10/2018	• Updating information on Queensland Children's Hospital<br>• Accessing medical records from other healthcare facilities<br>• Blood collection for inflammatory biomarkers optional on consent form

S2. Definition of outcomes

Primary Outcome

The primary outcome of VFD will be calculated as the sum of all episodes of invasive respiratory support, which require an endotracheal tube in situ, from start of CPB and censored at 28 days after CPB start. Patients who die within this time period will be assigned a value of zero for the VFD outcome. Ventilated days do not include non-invasive ventilation or high-flow nasal cannulae. Patients who receive a tracheostomy will be counted as ventilated as long as they receive positive pressure ventilation through the tracheostomy. The individual components of VFDs (duration of invasive respiratory support from start of CPB within the first 28 days, and death within the first 28 days) will also be presented.

Secondary Outcomes

The main secondary outcome is defined by a composite of presence of LCOS, extracorporeal life support (ECLS), or all-cause death.

- The presence of LCOS (21) will be calculated as a binary variable by evaluating the relevant data items at the following time-points: admission to PICU, and after six hours, 12 hours, 24 hours, and 48 hours post-PICU admission, or until the patient is discharged from PICU (whichever occurs first). Criteria for LCOS include a blood lactate level (arterial where available, if not then venous) >4 mmol/L and the presence of an oxygen extraction of greater than 35% (SaO_2-$ScvO_2$ gradient >35%), or a high inotrope requirement operationalised as Vasoactive-Inotrope Score (VIS) ≥15 (22, 23). If these criteria are met for at least one of the above listed timepoints, the presence of LCOS is confirmed. If individual data items required for the calculation of LCOS are missing, then the patient will be assumed not to have had LCOS.

- ECLS post-CPB during the first 48 hours after CPB start.
- Mortality is defined as all-cause death within the first 28 days after CPB start.

Additional secondary outcomes include PICU and hospital length of stay, which will be measured from start time of CPB. In patients discharged alive from the PICU to the ward and readmitted within 72 hours the subsequent PICU admission duration will be added to the index admission. Length of stay (both PICU and hospital) will be censored at 28 days or when the patient was last alive, whichever occurs earlier. Patients who die during their PICU or hospital admission will be assigned the time to event.

Process of care measures will include the use of ECLS (as defined above); duration of time with open chest post-operatively within the first 28 days (as all surgeries will have an open chest during theatre, the time measure will start with admission to PICU, and will include time spent with open chest in cases of emergency secondary reopening); treatment and duration of treatment with inhalational NO from admission to PICU post-operatively within the first 28 days; and treatment and duration of treatment with renal replacement from admission to PICU post-operatively within the first 28 days (defined as the use of peritoneal dialysis or continuous renal replacement therapy [CRRT]). Duration of treatment with inhalational NO post-operatively and duration of treatment with renal replacement post-operatively will be restricted to those patients receiving these treatments.

Organ dysfunction will be assessed at admission to PICU, after 24 hours and after 48 hours, and will be defined by using the cardiovascular, respiratory, renal, neurological, and haematological components of the Paediatric Logistic Organ Dysfunction-2 (PELOD-2) scores (24). If score items are missing then it will be assumed that value was normal for the purpose of the score.

Postoperative Acute Kidney Injury was assessed using serum creatinine levels to classify according to Kidney Disease: Improving Global Outcomes (KDIGO) criteria (25). Because no baseline creatinine values were available, we applied the age-specific thresholds used in PELOD-2 to define the presumed baseline creatinine values. KDIGO Stage 1 was defined as an increase in creatinine to 1.5 to 1.9 times the presumed baseline; Stage 2 as an increase 2.0 to 2.9 times; and KDIGO 3 as an increase ≥3.0 baseline and/or the use of RRT.
