# Supplementary material for: Neurodevelopmental Outcomes After Nitric Oxide During Cardiopulmonary Bypass for Open Heart Surgery: A Randomized Clinical Trial
Source: JAMA Netw Open. 2025 Feb 5;8(2):e2458040. doi: 10.1001/jamanetworkopen.2024.58040 (PMC11800016; doi:10.1001/jamanetworkopen.2024.58040)
Supplement: Supplement 2. — eMethods eReferences eTable 1. Baseline Characteristics of Children Lost to Follow-Up Compared With Those Included in NITRIC 12-Month Follow-Up eTable 2. Additional Sociodemographic Characteristics of Children and Their Parents/Caregivers Who Participated in NITRIC 12-Month Follow-Up eTable 3. Listing of Sociodemographic Data Contained in ‘Other’ Categories eTable 4. Sensitivity Analysis for Comparison of Primary and Secondary Outcomes eTable 5. Adjusted Estimates of Difference for Total ASQ-3 Score and Total PedsQL Score eTable 6. Factors Associated With Neurodevelopmental Outcome (ASQ-3 Total Score at 12 Months) eTable 7. Factors Associated With Health-Related Quality of Life Outcome (PedsQL Total Score at 12 Months) [file jamanetwopen-e2458040-s002.pdf]

Supplemental Online Content

Long DA, Gibbons KS, Horton SB et al. Neurodevelopmental outcomes after nitric oxide during cardiopulmonary bypass for open heart surgery: a randomized clinical trial. *JAMA Netw Open*. 2025;8(2):e2458040. doi:10.1001/jamanetworkopen.2024.58040

eMethods

eReferences

eTable 1. Baseline Characteristics of Children Lost to Follow-Up Compared With Those Included in NITRIC 12-Month Follow-Up

eTable 2. Additional Sociodemographic Characteristics of Children and Their Parents/Caregivers Who Participated in NITRIC 12-Month Follow-Up

eTable 3. Listing of Sociodemographic Data Contained in "Other" Categories

eTable 4. Sensitivity Analysis for Comparison of Primary and Secondary Outcomes

eTable 5. Adjusted Estimates of Difference for Total ASQ-3 Score and Total PedsQL Score

eTable 6. Factors Associated With Neurodevelopmental Outcome (ASQ-3 Total Score at 12 Months)

eTable 7. Factors Associated with Health-Related Quality of Life Outcome (PedsQL Total Score at 12 Months)

This supplemental material has been provided by the authors to give readers additional information about their work.

© 2025 Long DA et al. *JAMA Network Open*.

eMethods

Study Outcomes

The primary outcome was neurodevelopment at 12 months post randomization. Neurodevelopment was defined as the total score of the Ages and Stages Questionnaire, Third Edition (ASQ-3).[1] The ASQ-3 is a validated parent-completed questionnaire for children aged 1 to 66 months, developed to screen for global development. It provides 21 age-specific questionnaires that consists of 30 statements about the child's present functioning categorized into five developmental domains of communication, gross motor, fine motor, problem-solving and personal social. The ASQ-3 is valid as a parent-completed screening instrument in children with CHD.[2] Parents were asked to indicate if their child achieved a specific behavior or milestone by answering either 'yes' (ten points), 'sometimes' (five points) or 'not yet' (zero points). Scores for all the questions in a domain are summed up for a maximum of 60 points per domain. The total score was formed by summing each of the domain scores (maximum of 300 points), with lower scores indicating more difficulties. The ASQ-3 has been validated for its accuracy in identifying developmental delay in children with congenital heart disease who have undergone surgical repair.[2,3] Baseline ASQ-3 scores were also collected after surgery to capture pre-morbid neurodevelopment.

Secondary outcomes included severe neurodevelopmental impairment, severe neurodevelopmental impairment per domain, poor HRQoL, poor HRQoL per physical and psychosocial subscales, and proportion of functional status worse than or equal to moderate/severe. Total HRQoL and total HRQoL per subscale were also explored. Severe neurodevelopmental impairment was defined as scores > 2 standard deviations (SD) below the published normative sample mean (United States [US] based cohort).[4] HRQoL was defined as the total score of the Pediatric Quality of Life Inventory (PedsQL™).[5,6] The PedsQL™ is a validated parent-completed questionnaire for children aged 1 month to 18 years. It consists of

© 2025 Long DA et al. *JAMA Network Open*.

23-38 statements (depending on the child's age) about the child's physical functioning and psychosocial functioning (comprising of emotional, social and cognitive/school functioning) and has been validated in children with CHD. [7] Parents answer questions based on a 5-point Likert item (0, not a problem; 4, almost always a problem). Items are reverse-scored and linearly transformed to a scale of 0-100, with higher transformed scores indicating a better HRQoL. The proportion of children with poor HRQoL was defined as scores > 2 SD below population norms (US based cohorts). [6,8] Functional status was assessed using the Pediatric Outcome Performance Category (POPC). [9] The POPC provides an estimate of the overall disability of children surviving their PICU admission, determined from six categories based upon their age-appropriate functional outcomes (ranging from good/functionally normal, to brain death). The POPC is a valid clinician-completed instrument in children with CHD. [10]

Two additional outcomes were analysed and reported – mortality at 12 months post-randomisation, and a composite outcome of mortality or severe neurodevelopmental impairment (two or more ASQ-3 domains ≥ 2SD below mean). These variables were not pre-specified, but instead constructed during analyses to assess the impact of truncation due to death.

Approach to Missing Data

Where present, missing data is reported in tables. While there is an extremely low level of missing data for baseline characteristics and early outcomes, due to the proportion of participants lost to follow-up, we undertook additional sensitivity analyses to assess the effect of truncation due to death (using the two additional outcome measures: mortality at 12 months, and composite mortality and neurodevelopmental impairment), and missing data due to non-response. For patients with missing data for the primary outcome, ASQ-3 Total Score at 12 months, multiple imputation was undertaking using the chained equation method. After assessing missing data, and clinically relevant differences in characteristics between respondents

© 2025 Long DA et al. *JAMA Network Open*.

and those lost to follow-up, the following variables were included as predictors in the multiple imputation model: study group, site, stratification variables (age at randomization and lesion type), prematurity, pre-surgical congenital syndrome, duration of bypass, presence of LCOS at 48 hours post-randomization, need for extra corporeal life support within the first 48 hours, ethnicity, ventilator free days, and RACHS (risk adjustment in congenital heart surgery) score. Fifty imputed datasets were used, generated using predictive mean matching. Using these datasets, the primary analysis was repeated with results combined from the imputed datasets according to Rubin's rules[11].

© 2025 Long DA et al. *JAMA Network Open*.

eReferences

1. Squires J, Bricker D, Potter L. Revision of a parent-completed developmental screening tool: Ages and Stages Questionnaires. Journal of Pediatric Psychology. 1997;22(3):313-328.

2. Lépine J, Gagnon K, Prud'homme J, et al. Utility of the ages and stages questionnaires 3rd edition for developmental screening in children with surgically repaired congenital heart disease. Developmental Neurorehabilitation. 2022;25(2):125-132.

3. Noeder MM, Logan BA, Struemph KL, et al. Developmental screening in children with CHD: ages and stages questionnaires. Cardiology in the Young. 2017;27(8):1447-1454.

4. Squires J, Potter L, Bricker D. The ASQ user's guide for the Ages & Stages Questionnaires: A parent-completed, child-monitoring system. Paul H Brookes Publishing; 1995.

5. Varni JW, Seid M, Kurtin PS. PedsQL™ 4.0: Reliability and validity of the Pediatric Quality of Life Inventory™ Version 4.0 Generic Core Scales in healthy and patient populations. Medical Care. 2001:800-812.

6. Varni JW, Limbers CA, Neighbors K, et al. The PedsQL™ Infant Scales: feasibility, internal consistency reliability, and validity in healthy and ill infants. Quality of Life Research. 2011;20:45-55.

7. Uzark K, Jones K, Burwinkle TM, Varni JW. The Pediatric Quality of Life Inventory™ in children with heart disease. Progress in Pediatric Cardiology. 2003;18(2):141-149.

8. Varni JW, Burwinkle TM, Seid M, Skarr D. The PedsQL™* 4.0 as a pediatric population health measure: feasibility, reliability, and validity. Ambulatory Pediatrics. 2003;3(6):329-341.

9. Fiser DH. Assessing the outcome of pediatric intensive care. The Journal of Pediatrics. 1992;121(1):68-74.

10. Kabbani MS, Alsumih NA, Alsadun SA, Hamadah HK. Five-year survival, performance, and neurodevelopmental outcome following cardiopulmonary resuscitation after pediatric cardiac surgery, preliminary investigation in a single-center experience. Journal of the Saudi Heart Association. 2019;31(4):161-169.

11. Little R, Rubin D. Multiple imputation for nonresponse in surveys. John Wiley & Sons, Inc doi. 1987;10:9780470316696.

© 2025 Long DA et al. *JAMA Network Open*.

eTable 1. Baseline Characteristics of Children Lost to Follow-Up Compared With Those Included in NITRIC 12-Month Follow-Up

**Characteristic**	**12-month Follow-up N=927**	**Lost to Follow-up N=391**
**Age at randomization (weeks)**[a] *median (IQR)*	13.3 (1.6 to 27.0)	17.0 (6.2 to 35.6)
< 6 weeks *n (%)*	329 (35.5)	97 (24.8)
≥ 6 weeks *n (%)*	598 (64.5)	294 (75.2)
**Weight (kg)** *median (IQR)*	4.6 (3.5 to 6.6)	5.3 (3.6 to 7.4)
**Sex** *n (%)*		
Female	411 (44.3)	155 (39.6)
Male	516 (55.7)	236 (60.4)
**Ethnicity**[b] *n (%)*		
Aboriginal/Torres Strait Islander	17 (1.8)	21 (5.4)
Asian	102 (11.0)	40 (10.2)
Māori/Pacific Islander Peoples	68 (7.3)	63 (16.1)
Multiethnic/Other[c]	101 (10.9)	64 (16.4)
White	639 (68.9)	203 (51.9)
**NITRIC trial treatment group**		
Nitric oxide	462 (49.8)	196 (50.1)
Standard care	465 (50.2)	195 (49.9)
**Congenital heart disease**[d]		
Univentricular[a] *n (%)*	108 (11.7)	32 (8.2)
Biventricular[a] *n (%)*	819 (88.4)	359 (91.8)
History of previous cardiac surgery on CPB *n (%)*	74 (8.0)	38 (9.7)
Shunt lesions *n (%)*	612 (66.0)	263 (67.3)
Ventricular septal defect *n (%)*	368 (39.7)	169 (43.2)
Atrial septal defect *n (%)*	153 (16.5)	58 (14.8)
Transposition of the great arteries *n (%)*	139 (15.0)	52 (13.3)
Atrioventricular septal defect *n (%)*	80 (8.6)	34 (8.7)
Truncus arteriosus *n (%)*	13 (1.4)	3 (0.8)
Persistent ductus arteriosus *n (%)*	3 (0.3)	2 (0.5)
Other[e] *n (%)*	9 (1.0)	3 (0.8)
Right-sided obstructive lesions *n (%)*	272 (29.4)	133 (34.0)
Tetralogy of Fallot *n (%)*	146 (15.8)	78 (20.0)
Pulmonary stenosis/atresia *n (%)*	103 (11.1)	41 (10.5)
Other[e] *n (%)*	25 (2.7)	12 (3.1)
Tricuspid stenosis/atresia *n (%)*	17 (1.8)	3 (0.8)
Double outlet right ventricle *n (%)*	11 (1.2)	7 (1.8)
Left-sided obstructive lesions *n (%)*	206 (22.2)	58 (14.8)
Hypoplastic aortic arch *n (%)*	127 (13.7)	29 (7.4)
Hypoplastic left heart syndrome *n (%)*	39 (4.2)	11 (2.8)
Coarctation *n (%)*	5 (0.5)	4 (1.0)
Interrupted aortic arch *n (%)*	4 (0.4)	3 (0.8)

© 2025 Long DA et al. *JAMA Network Open*.

Characteristic	12-month Follow-up N=927	Lost to Follow-up N=391
Other[e] *n (%)*	49 (5.3)	18 (4.6)
Various lesions *n (%)*	49 (5.3)	17 (4.4)
Total anomalous pulmonary venous drainage *n (%)*	30 (3.2)	8 (2.1)
Double-inlet left ventricle *n (%)*	9 (1.0)	6 (1.5)
Other[e] *n (%)*	13 (1.4)	4 (1.0)
**Surgical complexity**		
RACHS score *median (IQR)*	3 (2, 3)	2 (2, 3)
RACHS-1 *n (%)*	30 (3.2)	12 (3.1)
RACHS-2 *n (%)*	433 (46.7)	217 (55.5)
RACHS-3 *n (%)*	249 (26.9)	89 (22.8)
RACHS-4 *n (%)*	179 (19.3)	61 (15.6)
RACHS-5 *n (%)*	4 (0.4)	1 (0.3)
RACHS-6 *n (%)*	32 (3.5)	11 (2.8)
**Comorbidities**		
Pre-surgical Modified Pediatric Overall Performance Category *n (%)*		
Good/normal/Functionally normal	550 (60.7)	259 (68.0)
Mild overall disability	144 (15.9)	44 (11.6)
Moderate overall disability	177 (19.5)	65 (16.6)
Severe overall disability	35 (3.9)	13 (3.3)
Coma/vegetative state	0	0
Brain death	0	0
Congenital syndrome[d]	152 (16.4)	75 (19.2)
Trisomy 21 *n (%)*	75 (8.1)	40 (10.2)
22q11 *n (%)*	22 (2.4)	10 (2.6)
VACTERL *n (%)*	7 (0.7)	4 (1.0)
Noonan *n (%)*	4 (0.4)	2 (0.5)
Turner *n (%)*	2 (0.2)	1 (0.3)
CHARGE *n (%)*	1 (0.1)	0 (0.0)
Other syndrome[f] *n (%)*	42 (4.5)	19 (4.9)
**Country of hospital *n (%)***		
Australia	693 (74.8)	293 (72.4)
New Zealand	180 (19.4)	81 (20.7)
Netherlands	54 (5.8)	27 (6.9)
**ICU Outcomes**		
Ventilator-free days *median (IQR)*	26.3 (24.7 to 27.2)	26.9 (24.9 to 27.4)
Duration of invasive ventilation (days) *median (IQR)*	1.7 (0.8 to 3.3)	1.1 (0.6 to 3.1)
Low cardiac output syndrome[g], need for extra corporeal life support or death *n (%)*	186 (20.1)	72 (18.4)
Length of stay in ICU (days) *median (IQR)*	3.0 (1.9 to 5.9)	2.8 (1.9 to 5.1)
Length of stay in hospital (days) *median (IQR)*	9.8 (6.8 to 18.0)	8.0 (6.0 to 14.8)
Acute kidney injury at 48 hours *n (%)*	169 (18.2)	52 (13.3)

© 2025 Long DA et al. *JAMA Network Open*.

Abbreviations: CHARGE, coloboma, heart defects, atresia choanae (also known as choanal atresia), growth retardation, genital abnormalities, and ear abnormalities; CPB, cardiopulmonary bypass; ICU, intensive care unit; RACHS, risk adjustment in congenital heart surgery; VACTERL, vertebral defects, anal atresia, cardiac defects, tracheo-esophageal fistula, renal anomalies, and limb abnormalities.

[a]Used for stratification.

[b]Ethnicity was self-reported by the parent or guardian of the child.

[c]Ethnicities included in the "multiethnic/other" category were predominantly multiethnic children, with primary "other" ethnicities reported as Arabian (n=39), Indian (n=25) and African (n=14)

[d]Patients may have more than 1 type of congenital heart disease and more than 1 type of congenital syndrome

[e]Details of "Other" lesions are provided in Schlapbach 2022.[10]

[f]Details of "Other" congenital syndromes are provided in Schlapbach 2022.[10]

[g]Low cardiac output syndrome was defined as a blood lactate level greater than 4 mmol/L with a concurrent oxygen extraction gradient of at least 35 percentage points, or high inotrope and/or vasopressor requirements

© 2025 Long DA et al. *JAMA Network Open*.

eTable 2. Additional Sociodemographic Characteristics of Children and Their Parents/Caregivers Who Participated in NITRIC 12-Month Follow-Up

Characteristic	Nitric oxide N=462	Standard care N=465	Difference (95% CI)
**Number of people in house on regular basis** *n (%)*			
2	20 (4.3)	29 (6.2)	1.9 (-1.0 to 4.8)
3	139 (30.1)	125 (26.9)	-3.2 (-9.0 to 2.6)
4	157 (34.0)	180 (38.7)	4.7 (-1.5 to 10.9)
5	71 (15.4)	79 (17.0)	1.6 (-3.1 to 6.4)
6	33 (7.1)	18 (3.9)	-3.3 (-6.2 to -0.3)
7 or more	30 (6.5)	22 (4.7)	-1.8 (-4.7 to 1.2)
Missing	12 (2.6)	12 (2.6)	
**Types of people who live in house**[a]			
Your children *n (%)*	414 (89.6)	405 (87.1)	-2.8 (-6.6 to 1.0)
Your partner *n (%)*	361 (78.1)	360 (77.4)	-0.9 (-6.2 to 4.3)
Your/Partner's parents *n (%)*	48 (10.4)	49 (10.5)	1.3 (-3.9 to 4.2)
Extended family *n (%)*	32 (6.9)	30 (6.5)	0.5 (-3.8 to 2.8)
Your partner's children *n (%)*	22 (4.8)	18 (3.9)	-0.9 (-3.6 to 1.8)
Friends/Guests *n (%)*	8 (1.7)	9 (1.9)	0.2 (-1.6 to 2.0)
Other *n (%)*[b]	0 (0)	1 (0.2)	-0.2 (-0.2 to 0.7)
Missing	12 (2.6)	11 (2.4)	
**Number of children < 18 years living in house** *n (%)*			
1	169 (36.6)	174 (37.4)	0.8 (-5.4 to 7.1)
2	163 (35.3)	177 (38.1)	2.8 (-3.4 to 9.0)
3	73 (15.8)	69 (14.8)	-1.0 (-5.6 to 3.7)
4	27 (5.8)	16 (3.4)	-2.4 (-5.1 to 0.3)
5 or more	17 (3.7)	16 (3.4)	-0.2 (-2.6 to 2.1)
Missing	13 (2.8)	13 (2.8)	
**Partner Highest Education** *n (%)*	*N=418*	*N=408*	
Diploma/Community College Trade	157 (37.6)	162 (39.7)	2.1 (-4.5 to 8.8)
Bachelor	99 (23.7)	89 (21.8)	-1.9 (-7.6 to 3.8)
High School	92 (22.0)	94 (23.0)	1.0 (-4.7 to 6.7)
Postgraduate	53 (12.7)	46 (11.3)	-1.4 (-5.8 to 3.0)
Primary School	1 (0.2)	2 (0.5)	0.3 (-0.6 to 1.1)
Other[b]	2 (0.5)	1 (0.3)	-0.2 (-1.1 to 0.6)
Missing	14 (3.4)	14 (3.4)	
**Partner Employment Status** *n (%)*	*N=420*	*N=418*	
Full time	310 (73.8)	320 (76.6)	2.7 (-3.1 to 8.6)
Part time	41 (9.8)	38 (9.1)	-0.7 (-4.6 to 3.3)
Stay at home parent	29 (6.9)	36 (8.6)	1.7 (-1.9 to 5.3)
Other[b]	25 (6.0)	10 (2.4)	-3.6 (-6.3 to -0.9)
Missing	15 (3.6)	14 (3.4)	

© 2025 Long DA et al. *JAMA Network Open*.

[a]Patients may have more than 1 type of people living in the house

[b]Details of “Other” type of people living in the house, partner highest education and partner employment status are provided in eTable 3 of Supplement.

© 2025 Long DA et al. *JAMA Network Open*.

eTable 3. Listing of Sociodemographic Data Contained in "Other" Categories. Please refer to Table 2 for the comparison of sociodemographic data between the intervention and the control group

Description	Frequency
**Respondent Employment Status**	
Student	13
Actively seeking employment	11
Causal	8
Self employed	7
Unemployed	2
**Types of people who live in house**	
Not stated	1
**Partner Highest Education**	
Not stated	3
**Partner Employment Status**	
Student	7
Actively seeking employment	7
Causal	5
Self employed	10
Unemployed	6
**Languages Spoken at Home**	
Mandarin	16
Filipino	15
Hindi	14
Samoan/Tongan/Tole lauan	13
Arabic	10
Punjabi	10
Spanish	8
Cantonese	7
French	7
Singhalese	7
Malaysian	6
Urdu	6
Bengali	5
Nepali	5
Tamil	5
Afrikaans	<5
Amharic	<5
Bosnian	<5
Creole	<5
Croatian	<5
Dzongkha	<5
Egyptian	<5
Estonian	<5

© 2025 Long DA et al. *JAMA Network Open*.

Fijian	<5
Finnish	<5
First nation indigenous	<5
German	<5
Greek	<5
Gujarati	<5
Hokkien	<5
Igbo	<5
Indonesian	<5
Italian	<5
Japanese	<5
Kannada	<5
Kashmiri	<5
Korean	<5
Lebanese	<5
Macedonian	<5
Marathi	<5
Pashto	<5
Polish	<5
Portuguese	<5
Romanian	<5
Russian	<5
Serbian	<5
Sign language	<5
Somali	<5
Swahili	<5
Telugu	<5
Thai	<5
Tigrinya	<5
Turkish	<5
Vietnamese	<5

© 2025 Long DA et al. *JAMA Network Open*.

eTable 4. Sensitivity Analysis for Comparison of Primary and Secondary Outcomes

Outcome	Nitric Oxide	Standard Care	Unadjusted Estimate of Difference (95% CI)	Adjusted Estimate of Difference (95% CI)
ASQ-3 Total Score[a] *mean (SD)*	192.6 (78.4) *(N = 251)*	197.8 (75.8) *(N = 228)*	-0.54 (-12.86 to 11.79)	-2.59 (-9.42 to 14.59)
ASQ-3 Domain Score[b] *mean (SD)*				
Communication	33.9 (18.9) *(N = 251)*	35.6 (19.6) *(N = 230)*	-1.37 (-4.37 to 1.63)	-0.83 (-4.10 to 2.45)
Gross Motor Score	38.8 (22.8) *(N = 251)*	39.0 (21.8) *(N = 229)*	0.95 (-2.62 to 4.53)	0.05 (-3.64 to 3.73)
Fine Motor Score	41.1 (17.1) *(N = 252)*	41.8 (16.2) *(N = 230)*	0.41 (-2.31 to 3.14)	0.60 (-2.28 to 3.48)
Problem solving	38.2 (17.5) *(N = 251)*	39.2 (16.7) *(N = 230)*	0.08 (-2.66 to 2.82)	-0.06 (-2.85 to 2.97)
Personal Social	40.5 (15.6) *(N = 252)*	42.4 (15.2) *(N = 228)*	-0.61 (-3.13 to 1.90)	-0.77 (-3.36 to 1.83)
Modified Pediatric Overall Performance Category[c]	*(N = 388)*	*(N = 386)*		
Good/normal Functionally normal *n (%)*	265 (68.3)	284 (73.6)	*1 (reference)*	*1 (reference)*
Mild overall disability *n (%)*	66 (17.0)	43 (11.1)	0.50 (0.08 to 0.92)	0.52 (0.08 to 0.95)
Moderate/Severe overall disability *n (%)*	57 (14.7)	59 (15.3)	0.03 (-0.37 to 0.44)	0.01 (-0.48 to 0.50)
Coma/vegetative state *n (%)*	-	-	-	-
Brain death *n (%)*	-	-	-	-

[a]Adjusted for age at randomization, lesion type and site, ASQ-3 total score at baseline

[b]Adjusted for age at randomization, lesion type and site, ASQ-3 domain score at baseline

[c]Adjusted for age at randomization, lesion type and site, presurgical Modified Pediatric Overall Performance Category

© 2025 Long DA et al. *JAMA Network Open*.

eTable 5. Adjusted Estimates of Difference for Total ASQ-3 Score and Total PedsQL

Group	Nitric oxide	Standard care	Estimate (95% CI)
ASQ-3	N=458	N=463	
Age at randomization (weeks)[a] *mean (SD)*			*p=0.580*
< 6 weeks	201.9 (70.0) (N=162)	207.4 (67.2) (N=166)	-6.03 (-20.58 to 8.52)
≥ 6 weeks	193.7 (78.1) (N=296)	193.9 (77.0) (N=297)	-0.18 (-12.65 to 12.28)
Lesion[b] *mean (SD)*			*p=0.925*
Univentricular	188.5 (72.5) (N=49)	192.7 (65.3) (N=59)	-1.95 (-27.39 to 23.50)
Biventricular	197.6 (75.7) (N=409)	199.6 (75.0) (N=404)	-2.08 (-12.40 to 8.24)
PedsQL	N=454	N=455	
Age at randomization (weeks)[a] *mean (SD)*			*p=0.652*
< 6 weeks	74.7 (13.5) (N=158)	75.9 (13.7) (N=160)	-1.38 (-4.35 to 1.59)
≥ 6 weeks	74.0 (16.1) (N=296)	74.4 (15.1) (N=295)	-0.49 (-2.99 to 2.00)
Lesion[b] *mean (SD)*			*p=0.414*
Univentricular	68.4 (15.8) (N=49)	71.5 (14.4) (N=58)	-2.07 (-7.49 to 3.35)
Biventricular	75.0 (15.1) (N=405)	75.4 (14.6) (N=397)	-0.52 (-2.57 to 1.53)

[a]Adjusted for lesion type and site

[b]Adjusted for age at randomization and site

© 2025 Long DA et al. *JAMA Network Open*.

eTable 6. Factors Associated With Neurodevelopmental Outcome (ASQ-3 Total Score at 12 Months) (N=921)

Factors	ASQ-3 Total Score		Unadjusted Estimate of Difference (95% CI)[a]	Adjusted Estimate of Difference (95% CI)[b] (N=890)
	n	mean (SD)		
**Child**				
Gestation at birth				
<37 weeks	168	169.9 (82.9)	-34.0 (-46.2 to -21.7)	-17.9 (-28.9 to -6.9)
≥37 weeks	753	203.9 (71.2)	*1 (reference)*	*1 (reference)*
Age at randomization (weeks)				
< 6	328	204.7 (68.6)	10.9 (0.9 to 20.9)	7.8 (-1.6 to 17.1)
≥ 6	593	193.8 (77.5)	*1 (reference)*	*1 (reference)*
Congenital heart disease *n (%)*				
Univentricular	108	190.8 (68.4)	-7.8 (-22.7 to 7.2)	-16.0 (-29.3 to -2.6)
Biventricular	813	198.6 (75.4)	*1 (reference)*	*1 (reference)*
Congenital syndrome				
No	770	213.5 (61.9)	*1 (reference)*	*1 (reference)*
Yes	151	117.1 (81.5)	-97.1 (-108.6 to -85.7)	-91.2 (-102.8 to -79.6)
**Sociodemographic**				
Parent highest education				
High School	209	195.1 (75.5)	-12.6 (-28.0 to 2.9)	-11.3 (-24.8 to 2.2)
Diploma/TAFE/Trade	273	197.2 (76.0)	-10.4 (-25.1 to 4.2)	-9.2 (-21.8 to 3.4)
Bachelor	254	194.8 (71.7)	-12.8 (-27.7 to 2.0)	-8.7 (-21.4 to 4.0)
Postgraduate	154	207.6 (73.8)	*1 (reference)*	*1 (reference)*
Ethnicity				
Aboriginal/Torres Strait Islander	17	192.9 (102.6)	-4.8 (-40.7 to 31.0)	-3.7 (-36.0 to 28.6)
Asian	102	191.2 (77.3)	-6.5 (-22.1 to 9.0)	-8.2 (-21.9 to 5.6)
Māori/Pacific Islander Peoples	67	197.5 (83.7)	0.2 (-19.0 to 18.5)	3.2 (-14.8 to 21.2)
Multiethnic/Other	101	204.9 (67.7)	7.2 (-8.4 to 22.9)	-0.8 (-14.3 to 12.7)
White	634	197.7 (73.5)	*1 (reference)*	*1 (reference)*
**Treatment and Surgery**				
NITRIC trial treatment group				
Nitric oxide	458	196.6 (75.4)	-2.1 (-11.7 to 7.5)	-2.1 (-10.4 to 6.1)
Standard care	463	198.7 (73.8)	*1 (reference)*	*1 (reference)*
Duration of cardiopulmonary bypass (mins)[c]	921	0.019	0 (-0.1 to 0.1)	-
RACHS score				
RACHS-1 or RACHS-2	461	198.3 (74.4)	*1 (reference)*	-
RACHS-3	246	192.3 (79.5)	-6.0 (-17.5 to 5.5)	-

© 2025 Long DA et al. *JAMA Network Open*.

Factors	ASQ-3 Total Score		Unadjusted Estimate of Difference (95% CI)[a]	Adjusted Estimate of Difference (95% CI)[b] (N=890)
	n	mean (SD)		
RACHS-4	178	203.8 (68.8)	5.5 (-7.4 to 18.4)	-
RACHS-5 or RACHS-6	36	195.6 (69.8)	-2.8 (-28.0 to 22.5)	-
Cross clamp (mins)[c]	863	0.032	0 (-0.1 to 0.1)	-
Deep hypothermic arrest				
No	835	197.4 (74.7)	*1 (reference)*	-
Yes	86	200.4 (73.4)	3.0 (-13.5 to 19.6)	-
Antegrade cerebral perfusion				
No	771	197.1 (75.2)	*1 (reference)*	-
Yes	150	200.3 (71.3)	3.1 (-9.9 to 16.1)	-
**ICU Management and Outcomes**				
Low cardiac output syndrome[d]				
No	746	200.3 (73.8)	*1 (reference)*	*1 (reference)*
Yes	175	186.7 (76.9)	-13.6 (-25.8 to -1.4)	-3.6 (-15.2 to 7.9)
Duration of invasive ventilation (days)[c,e]	921	-0.132	-8.6 (-12.5 to -4.6)	-
Extracorporeal life support				
No	910	198.2 (74.1)	*1 (reference)*	-
Yes	11	150.9 (98.3)	-47.3 (-91.5 to -3.1)	-
Seizures during ICU admission				
No	909	197.9 (74.6)	*1 (reference)*	-
Yes	12	181.7 (72.1)	-16.2 (-58.7 to 26.2)	-
Length of stay in ICU (days)[c,e]	921	-0.178	-15.2 (-20.4 to -10.0)	-2.4 (-3.2 to -1.6)

Abbreviations: CPB, cardiopulmonary bypass; ICU, intensive care unit; RACHS, risk adjustment in congenital heart surgery.

[a]Adjusted for site

[b]Adjusted for site, prematurity, age at randomization, univentricular/biventricular disease, congenital syndrome, parental highest education, ethnicity, study treatment group, low cardiac output syndrome, and length of stay in ICU; RACHS was excluded due to collinearity

[c]Spearman's correlation coefficient

[d]Low cardiac output syndrome was defined as a blood lactate level greater than 4 mmol/L with a concurrent oxygen extraction gradient of at least 35 percentage points, or high inotrope and/or vasopressor requirement.

[e]Independent variable log-transformed in bivariate and multivariate analyses to meet model assumptions.

© 2025 Long DA et al. *JAMA Network Open*.

eTable 7. Factors Associated With Health-Related Quality of Life Outcome (PedsQL Total Score at 12 Months) (N=909)

Factors	PedsQL Total Score		Unadjusted Estimate of Difference (95% CI)[a]	Adjusted Estimate of Difference (95% CI)[b] (N=893)
	n	mean (SD)		
**Child**				
Gestation at birth				
<37 weeks	165	69.2 (15.9)	-6.6 (-9.1 to -4.2)	-5.3 (-7.7 to -2.8)
≥37 weeks	747	75.8 (14.5)	*1 (reference)*	*1 (reference)*
Age at randomization (weeks)				
< 6	318	75.3 (13.6)	1.1 (-1.0 to 3.1)	-0.8 (-1.3 to 2.8)
≥ 6	591	74.2 (15.6)	*1 (reference)*	*1 (reference)*
Congenital heart disease *n (%)*				
Univentricular	107	70.0 (15.1)	-5.2 (-8.2 to -2.2)	-6.2 (-9.1 to -3.2)
Biventricular	802	75.2 (14.8)	*1 (reference)*	*1 (reference)*
Congenital syndrome				
No	759	76.3 (14.4)	*1 (reference)*	*1 (reference)*
Yes	150	66.2 (15.1)	-10.1 (-12.6 to -7.5)	-9.4 (-12.0 to -6.8)
**Sociodemographic**				
Parent highest education				
High School	209	74.5 (13.7)	-1.1 (-4.1 to 2.0)	-0.5 (-3.5 to 2.5)
Diploma/TAFE/Trade	273	73.8 (16.3)	-1.8 (-4.7 to 1.2)	-1.4 (-4.2 to 1.4)
Bachelor	255	75.1 (13.6)	-0.4 (-3.4 to 2.5)	0.4 (-2.4 to 3.2)
Postgraduate	156	75.5 (15.8)	*1 (reference)*	*1 (reference)*
Ethnicity				
Aboriginal/Torres Strait Islander	17	72.1 (16.9)	-2.6 (-9.8 to 4.6)	-2.2 (-9.4 to 5.0)
Asian	100	74.3 (16.3)	-0.4 (-3.5 to 2.8)	-0.6 (-3.6 to 2.5)
Māori/Pacific Islander Peoples	67	74.3 (14.3)	-0.3 (-4.1 to 3.4)	0.8 (-3.3 to 5.0)
Multiethnic/Other	98	75.1 (14.7)	0.5 (-2.7 to 3.7)	-0.7 (-3.8 to 2.3)
White	627	74.7 (14.8)	*1 (reference)*	*1 (reference)*
**Treatment and Surgery**				
NITRIC trial treatment group				
Nitric oxide	454	74.2 (15.3)	-0.7 (-2.7 to 1.2)	-0.8 (-2.7 to 1.0)
Standard care	455	75.0 (14.6)	*1 (reference)*	*1 (reference)*
Duration of cardiopulmonary bypass (mins)[c]	909	-0.049	-0.01 (-0.03 to -0.001)	-
RACHS score				

© 2025 Long DA et al. *JAMA Network Open*.

Factors	PedsQL Total Score		Unadjusted Estimate of Difference (95% CI)[a]	Adjusted Estimate of Difference (95% CI)[b] (N=893)
	n	mean (SD)		
RACHS-1 or RACHS-2	456	74.6 (15.3)	*1 (reference)*	-
RACHS-3	242	74.2 (15.2)	-0.4 (-2.7 to 1.9)	-
RACHS-4	176	75.9 (13.2)	1.3 (-1.3 to 3.9)	-
RACHS-5 or RACHS-6	35	70.5 (16.1)	-4.1 (-9.3 to 1.0)	-
Cross clamp (mins)[c]	850	-0.014	-0.01 (-0.03 to 0.01)	-
Deep hypothermic arrest				
No	823	74.4 (15.1)	*1 (reference)*	-
Yes	86	76.3 (13.9)	1.9 (-1.4 to 5.2)	-
Antegrade cerebral perfusion				
No	759	74.7 (15.1)	*1 (reference)*	-
Yes	150	74.3 (14.1)	-0.4 (-3.0 to 2.2)	-
**ICU Management and Outcomes**				
Low cardiac output syndrome[d]				
No	733	75.0 (14.8)	*1 (reference)*	*1 (reference)*
Yes	176	72.9 (15.3)	-2.1 (-4.6 to 0.3)	-0.6 (-3.1 to 1.9)
Duration of invasive ventilation (days)[c,e]	909	-0.130	-1.6 (-2.4 to -0.7)	-
Extra corporeal life support				
No	898	74.7 (14.8)	*1 (reference)*	-
Yes	11	67.7 (22.2)	-7.0 (-15.9 to 1.9)	-
Seizures during ICU admission				
No	897	74.7 (14.9)	*1 (reference)*	-
Yes	12	69.5 (21.0)	-5.2 (-13.7 to 3.4)	-
Length of stay in ICU (days)[c,e]	909	-0.159	-2.6 (-3.7 to -1.6)	-0.3 (-0.4 to -0.1)

Abbreviations: CPB, cardiopulmonary bypass; ICU, intensive care unit; RACHS, risk adjustment in congenital heart surgery.

[a]Adjusted for site

[b]Adjusted for site, prematurity, age at randomization, univentricular/biventricular disease, congenital syndrome, parental highest education, ethnicity, study treatment group, low cardiac output syndrome, and length of stay in ICU; RACHS was excluded due to collinearity

[c]Spearman's correlation coefficient

[d]Low cardiac output syndrome was defined as a blood lactate level greater than 4 mmol/L with a concurrent oxygen extraction gradient of at least 35 percentage points, or high inotrope and/or vasopressor requirement.

[e]Independent variable log-transformed in bivariate and multivariate analyses to meet model assumptions.

© 2025 Long DA et al. *JAMA Network Open*.
