# Supplementary material for: Neurodevelopmental Outcomes After Nitric Oxide During Cardiopulmonary Bypass for Open Heart Surgery: A Randomized Clinical Trial
Source: JAMA Netw Open. 2025 Feb 5;8(2):e2458040. doi: 10.1001/jamanetworkopen.2024.58040 (PMC11800016; doi:10.1001/jamanetworkopen.2024.58040)
Supplement: Supplement 3. — Nonauthor Collaborators [file jamanetwopen-e2458040-s003.pdf]

Supplemental Online Content: Nonauthor Collaborators
*First name, last name, and suffix (if applicable) are required and will appear in PubMed.

*Group Name(s): the NITRIC Study Group							
***First Name and Middle Initial(s)**	***Last Name**	***Suffix (eg, Jr, III)**	Academic Degrees	Institution	Location (city, state/province, country)	Role or Contribution, eg, chair, principal investigator	Group (if more than 1 Group listed in the byline) and/or Subgroup (eg, Steering Committee)
Johnny	Millar		MBChB, PhD	Royal Children's Hospital	Melbourne, Australia	Chief Investigator	**NITRIC Study Group**
Carmel	DelZoppo			Royal Children's Hospital	Melbourne, Australia	Research Coordinator	**NITRIC Study Group**
John	Beca			Starship Children's Hospital	Auckland, New Zealand	Chief Investigator	**NITRIC Study Group**
Taryn	Evans			Starship Children's Hospital	Auckland, New Zealand	Perfusionist	**NITRIC Study Group**
Shelley	Coetzer			Starship Children's Hospital	Auckland, New Zealand	Research Coordinator	**NITRIC Study Group**
Claire	Sherring			Starship Children's Hospital	Auckland, New Zealand	Research Coordinator	**NITRIC Study Group**
Killian	O'Shaughnessy			The Children's Hospital at Westmead	Sydney, Australia	Perfusionist	**NITRIC Study Group**
Chong Tien	Goh			The Children's Hospital at Westmead	Sydney, Australia	PICU Specialist	**NITRIC Study Group**
Gail	Harper			The Children's Hospital at Westmead	Sydney, Australia	Research Coordinator	**NITRIC Study Group**
Sam	Barr		BSc	Perth Children's Hospital	Perth, Australia	Study procedure, set u	**NITRIC Study Group**
Rae	Kelly		MSc (Perfusion)	Perth Children's Hospital	Perth, Australia	Perfusionist	**NITRIC Study Group**
Hannah	Thomson		BSc, PGCert	Perth Children's Hospital	Perth, Australia	Site study coordinator	**NITRIC Study Group**
Kelly	Holmes		DipNursing (UK)	Perth Children's Hospital	Perth, Australia	Cardiac liaison, recruitr	**NITRIC Study Group**
Nigel	Slade		Diploma in Clinical Perfusion	Perth Children's Hospital	Perth, Australia	Perfusionist	**NITRIC Study Group**
David	Andrews		MBBS	Perth Children's Hospital	Perth, Australia	Recruitment and conse	**NITRIC Study Group**
Carla	Zazulak			Queensland Children's Hospital	Brisbane, Australia	Perfusionist	**NITRIC Study Group**
Benjamin	Anderson			Queensland Children's Hospital	Brisbane, Australia	Cardiologist	**NITRIC Study Group**
Jessicah	Minogue			Queensland Children's Hospital	Brisbane, Australia	Research Coordinator	**NITRIC Study Group**
Antje	Blumenthal			Diamantina Institute, The University of	Brisbane, Australia	Immunology and micro	**NITRIC Study Group**
Jonas	Fooken		BA, MA, PhD	School of Economics, and Centre for th	Brisbane, Australia	Health Economist	**NITRIC Study Group**
Endrias	Ergetu		BBus, BEng (Hons), MEngSci	The University of Queensland	Brisbane, Australia	Data analyst	**NITRIC Study Group**
Brenda	Gannon			School of Economics, and Centre for th	Brisbane, Australia	Health Economist	**NITRIC Study Group**
Trang	Pham		BEng, MEng	The University of Queensland	Brisbane, Australia	Data Manager	**NITRIC Study Group**
Annelies	Hennick			Wilhelmina Children’s Hospital	Utrecht, The Netherlands	Trial coordinator, data	**NITRIC Study Group**

Supplemental Online Content: Nonauthor Collaborators
*First name, last name, and suffix (if applicable) are required and will appear in PubMed.

***First Name and Middle Initial(s)**	***Last Name**	***Suffix (eg, Jr, III)**	Academic Degrees	Institution	Location (city, state/province, country)	Role or Contribution, eg, chair, principal investigator	Group (if more than 1 Group listed in the byline) and/or Subgroup (eg, Steering Committee)
Nicole	van Bell-van Haaren		MSc	Wilhelmina Children’s Hospital	Utrecht, The Netherlands	Preparations for implementation of the intervention and data collection perfusion	**NITRIC Study Group**
Bram	van Wijk			Wilhelmina Children’s Hospital	Utrecht, The Netherlands	Surgical data collection and scoring	**NITRIC Study Group**
Erik	Koomen		MD, PhD	Wilhelmina Children’s Hospital	Utrecht, The Netherlands	Sub-PI, PICU adverse event assessor and data collection	**NITRIC Study Group**
Breanna	Pellegrini			Australian and New Zealand Paediatric Intensive Care Registry			**ANZICS PSG**
Shane	George			Gold Coast University Hospital	Gold Coast, Australia		**ANZICS PSG**
Corrine	Balit			John Hunter Hospital	Newcastle, Australia		**ANZICS PSG**
Felix	Oberender			Monash Children's Hospital	Melbourne, Austalia		**ANZICS PSG**
Simon	Erickson			Perth Children's Hospital	Perth, Australia		**ANZICS PSG**
Jenipher	Chubes Flores			Perth Children's Hospital	Perth, Australia		**ANZICS PSG**
Karina	Charles			Queensland Children's Hospital	Brisbane, Australia		**ANZICS PSG**
Sai	Raman			Queensland Children's Hospital	Brisbane, Australia		**ANZICS PSG**
Michaela	Waak			Queensland Children's Hospital	Brisbane, Australia		**ANZICS PSG**
Tara	Williams			Queensland Children's Hospital	Brisbane, Australia		**ANZICS PSG**
Debbie	Long			Queensland University of Technology	Brisbane, Australia		**ANZICS PSG**
Warwick	Butt			Royal Children's Hospitaal	Melbourne, Austalia		**ANZICS PSG**
Ben	Gelbart			Royal Children's Hospitaal	Melbourne, Austalia		**ANZICS PSG**
Kate	Masterson			Royal Children's Hospitaal	Melbourne, Austalia		**ANZICS PSG**
Johnny	Milllar			Royal Children's Hospitaal	Melbourne, Austalia		**ANZICS PSG**
Anusha	Ganeshalingam			Starship Children's Hospital	Auckland, New Zealand		**ANZICS PSG**
Claire	Sherring			Starship Children's Hospital	Auckland, New Zealand		**ANZICS PSG**
Puneet	Singh			Sydney Children's Hospital	Sydney, Australia		**ANZICS PSG**
Vicky	Smith			Sydney Children's Hospital	Sydney, Australia		**ANZICS PSG**
Kristen	Gibbons			The University of Queensland	Brisbane, Australia		**ANZICS PSG**
Luregn	Schlapbach			The University of Queensland	Brisbane, Australia		**ANZICS PSG**
Jessica	Schults			The University of Queensland	Brisbane, Australia		**ANZICS PSG**

Supplemental Online Content: Nonauthor Collaborators
*First name, last name, and suffix (if applicable) are required and will appear in PubMed.

***First Name and Middle Initial(s)**	***Last Name**	***Suffix (eg, Jr, III)**	Academic Degrees	Institution	Location (city, state/province, country)	Role or Contribution, eg, chair, principal investigator	Group (if more than 1 Group listed in the byline) and/or Subgroup (eg, Steering Committee)
Jennifer	Darvas			The Children's Hospital at Westmead	Sydney, Australia		**ANZICS PSG**
Marino	Festa			The Children's Hospital at Westmead	Sydney, Australia		**ANZICS PSG**
