# Supplementary material for: Neurodevelopmental Outcomes After Nitric Oxide During Cardiopulmonary Bypass for Open Heart Surgery: A Randomized Clinical Trial
Source: JAMA Netw Open. 2025 Feb 5;8(2):e2458040. doi: 10.1001/jamanetworkopen.2024.58040 (PMC11800016; doi:10.1001/jamanetworkopen.2024.58040)
Supplement: Supplement 4. — Data Sharing Statement [file jamanetwopen-e2458040-s004.pdf]

Data Sharing Statement

Long. Neurodevelopmental Outcomes After Nitric Oxide During Cardiopulmonary Bypass for Open Heart Surgery. *JAMA Netw Open*. Published February 05, 2025.
doi:10.1001/jamanetworkopen.2024.58040

Data
Additional Information: Australian and New Zealand Clinical Trials Registry anzctr.org.au ACTRN12617000821392
Data available: Yes
Data types: Deidentified participant data
How to access data: Deidentified participant data will be shared upon reasonable request, addressed to NITRIC Study Group at warwick.butt@rch.org.au. Researchers with an approved proposed use, approved by appropriate institutional review boards and the Trial Steering Committee. Types of analyses: An approved specified purpose. Mechanisms of data availability: With a signed data access agreement. How to access data: Deidentified participant data will be shared upon reasonable request, addressed to the NITRIC Study Group at warwick.butt@rch.org.au.
When available: With publication

Supporting Documents
Document types: Statistical/analytic code
How to access documents: The statistical analysis plan has been published before completion of recruitment (Gibbons et al. Crit Care Resusc 2021; 23 (1): 47-58). This publication provides the link to the GitHub repository where the full Stata code was uploaded before completion of recruitment: https://github.com/kgibbons44/NITRICAnalysis/
When available: With publication

Additional Information
Who can access the data: Researchers with an approved proposed use, approved by appropriate institutional review boards and the Trial Steering Committee.
Types of analyses: An approved specified purpose.
Mechanisms of data availability: With a signed data access agreemen
Any additional restrictions: Any data release needs to be approved by the NITRIC Study Group, the Australian and New Zealand Intensive Care Society Clinical Trials Group (ANZICS CTG), and the ANZICS Paediatric Study Group (PSG)
